# A copy number variation in human NCF1 and its pseudogenes

**DOI:** 10.1186/1471-2156-11-13

**Published:** 2010-02-23

**Authors:** Tiffany Brunson, Qingwei Wang, Isfahan Chambers, Qing Song

**Affiliations:** 1 Cardiovascular Research Institute, Morehouse School of Medicine, Atlanta, Georgia, USA

## Abstract

**Background:**

Neutrophil cytosolic factor-1 (NCF1) is a component of NADPH oxidase. The NCF1 gene colocalizes with two pseudogenes (NCF1B and NCF1C). These two pseudogenes have a GT deletion in exon 2, resulting in a frameshift and an early stop codon. Here, we report a copy number variation (CNV) of the NCF1 pseudogenes and their alternative spliced expressions.

**Results:**

We examined three normal populations (86 individuals). We observed the 2:2:2 pattern (NCF1B:NCF1:NCF1C) in only 26 individuals. On average, each African- American has 1.4 ± 0.8 (Mean ± SD) copies of NCF1B and 2.3 ± 0.6 copies of NCF1C; each Caucasian has 1.8 ± 0.7 copies of NCF1B and 1.9 ± 0.4 copies of NCF1C; and each Mexican has 1.6 ± 0.6 copies of NCF1B and 1.0 ± 0.4 copies of NCF1C. Mexicans have significantly less NCF1C copies than African-Americans (*p *= 6e-15) and Caucasians (*p *= 3e-11). Mendelian transmission of this CNV was observed in two CEPH pedigrees. Moreover, we cloned two alternative spliced transcripts generated from these two pseudogenes that adopt alternative exon-2 instead of their defective exon 2. The NCF1 pseudogene expression responded robustly to PMA induction during macrophage differentiation. NCF1B decreased from 32.9% to 8.3% in the cDNA pool transcribed from 3 gene copies. NCF1Ψs also displayed distinct expression patterns in different human tissues.

**Conclusions:**

Our results suggest that these two pseudogenes may adopt an alternative exon-2 in different tissues and in response to external stimuli. The GT deletion is insufficient to define them as functionless pseudogenes; this CNV may have biological relevance.

## Background

Recent genomic studies suggested that gene duplication occurred frequently and in variable numbers during the recent history of human populations, which has led to de novo formations of copy number variation (CNV) [1]. Presumably due to positive selection, genes encoding certain protein categories are particularly enriched in CNVs, such as those involved in processes related to environmental responses [1-8]. In this process, duplicated genes are thought to be the "successful" copies; pseudogenes are those "unsuccessful" duplicates retained in the genome [1].

Neutrophil cytosolic factor 1 (NCF1, also called p47^*phox*^, for phagocyte oxidase), is a crucial component of NADPH oxidase [9]. This enzyme catalyzes the production of microbicidal superoxide in phagocytes such as neutrophil and plays a vital role in host defense against microbial pathogens [10,11]. A 2-bp GT deletion in exon 2 of the NCF1 gene causes chronic granulomatous disease (CGD) in humans [12,13]. NCF1 is expressed in many cell types and may play a role in many other diseases [14-19].

The human NCF1 gene is located at 7q11.23, the Williams Beurens Syndrome region [20-22], accompanied by two nearly identical (>99.5%) pseudogenes (NCF1B and NCF1C) which presumably arose by gene duplication [23]. These two pseudogenes have the same signature sequence as the one in the NCF1 gene responsible for CGD, the 2-bp GT deletion in exon 2 [13,23,24]. This mutation leads to a frameshift and a premature stop codon and thus these two gene duplicates were categorized as pseudogenes. It has been noticed that the NCF1Ψ/NCF1 ratio vary in human individuals, and it was believed some NCF1Ψ gene copies contain the wild-type NCF1 exon 2 sequence [22,25].

In this study, we examined the copy numbers of NCF1 pseudogenes in human populations and found a copy number variation (CNV). Our additional data revealed that these two pseudogenes can generate RNA transcripts that skip over their defective exon 2 by alternative splicing.

## Results

### Copy Number Variation of NCF1 and NCF1Ψ

**Figure 1:**
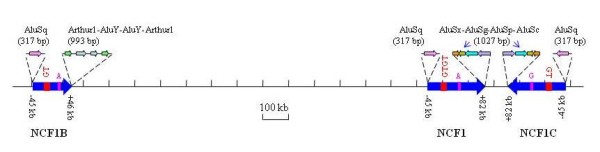
**Genomic organization of the NCF1 gene locus at 7q11.23 in the human genome**. There are 3 large duplicons (arrow blocks) at this locus; the arrow directions illustrate the NCF1 or NCF1Ψ transcription orientations. According to their genomic positions, we designated those two pseudogene duplicons as NCF1B and NCF1C. These duplicons share a 106-kb sequence with >99.5% similarities spanning from -45 kb at 5'-end to +46 kb at 3'-end regarding the NCF1/NCF1Ψ coding region. NCF1 and NCF1B duplicons share an additional 3'-flanking sequence until +82 kb. The signature difference between NCF1 and its pseudogenes, the 2-bp GT deletion, is showed by GTGT at NCF1 and GT at NCF1B and NCF1C. Another nucleotide difference between duplicons, an A/G substitution in exon-9, is also indicated above the arrow blocks. The transposons at the boundaries of these duplicons are indicated.

**Figure 2:**
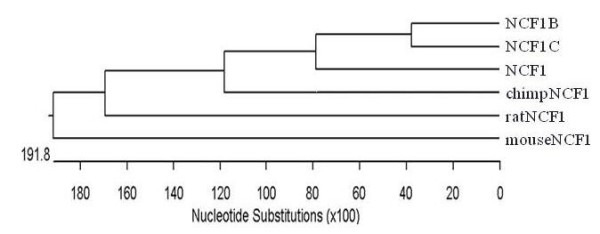
**Phylogenetic analysis of NCF1 and NCF1Ψ genes**.

There are three large highly homologous duplicons at the NCF1 locus (Figure 1). According to their chromosomal positions (UCSC Human Assembly 2006 March, chr7:72,272,547-72,287,915 [pseudogene], chr7:73,826,245-73,841,595 [NCF1], chr7:74,210,381-74,225,753 [pseudogene]), we designated two NCF1 pseudogene duplicons as NCF1B and NCF1C, respectively. These duplicons share a 106-kb sequence with >99.5% similarities spanning from -45 kb at 5'-end to +46 kb at 3'-end regarding the NCF1 coding region. NCF1 or NCF1C duplicons share an additional 3'-flanking sequence until +82 kb (Figure 1). In contrast to the human genome, NCF1 is a single-copy gene in the reference genomes of all other species, such as chimpanzee, rhesus monkey, rat and mouse (UCSC Genome Assemblies). The phylogenetic tree revealed that NCF1B and NCF1C duplicated after they arose from the NCF1 gene (Figure 2).

To determine the relative copy numbers of NCF1B, NCF1, and NCF1C, we genotyped the genomic DNA of human subjects at two particular positions, the signature 2-bp GT deletion in exon 2, and an A/G substitution in exon-9, in which NCF1B and NCF1 has an A allele, and NCF1C has a G allele (Figure 1 and Additional file 1). Pyrosequencing is a high-throughput technology that can be used for accurate determination of the allele frequency in pooled DNA [26]. Based on the pyrogram peak heights, we assessed the allele composition of each individual with the PyroMarkID Software v1.0 (Additional file 2).

**Figure 3:**
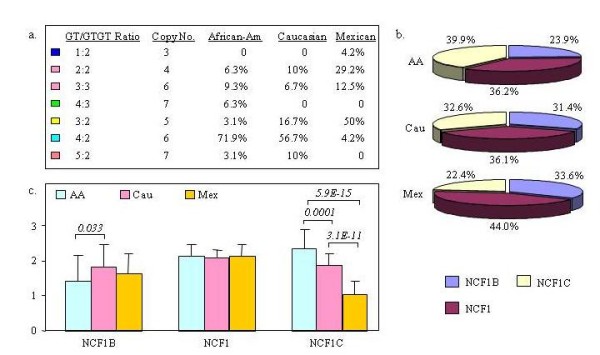
**Copy number variation at the NCF1 locus**. a) The ratio of NCF1 and NCF1Ψ genes as determined by pyrosequencing the GT deletion ([GT]/[GTGT]). b) The percentage of NCF1, NCF1B and NCF1C copies in the gene pool of each population. c) The average copy numbers of NCF1, NCF1B and NCF1C in each individual (mean ± SD). Three independent experiments were performed in duplicates. P values are indicated.

We analyzed 86 non-related individuals (32 African-Americans [AA], 30 Caucasians [Cau] and 24 Mexicans [Mex]). Totally we observed 6 different NCF1Ψ/NCF1 ratios in these normal populations (Figure 3a and Additional file 3). The 4:2 ratio (4 pseudogenes & 2 NCF1 in each genome) are predominant in AA (71.9%) and Caucasians (56.1%), but rare in Mexicans (4.2%), instead, the 3:2 (50.0%) and 2:2 (41.7%) genotypes are common in Mexicans. Interestingly, the Mexican population has an additional genotype (4.2%), 1:2, which contains only one pseudogene copy in each genome.

Using the genotyping pyrograms at exon 2 (GT/GTGT/GT) [NCF1B/NCF1/NCF1C] and exon 9 (A/A/G), we were able to further dissect the copy numbers of two pseudogenes in each genome (see Additional file 3). On average, each AA individual has 1.4 ± 0.8 (Mean ± SD) copies of NCF1B, 2.1 ± 0.7 copies of NCF1, and 2.3 ± 0.6 copies of NCF1C; each Caucasian has 1.8 ± 0.7 copies of NCF1B, 2.1 ± 0.3 copies of NCF1, and 1.9 ± 0.4 copies of NCF1C; and each Mexican genome has 1.6 ± 0.6 copies of NCF1B, 2.1 ± 0.3 copies of NCF1, and 1.0 ± 0.4 copies of NCF1C. There is a significant difference on the copy number of NCF1C among these 3 human populations (Figure 3c and 3d), in which Mexicans have significantly less copies of NCF1C than AA (*p *= 6e-15) and Cau (*p *= 3e-11). There is also a significant difference on the copy number of NCF1B between Cau and AA (*p *= 0.033).

Epstein-Barr virus (EBV) transformed lymphoblastoid cells lines are widely used as a genomic resource for many human genetic studies. However, chromosomal instability, which can cause a duplication or deletion in the host or viral genome sequences flanking the integration sites, is increased by viral integration [27]. In order to eliminate the possibility that this CNV is just an artifact in lymphoblastoid cell lines, we analyzed 48 genomic DNA samples directly extracted from human peripheral white blood cells (Additional file 4). Collectively 4 individuals had 5:2 ratios (2.41+ 0.058), 18 individuals had 2:1 ratios (1.97+ 0.141), 23 individuals had 3:2 ratios (1.53+ 0.141) and 3 individuals had 1:1 ratios. This data confirmed the presence of this CNV.

### Heritability of NCF1 Copy Number Variation

**Figure 4:**
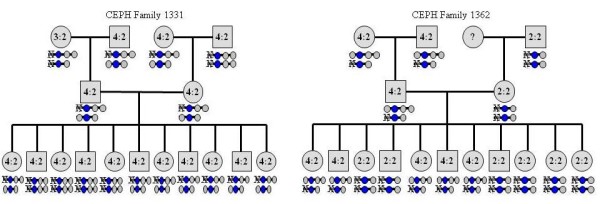
**Inheritance of the NCF1/NCF1Ψ copy numbers in two CEPH families**. A NCF1Ψ/NCF1 copy number ratio is indicated within each shape; haplogenotypes are indicated below each shape. In haplogenotypes, blue circles represent the NCF1 duplicons, grey circles represent the NCF1Ψ duplicons (NCF1B on the left arm of the NCF1 blue circle on the haplogenotype, NCF1C on the right arm of NCF1), and an X indicates an absence of NCF1 and NCF1Ψ copy.

Two large CEPH families were used to examine the heritability of this CNV detected at the NCF1 locus (Figure 4). The majority of the members in family 1331 have a 2:1 ΔGT/GTGT ratio as represented by a 4:2 ratio in a diploid genome, which was further determined as a 1:2:3 proportion (NCF1B: NCF1: NCF1C); one family member, the paternal grandmother, has a 0:2:3 ratio. Family 1362 is particularly interesting as both maternal and paternal lineages have very different NCF1B:NCF1:NCF1C ratios. The paternal lineage has an unvarying 4:2 ratio (1:2:3) where as the maternal lineage has a continuous 2:2 (0:2:2) ratio. Based on the potential haplogenotypes deduced from the pedigrees, we observed a clear allele transmission pattern of this CNV in both families, suggesting a heritability of this CNV; however, our data cannot exclude the de novo formation of new copy numbers of this CNV in other families. The clear inheritance of the copy numbers in these two large pedigrees also suggests that there is no large experimental bias on the copy number measurement.

### Transcription and Alternative Splicing of NCF1Ψ and NCF1 Genes

**Figure 5:**
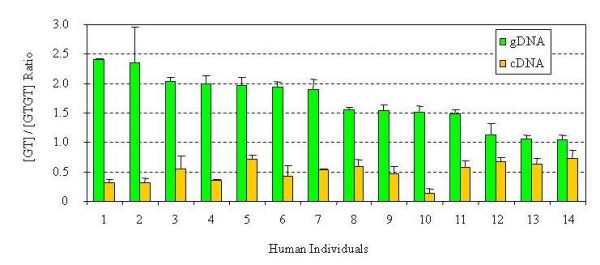
**Relative quantities of expression of NCF1 and its pseudogene**. The relative proportion of gene expression of NCF1 and NCF1Ψs (GTGT or GT containing transcripts) was measured by genotyping the cDNA samples from LCLs of 14 human individuals at the signature GT deletion. Two pseudogenes collectively contribute to lesser amount of transcript than from one NCF1 gene copy alone (0.3-0.7):1 [NCF1Ψ/NCF1, the [GT]/[GTGT] ratio) (yellow bars). The [GT]/[GTGT] ratios in genomic DNA are shown by the green bars. Three independent experiments were performed in duplicates.

We explored if these two NCF1 pseudogenes are transcriptionally active. Pyrosequencing was used to quantify the NCF1/NCF1Ψs compositions in the mRNAs of 14 single-donor lymphoblastoid cell lines by genotyping the signature 2-bp GT deletion in cDNA (Figure 5). Interestingly, although NCF1Ψs have more copies than NCF1 in each individual, they made much less GT-containing transcripts than NCF1. For example, in the individual-6 who has 4 copies of NCF1Ψs and 2 copies of NCF1, pseudogenes collectively only contributed the amount of transcripts equal to half of the amount from the NCF1 gene copy (as revealed by the ratio 0.5:1 in cDNA).

**Figure 6:**
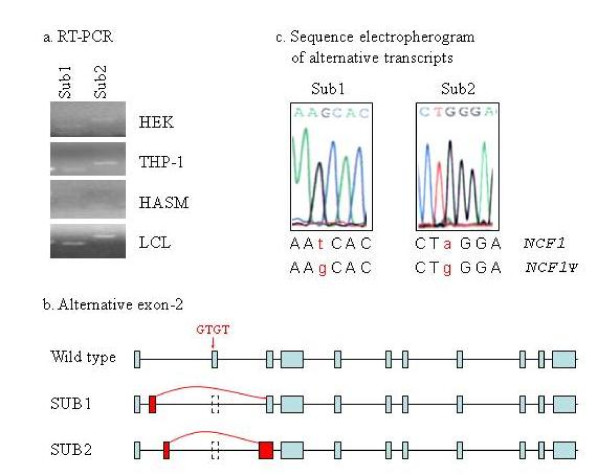
**Alternative splicing of the NCF1 pseudogenes**. a) RT-PCR detection of two novel pseudogene-specific exons. b) The alternative splicing pattern (sub1 and sub2) using these two new exons, both splicing isoforms skip over the defective exon 2. c) Sequencing electropherogram of sub1 and sub2, the single nucleotide substitutions between NCF1 and its pseudogenes revealed that these two alternative exons were transcribed from the pseudogene copies. HEK, human embryonic kidney cell line; THP-1, a monocyte cell line; HASM, human aortic smooth muscle cells; LCL, lymphoblastoid cell line.

By PCR, cloning and direct DNA sequencing, we have experimentally discovered two novel alternative exons (GenBank: GU215077, GU215078) located in the intron-1 (Additional file 5). Neither of these two new alternative splicing transcripts used the GT-containing exon-2 (Figure 6b), and both of them were made from the NCF1Ψs copies as revealed by their nucleotide sequences (Figure 6c). We have searched for putative open reading frames with the NCBI ORF Finder. Sub1 showed a continuous ORF without a stop codon, thus the full-length transcript containing this alternative splicing pattern may produce a long ORF. Sub2 contains three predicted ORF, but all have a stop codon before its last nucleotide (Additional file 6).

### NCF1Ψ and NCF1 Transcription in Monocyte Differentiation

**Figure 7:**
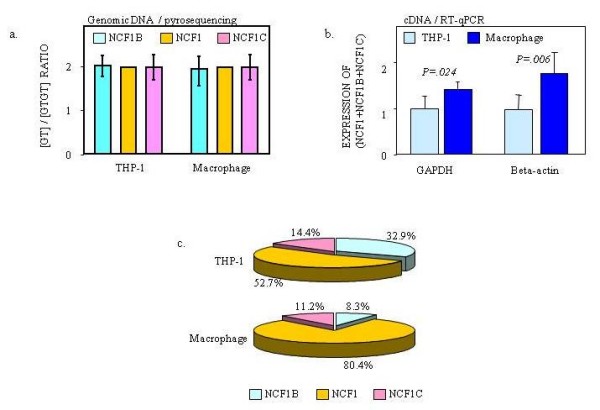
**The response of NCF1Ψ/NCF1 gene expression ratio to macrophage differentiation**. a) Pyrosequencing was carried out to measure the ratio of NCF1B, NCF1 and NCF1C copies in the genomic DNA of THP-1 and differentiated macrophages. b) RT-qPCR was performed to measure the NCF1Ψ/NCF1 gene expression level using primers that recognize the GT or GTGT containing transcripts from all three gene copies. Five independent experiments were performed in duplicates using both GAPDH and β-actin as a reference. P values are indicated. c) Pyrosequencing was carried out to measure the relative contributions of NCF1 and its pseudogenes to the GTGT or GT containing transcript pool during macrophage differentiation.

Monocytes/Macrophages have been implicated in atherosclerosis [28]. After treatment with 800 ng/ml PMA for 12 hrs, the monocytes became adherent and acquired a macrophage-like phenotype. Both monocytes and macrophages have NCF1B:NCF1:NCF1C genomic copy number ratios of 2:2:2 (Figure 7a). After macrophage differentiation, quantitative RT-PCR experiments (RT-qPCR) were performed to measure the transcripts with the "GT-containing" exon 2 using primers that recognize all three NCF1 genes. The results showed that the NCF1/NCF1Ψs total GT-containing expression is slightly upregulated 1.34-fold (GAPDH, p = 0.024) to 1.82 fold (β-actin, p = 0.006) (Figure 7b). However, the relative contributions of each pseudogenes and the NCF1 gene copy to the GT-containing transcript pool were altered dramatically. NCF1B decreased from 32.9% to 8.3%, whereas NCF1 increased from 52.7% to 80.4% (Figure 7c).

### NCF1Ψ Expressions in Human Tissues

**Figure 8:**
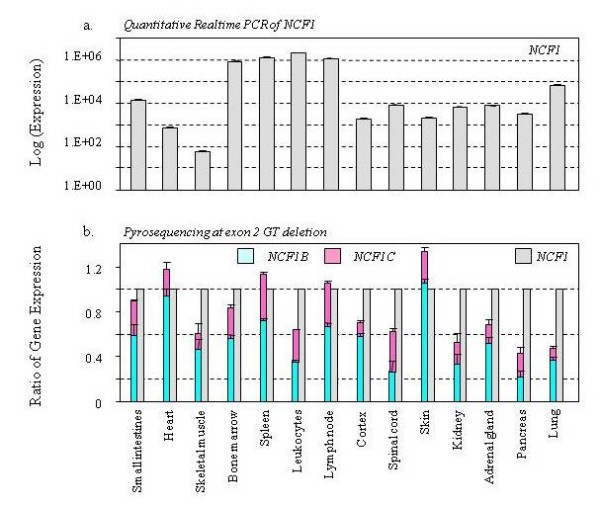
**NCF1Ψ/NCF1 gene expression in human tissues**. a). RT-qPCR was performed using primers specific to the NCF1 gene to determine the NCF1 expression in different human tissues. The data is in logarithmic scale. b). Pyrosequencing was performed to determine the relative transcript levels of NCF1, NCF1B and NCF1C. Three independent experiments were performed in duplicates.

We measured the NCF1B:NCF1:NCF1C transcript ratios by pyrosequencing the signature GT deletion in cDNAs generated from different human tissues. We observed that NCF1B and NCF1C expressions (GT-containing transcripts) varied dramatically in different human tissues (Figure 8). For example, skin produced the highest contribution of NCF1B relative to NCF1 expression, whereas pancreas produced the least amount of NCF1B. Spleen and lymph node produced the highest contribution of NCF1B, whereas lung and brain produced the least amount of NCF1C.

## Discussion

In this study, we report the existence of a NCF1 pseudogene CNV in human. The pseudogene copy numbers are apparently different among three human populations (African-Americans, Caucasians, and Mexicans). The CNV existence is validated by observance in genomic DNA extracted directly from human peripheral white blood cells. The NCF1 CNV inheritance found in the two family pedigrees, suggest that the chromosomal instability at this locus may not be high. Our phylogenetic analysis implies the existence of the NCF1 gene prior to the divergence of those two pseudogene duplicates, the NCF1 pseudogenes may emerge after the divergence of human and chimpanzee lineages. These data suggest that this NCF1 pseudogene CNV may be a consequence of recent gene duplications in human history [1].

Pseudogenes have long been considered to be 'dead' nonfunctional byproducts of genome evolution [29]. They were defined as genomic sequences that are similar to a functional gene but contain genetic defects that preclude the generation of functional products [30-32]. Recent findings have prompted to revise the definition of pseudogenes, which are now defined as genomic sequences that arise from functional genes but cannot encode the same type of functional products (i.e. protein, tRNA or rRNA) as the original genes [29]. Human genome is estimated to contain ~20,000 pseudogenes [33], it will be important to know how many and which pseudogenes are functional. Recently emphasis has been placed on polymorphisms such as CNV that has been documented to play a role in disease pathogenesis [34-39]. It has not been reported that a CNV of a pseudogene is biologically relevant. Historically, the NCF1 pseudogenes are considered "pseudo" because of their 2-bp GT deletion in exon 2, which is predicted to cause a frameshift and an early stop codon in protein synthesis. In our analysis of GT-containing transcript, the pseudogenes were far less active. However, our data revealed for the first time that a portion of NCF1 pseudogene transcripts do not utilize their defective exon-2, instead, they may use alternative exons to skip over their mutant exon-2 (Figure 6). When we measured transcript exclusive to exon-2, the NCF1 pseudogenes had varying transcriptional capacities that responded robustly to PMA induced macrophage differentiation (Figure 7) and showed distinct expression patterns in different human tissues (Figure 8). These observations prompted us to wonder if these NCF1 pseudogenes are "functional" or "unsuccessful duplicates". Recent studies have showed that ~95% of multi-exon genes undergo tissue-specific alternative splicing [40-42]; on the other hand, genes can also function by making regulatory non-coding RNAs in addition to making proteins [43]. These results certainly challenge the current perception of the NCF1 pseudogenes.

We observed differential expression of NCF1 and its pseudogenes and a varying contribution of the genes to the total transcript pool (Figure 5, 7, and 8). It may be caused by differential alternative splicing patterns, different promoter activities, and/or differential mRNA degradation. As indicated by sequence alignment with ClustalW2, the 5' untranslated regions of the NCF1 pseudogenes are nearly identical to the equivalent region of NCF1 (Additional file 7). Collectively, putative binding sites of 45 transcription factors (TFBS) were predicted with rVISTA (Additional file 7). The number and exact locations of these TFBS are also nearly identical among these three genes (Additional file 7), except that the NCF1 gene contains 19 AML1 TFBS and 4 CREB TFBS instead of the 18 AML1 sites and 3 CREB sites in both pseudogenes. Our data suggest that a portion of pseudogene transcripts did not adopt the GT-containing exon-2, which may explain our observation that NCF1 pseudogenes displayed fewer GT-containing transcripts relative to the true NCF1 gene. However, we are unclear if differential promoter activities and mRNA degradation contribute to this observation.

The CGD patients have shown that NCF1 is essential in the function of the neutrophil in the first line of host defense against many pathogenic bacteria and fungi. About 93% of humans patients caused by NCF1 mutations are homozygous for the 2-bp GT deletion [12,13]. Obviously the NCF1 pseudogenes, are not simply replacement duplicates, otherwise they may have compensated for the loss of NCF1 gene copies in the CGD patients. Two recent studies have reported that less copies of NCF1 pseudogenes may produce more reactive oxygen intermediates [44] and may exaggerate certain diseases involving inflammatory process such as inflammatory bowel disease [45]. Therefore, these two NCF1 pseudogenes may produce protein isoforms or small RNAs that act as inhibitors of the normal NCF1 function. Superoxide is a double-edged sword, it is essential for phagocytes to exert their bactericidal function, but in excess it is also toxic to our own cells. Cells in other tissues would benefit from the existence of these two pseudogenes if they could recognize different stimuli and then reduce the NCF1/NADPH oxidase activity and superoxide production accordingly. For example, they may help to reduce the inflammatory process during atherosclerosis.

## Conclusions

Taken together this study reported the existence of an NCF1 pseudogene CNV in three normal populations. These NCF1 pseudogenes are actively transcribed in human tissues. They can produce transcripts using alternative exon-2 instead of their defective exon-2. These results prompt us to re-consider their non-functional status in pseudogenes classification [29]. The functional contribution of the NCF1 pseudogenes to NCF1 function and the pathological relevance of this copy number variation will merit further investigations.

## Methods

### Subjects and Materials

Genomic DNAs of 86 unrelated individual DNA samples (The Human Variation Panels, 32 African-Americans [AA], 30 Caucasians [Cau], and 24 Mexicans [Mex]) and 2 CEPH/UTAH pedigrees (1331 and 1362) were obtained from The Coriell Cell Repositories. Single-donor Epstein-Barr virus (EBV)-immortalized lymphoblastoid cell lines (LCLs) were obtained from two sources: The Coriell Cell Repositories and The Emory Zafari Collection (kindly provided by Dr. Zafari of Emory Cardiology) [46]. Genomic DNAs of 48 unrelated healthy individuals directly extracted from peripheral blood cells instead of LCLs were kindly provided by Dr. Kittner of University of Maryland [47]. A human monocyte cell line THP-1 was purchased from ATCC (TIB-202). Total RNAs from human tissues were purchased from Clontech and US Biological. Phorbol 12-myristate 13-acetate (PMA) was purchased from Krackler Scientific (Cat. No. P1585). All subjects provided informed consent, and the study was approved by the Institutional Review Board of Morehouse School of Medicine.

### Cell Culture

Lymphoblastoid cells and THP-1 cells were grown in RMPI-1640 supplemented with 10% fetal bovine serum (FBS), 2 mM L-glutamine, and 1% penicillin/streptomycin. Cells were maintained in a humidified atmosphere containing 5% CO2 at 37°C. The differentiation of THP-1 monocytes into macrophages was induced by incubation in 800 ng/ml of PMA for 12 hours. The non-adherent cells were removed by aspiration; adherent cells were allowed to differentiate for 3-4 days.

### Genomic DNA and Total RNA Extraction

Genomic DNA was extracted from lymphoblastoid cells following a standard protocol described previously [48]. Total RNA was isolated using the RNeasy Mini kit (Qiagen). First strand cDNA was generated with 1 μg of total RNA using the Superscript III-RT system (Invitrogen).

### Genotyping

Genomic DNA and cDNA were subjected to genotyping at the 2-bp GT deletion in Exon-2 and a 1-bp difference in Exon-9 (Figure 1). PCR and pyrosequencing primers are provided in Additional file 8. These primers were designed to recognize both NCF1 and its pseudogenes without discrimination. The PCR products were examined by agarose gel electrophoresis to ensure PCR specificity. Genotyping was carried out with a pyrosequencer (PSQ96MA, Pyrosequencing, Uppsala, Sweden) following the manufacturer's protocol. Briefly, a 20 μL PCR reaction was carried out with either genomic DNA or cDNA. PCR products were then denatured and single-strand DNA templates were collected with streptavidin-coated Dynabeads (Dynal, Oslo, Norway). A pyrosequencing primer was added and pyrosequencing was performed in an automated PSQ96MA instrument. The pyrogram peak heights were evaluated per individual to give an accurate count of allele ratios using the PyroMarkID Software v1.0 provided by the manufacturer.

### Quantitative Real-Time PCR (RT-qPCR)

RT-qPCR was carried out using a LightCycler thermocycler (Roche) and a SYBR green kit (Roche). Oligo-dT primers were used in the reverse transcription, their sequences are provided in Additional file 8. Cycle numbers obtained at the log-linear phase of the reaction were plotted against a standard curve prepared with serially diluted control samples. Expressions of target genes were normalized by GAPDH and β-actin levels. The RT-qPCR amplification specificity of the NCF1-specific primer set is shown in Additional file 9.

### Statistical Analysis

All data is expressed as Mean ± Standard Deviation (SD). Student's *t *test was used in the setting of multiple comparisons where the appropriate and statistical significance is defined as *p *≤ 0.05.

### Bioinformatic analysis

Nucleotide sequences were retrieved from The UCSC Genome Browser (Human Assembly 2006 March). Sequence alignments were performed using multiple sequence alignment with MAP http://www.ebi.ac.uk/Tools/clustalw2/index.html. Phylogenetic trees were constructed using MegAlign ClustalW of DNAStar Software

## Authors' contributions

TB carried out all experiments, participated in study design and drafted the manuscript. QW assisted experiments. IC contributed to the monocyte experiments. QS designed the study, carried out the computational analysis in sequence alignment, ORF prediction and TFBS prediction. QS revised the manuscript. All authors have read and approved the final version of the manuscript.

## Supplementary Material

Additional file 1 **Genotyped positions of cDNA amplicons used in copy number variation analysis**. A) Amplicon generated at the exon-1 exon-2 boundary. B) Amplicon generated in exons 8 and 9. Genotyped locations are bold and underlined. Red base represents the end of an exon and blue represents the start of the neighboring exon. Click here for file

Additional file 2 **Representative pyrograms showing low pyrosequencing noise**. a) The 2-bp GT deletion in exon 2. b) The A/G substitution in exon 9. Click here for file

Additional file 3 **CNV allele frequencies in three populations**. Totally 86 non-related individuals (32 African-Americans, 30 Caucasians and 24 Mexicans) were genotyped at this CNV. Click here for file

Additional file 4 **Copy number variation at the NCF1 locus observed in genomic DNA samples extracted directly from peripheral white blood cells**. In order to eliminate the possibility that this CNV is an artefact caused by chromosomal instability in lymphoblastoid cell lines, we analyzed 48 genomic DNA samples directly extracted from human peripheral white blood cells. Means of 3 independent experiments performed in duplicate. Click here for file

Additional file 5 **cDNA sequences of alternatively spliced exons**. By PCR, cloning and direct DNA sequencing, we have experimentally discovered two novel alternative exons (GenBank: GU215077, GU215078) located in the intron-1. Neither of these two transcripts used the GT-containing exon-2. Click here for file

Additional file 6 **Putative open reading frames of alternative spliced transcripts**. The open reading frame (ORF) of two alternative spliced products, sub1 and sub2, were predicted with the NCBI ORF Finder. Click here for file

Additional file 7 **Putative transcription factor binding sites (TFBS) of the human NCF1 gene and its pseudogenes**. A 20 kb sequence of 5'-flanking region of each of NCF1 and its pseudogenes (immediately upstream to exon 1) was retrieved from UCSC Genome Browser. Sequence alignment was performed with EMBL-EBI CLUSTAL 2.0.12 (Larkin et al., 2007). Putative TFBS was predicted with rVISTA (Loots et al., 2002) using the vertebrates TRANSFAC matrices and cut-off 1.0 for both matrix similarity and core similarity. Click here for file

Additional file 8 **Primers used in this study**. This table provides the detailed sequence information of oligonucleotides that were used in this study. Click here for file

Additional file 9 **Melting curves of quantitative real-time PCR (RT-qPCR) using primers specific for the true p47^phox ^mRNA**. The specificity of this primer set is indicated the single sharp peak. The negative control is indicated by the straight line. Click here for file
