# Supplementary figures and images for: A copy number variation in human NCF1 and its pseudogenes
Source: BMC Genet. 2010 Feb 23;11:13. doi: 10.1186/1471-2156-11-13 (PMC2846862; doi:10.1186/1471-2156-11-13)

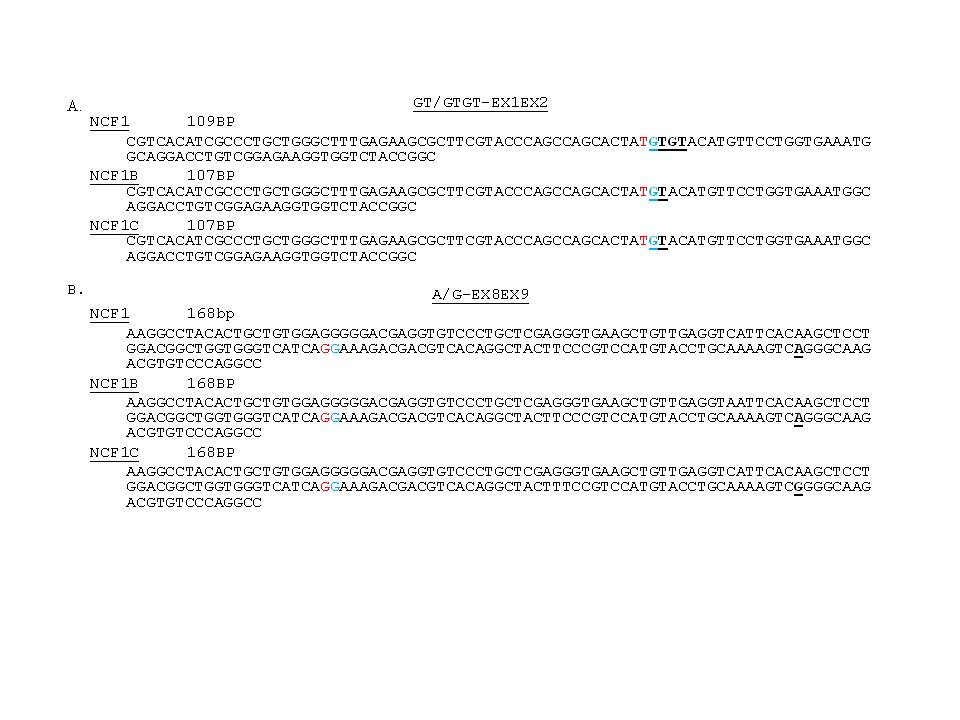

Supplement: Additional file 1 — Genotyped positions of cDNA amplicons used in copy number variation analysis. A) Amplicon generated at the exon-1 exon-2 boundary. B) Amplicon generated in exons 8 and 9. Genotyped locations are bold and underlined. Red base represents the end of an exon and blue represents the start of the neighboring exon. [file 1471-2156-11-13-S1.JPEG]

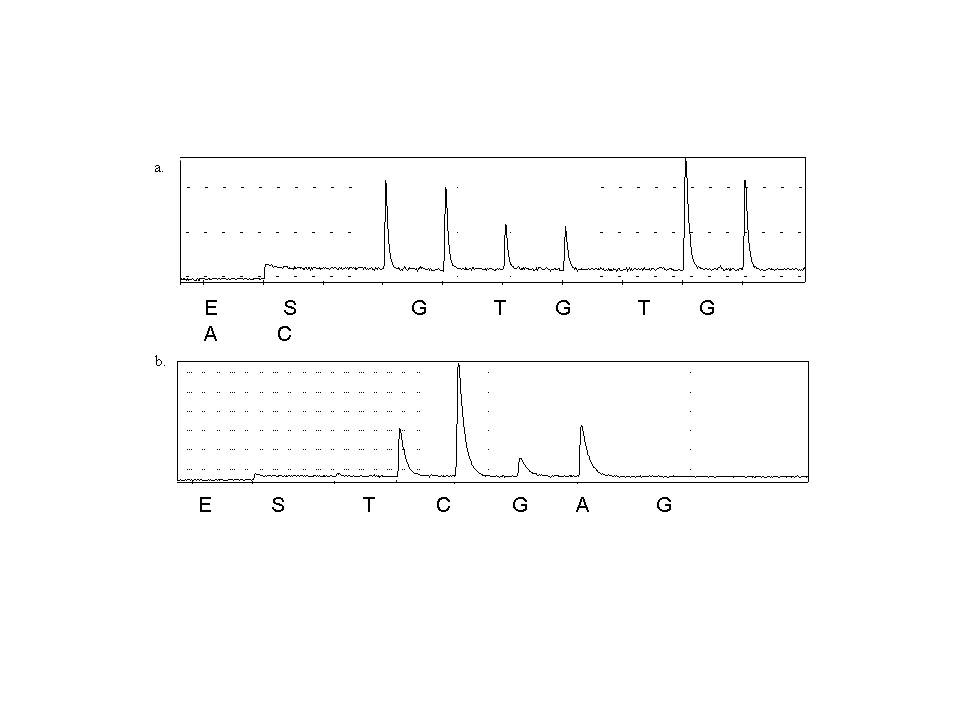

Supplement: Additional file 2 — Representative pyrograms showing low pyrosequencing noise. a) The 2-bp GT deletion in exon 2. b) The A/G substitution in exon 9. [file 1471-2156-11-13-S2.JPEG]

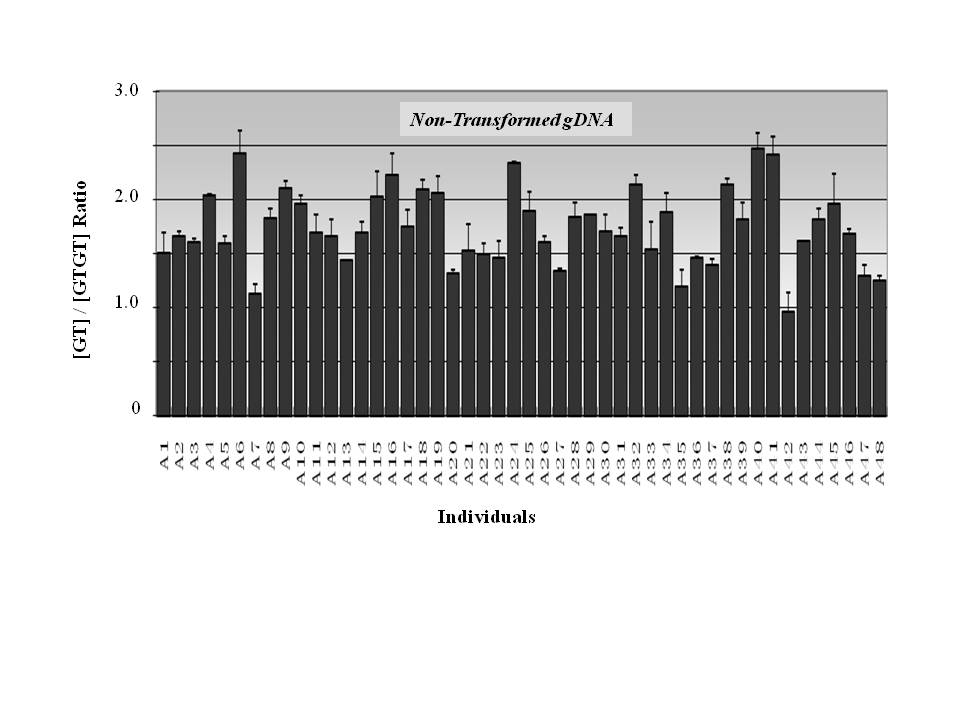

Supplement: Additional file 4 — Copy number variation at the NCF1 locus observed in genomic DNA samples extracted directly from peripheral white blood cells. In order to eliminate the possibility that this CNV is an artefact caused by chromosomal instability in lymphoblastoid cell lines, we analyzed 48 genomic DNA samples directly extracted from human peripheral white blood cells. Means of 3 independent experiments performed in duplicate. [file 1471-2156-11-13-S4.JPEG]

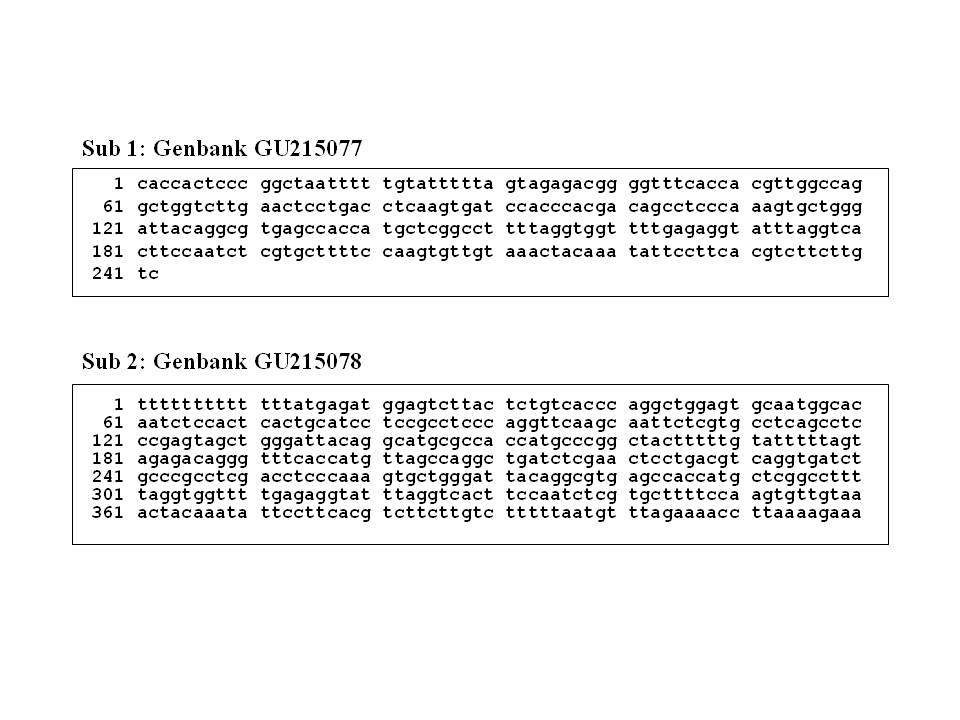

Supplement: Additional file 5 — cDNA sequences of alternatively spliced exons. By PCR, cloning and direct DNA sequencing, we have experimentally discovered two novel alternative exons (GenBank: GU215077, GU215078) located in the intron-1. Neither of these two transcripts used the GT-containing exon-2. [file 1471-2156-11-13-S5.JPEG]

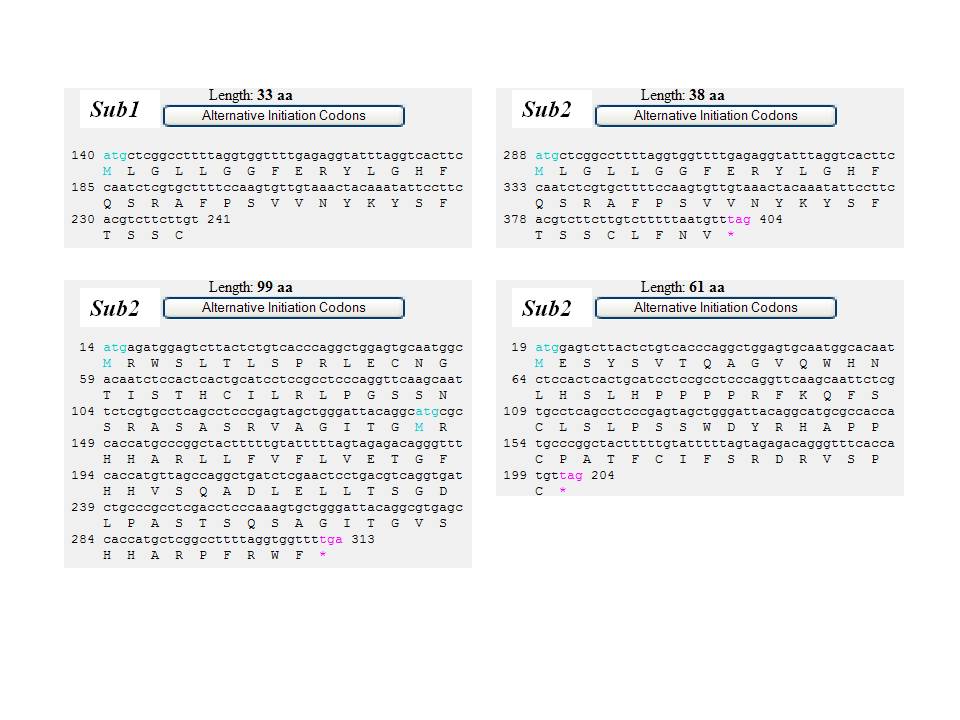

Supplement: Additional file 6 — Putative open reading frames of alternative spliced transcripts. The open reading frame (ORF) of two alternative spliced products, sub1 and sub2, were predicted with the NCBI ORF Finder. [file 1471-2156-11-13-S6.JPEG]

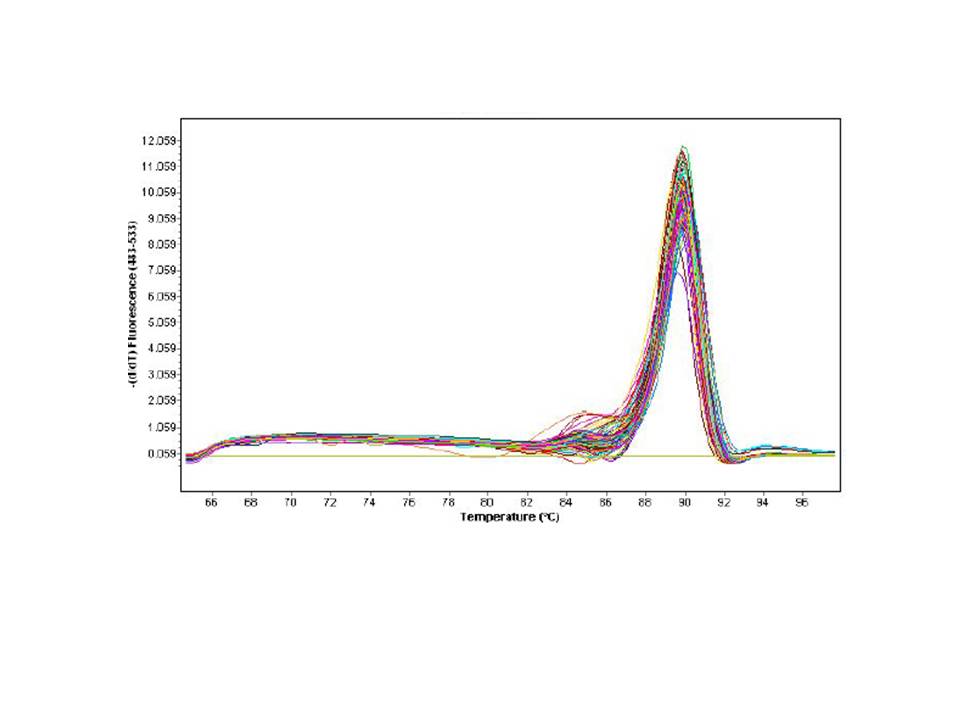

Supplement: Additional file 9 — Melting curves of quantitative real-time PCR (RT-qPCR) using primers specific for the true p47phox mRNA. The specificity of this primer set is indicated the single sharp peak. The negative control is indicated by the straight line. [file 1471-2156-11-13-S9.JPEG]
