# Supplementary material for: A copy number variation in human NCF1 and its pseudogenes
Source: BMC Genet. 2010 Feb 23;11:13. doi: 10.1186/1471-2156-11-13 (PMC2846862; doi:10.1186/1471-2156-11-13)
Supplement: Additional file 7 — Putative transcription factor binding sites (TFBS) of the human NCF1 gene and its pseudogenes. A 20 kb sequence of 5'-flanking region of each of NCF1 and its pseudogenes (immediately upstream to exon 1) was retrieved from UCSC Genome Browser. Sequence alignment was performed with EMBL-EBI CLUSTAL 2.0.12 (Larkin et al., 2007). Putative TFBS was predicted with rVISTA (Loots et al., 2002) using the vertebrates TRANSFAC matrices and cut-off 1.0 for both matrix similarity and core similarity. [file 1471-2156-11-13-S7.DOC]

**Additional file 7. Sequence alignment and putative transcription factor binding sites (TFBS) of the human NCF1 gene and its pseudogenes.** A 20 kb sequence of 5’-flanking region of each of NCF1 and its pseudogenes (immediately upstream to exon 1) was retrieved from UCSC Genome Browser. Sequence alignment was performed with EMBL-EBI CLUSTAL 2.0.12 (Larkin et al., 2007). Putative TFBS was predicted with rVISTA (Loots et al., 2002) using the vertebrates TRANSFAC matrices and cut-off 1.0 for both matrix similarity and core similarity.

NCF1B GAAATCAGCTCCATGAGAAGGATCTTAGACTCTGCCGAGTTTATCAAATTCACGGTCATT 60

NCF1C ----------CCATGAGAAGGATCTTAGACTCTGCCGAGTTTATCAAATTCACGGTCATT 50

NCF1 ---------TCCATGAGAAGGATCTTAGACTCTGCCGAGTTTATCAAATTCACGGTCATT 51

**************************************************

NCF1B AGGTAAGTGAGAGTTTCCTGCTTAGTCACAGGAGCGAATCTGGAGCTCATGAGGCTGACT 120

NCF1C AGGTAAGTGAGAGTTTCCTGCTTAGTCACAGGAGCGAATCTGGAGCTCATGAGGCTGACT 110

NCF1 AGGTAAGTGAGAGTTTCCTGCTTAGTCACAGGAGCGAATCTGGAGCTCATGAGGCTGACT 111

************************************************************

NCF1B CTTCTAAAATGCAGCCACAGGTAGTCATCGAATCCGGCTTCCTATGCTGTGCAATCAACA 180

NCF1C CTTCTAAAATGCAGCCACAGGTAGTCATCGAATCCGGCTTCCTATGCTGTGCAATCAACA 170

NCF1 CTTCTAAAATGCAGCCACAGGTAGTCATCGAATCCGGCTTCCTATGCTGTGCAATCAACA 171

************************************************************

NCF1B AATCAAAATAACTTGTGTCATCATTAGAATGTCAGATGTGCTTCTACGAACTAAGCTGAC 240

NCF1C AATCAAAATAACTTGTGTCATCATTAGAATGTCAGATGTGCTTCTACGAACTAAGCTGAC 230

NCF1 AATCAAAATAACTTGTGTCATCATTAGAATGTCAGATGTGCTTCTACGAACTAAGCTGAC 231

************************************************************

NCF1B TCTTTTAATTCTTTGGCAAAGGGTTGGCAAACTAGGACTGTTTGCCAAATTCAGGCTGCC 300

NCF1C TCTTTTAATTCTTTGGCAAAGGGTTGGCAAACTAGGACTGTTTGCCAAATTCAGGCTGCC 290

NCF1 TCTTTTAATTCTTTGGCAAAGGGTTGGCAAACTAGGACTGTTTGCCAAATTCAGGCTGCC 291 CEBP

************************************************************

NCF1B TCCTATTTTTAGAGTCTTCCTGAAACACAGCTACACCCGTATTATCCATGGCTGCTTTCC 360

NCF1C TCCTATTTTTAGAGTCTTCCTGAAACACAGCTACACCCGTATTATCCATGGCTGCTTTCC 350

NCF1 TCCTATTTTTAGAGTCTTCCTGAAACACAGCTACACCCGTATTATCCATGGCTGCTTTCC 351

************************************************************

NCF1B TGTCACAGTGAGTAAGTAGCTGGGACTGAGAAGGCATGGCCTCCAAAGTCTAAAATATTT 420

NCF1C TGTCACAGTGAGTAAGTAGCTGGGACTGAGAAGGCATGGCCTCCAAAGTCTAAAATATTT 410

NCF1 TGTCACAGTGAGTAAGTAGCTGGGACTGAGAAGGCATGGCCTCCAAAGTCTAAAATATTT 411

************************************************************

NCF1B ACTCTCTAGCACTTTGTAGAAAAACCTTAGCTAGGCACAGTGGCTAACGCCTGTAATCCC 480

NCF1C ACTCTCTAGCACTTTGTAGAAAAACCTTAGCTAGGCACAGTGGCTAACGCCTGTAATCCC 470

NCF1 ACTCTCTAGCACTTTGTAGAAAAACCTTAGCTAGGCACAGTGGCTAACGCCTGTAATCCC 471

************************************************************

NCF1B AGCATTTTGGGAGGCCAAGGCAGGCAGATCACCTGAGGTCAGGAGTTCCAGACCAGCCTG 540

NCF1C AGCATTTTGGGAGGCCAAGGCAGGCAGATCACCTGAGGTCAGGAGTTCCAGACCAGCCTG 530

NCF1 AGCATTTTGGGAGGCCAAGGCAGGCAGATCACCTGAGGTCAGGAGTTCCAGACCAGCCTG 531

************************************************************

NCF1B GCCAACATGGTGAAATCCCATCTCTACAAAAATAAAACAGTTAGCTGGGCATGATGGCGG 600

NCF1C GCCAACATGGTGAAATCCCATCTCTACAAAAATAAAACAGTTAGCTGGGCATGATGGCGG 590

NCF1 GCCAACATGGTGAAATCCCATCTCTACAAAAATAAAACAGTTAGCTGGGCATGATGGCGG 591

************************************************************

NCF1B GTGCCTGTAATCCCAGCTACTCGGGAGGCTGAGGCAGGATAGTCGCTTGAACCCAGGAGG 660

NCF1C GTGCCTGTAATCCCAGCTACTCGGGAGGCTGAGGCAGGATAGTCGCTTGAACCCAGGAGG 650

NCF1 GTGCCTGTAATCCCAGCTACTCGGGAGGCTGAGGCAGGATAGTCGCTTGAACCCAGGAGG 651

************************************************************

NCF1B TGGAGGTTGCAATGAGCCAAGATCATGCCACTGCACTCCAGCCTGGACGACAGAGTGAGA 720

NCF1C TGGAGGTTGCAATGAGCCAAGATCATGCCACTGCACTCCAGCCTGGACGACAGAGTGAGA 710

NCF1 TGGAGGTTGCAATGAGCCAAGATCATGCCACTGCACTCCAGCCTGGACGACAGAGTGAGA 711

************************************************************

NCF1B CTCCATCACAAGAAAAAAAGAACTTTGTCAACCTCTGTCTTAGGGCGCCTTGTCACAGGC 780

NCF1C CTCCATCACAAGAAAAAAAGAACTTTGTCAACCTCTGTCTTAGGGCGCCTTGTCACAGGC 770

NCF1 CTCCATCACAAGAAAAAAAGAACTTTGTCAACCTCTGTCTTAGGGCGCCTTGTCACAGGC 771

************************************************************

NCF1B TTCGGGTCAGACGGATTCAACCTCGCATCAGCCATTTGTTAGCCAGGTCACTCTGTTCTT 840

NCF1C TTCGGGTCAGACGGATTCAACCTCGCATCAGCCATTTGTTAGCCAGGTCACTCTGTTCTT 830

NCF1 TTCGGGTCAGACGGATTCAACCTTGCATCAGCCATTTGTTAGCCAGGTCACTCTGTTCTT 831 GR

*********************** ************************************

NCF1B TGTCTGTAAATGAGATTGATCGTTGTTCCCACTGAGAGTGTCAGCTCCTTCCCATAGAGC 900

NCF1C TGTCTGTAAATGAGATTGATCGTTGTTCCCACTGAGAGTGTCAGCTCCTTCCCATAGAGC 890

NCF1 TGTCTGTAAATGAGATTGATCGTTGTTCCCACTGAGAGTGTCAGCTCCTTCCCGTAGAGC 891

***************************************************** ******

NCF1B AGGCATGATGATTGTACTCACCTCTGACACCATTGTGAGTGCCACATTCCTTCCCACGTC 960

NCF1C AGGCATGATGATTGTACTCACCTCTGACACCATTGTGAGTGCCACATTCCTTCCCACGTC 950

NCF1 AGGCATGATGATTGTACTCACCTCTGACACCATTGTGAGTGCCACATTCCTTCCCACGTC 951 TEF1

************************************************************

NCF1B CTTGTCACCGTAAGAGATGCCCACCTGAGCACCAACCCCAGGTTATCTTCCCCTTTGTCT 1020

NCF1C CTTGTCACCGTAAGAGATGCCCACCTGAGCACCAACCCCAGGTTATCTTCCCCTTTGTCT 1010

NCF1 CTTGTCACTGTAAGAGATGCCCACCTGAGCACCAACCCCAGGTTATCTTCCCCTTTGTCT 1011

******** ***************************************************

NCF1B TCCAGCCCCCCAGAAACAGCTACGACTCAACCTACCCAATCATTTCATCATCAGATTGCC 1080

NCF1C TCCAGCCCCCCAGAAACAGCTACGACTCAACCTACCCAATCATTTCATCATCAGATTGCC 1070

NCF1 TCCAGCCCCCCAGAAACAGCTACGACTCAACCTACCCAATCATTTCATCATCAGATTGCC 1071 AREB6

************************************************************

NCF1B ACTGTCTCTAGTTCAGGTCTCTTGGGACTGGCACTCAGAAATCTCATAATAAATCCTCTT 1140

NCF1C ACTGTCTCTAGTTCAGGTCTCTTGGGACTGGCACTCAGAAATCTCATAATAAATCCTCTT 1130

NCF1 ACTGTCTCTAGTTCAGGTCTCTTGGGACTGGCACTCAGAAATCTCATAATAAATCCTCTT 1131 HNF4

************************************************************

NCF1B GAGGCTTCTCATACACTCGTCTTCTTCCAATCTTCTTTCCCTCAAAATCTCATATTTTGG 1200

NCF1C GAGGCTTCTCATACACTCGTCTTCTTCCAATCTTCTTTCCCTCAAAATCTCATATTTTGG 1190

NCF1 GAGGCTTCTCATACACTCGTCTTCTTCCAATCTTCTTTCCCTCAAAATCTCATATTTTGG 1191

************************************************************

NCF1B TTCCACTTCACCCACGGTCATTCTCCATATCACTCCCAGGAGTTAGGCAAAAAGCCCCTT 1260

NCF1C TTCCACTTCACCCACGGTCATTCTCCATATCACTCCCAGGAGTTAGGCAAAAAGCCCCTT 1250

NCF1 TTCCACTTCACCCACCGTCATTCTCCATATCACTCCCAGGAGTTAGGCAAAAAGCCCCTT 1251 CREB (NCF1-specific)

*************** ********************************************

NCF1B CCGTTCTTCCGTATGTTAAACTTAGAATCACTCTGTTCCCTGCTCTGCGTTTCTATTTTT 1320

NCF1C CCGTTCTTCCGTATGTTAAACTTAGAATCACTCTGTTCCCTGCTCTGCGTTTCTATTTTT 1310

NCF1 CCGTTCTTCCGTATGTTAAACTTAGAATCACTCTGTTCCCTGCTCTGCGTTTCTATTTTT 1311 SRY

************************************************************

NCF1B TGTTTTTCCTCCATTTACTAGTAGCTTAACACTTTCTAACAGTGTTCTTATTATTGATAC 1380

NCF1C TGTTTTTCCTCCATTTACTAGTAGCTTAACACTTTCTAACAGTGTTCTTATTATTGATAC 1370

NCF1 TGTTTTTCCTCCATTTACTAGTAGCTTAACACTTTCTAACAGTGTTCTTATTATTGATAC 1371

************************************************************

NCF1B GTATCTATCTCTTCCATAAGCTTATAAGGTCATGGATAATACTTCTCATTGTAGTACGTA 1440

NCF1C GTATCTATCTCTTCCATAAGCTTATAAGGTCATGGATAATACTTCTCATTGTAGTACGTA 1430

NCF1 GTATCTATCTCTTCCATAAGCTTATAAGGTCACGGATAATACTTCTCATTGTAGTACGTA 1431

******************************** ***************************

NCF1B AATGACGTGGGCTAGATATGAGTTGAATAAACAGTTATACCTGTAAATTCTTACAGAGTG 1500

NCF1C AATGACGTGGGCTAGATATGAGTTGAATAAACAGTTATACCTGTAAATTCTTACAGAGTG 1490

NCF1 AATGACGTGGGCTAGATATGAGTTGAATAAACAGTTATACCTGTAAATTCTTACAGAGTG 1491 CREB**,** ATF6

************************************************************

NCF1B AAAATAAATTGTTATACTTTACAATTTGTTTCTCTCTTTAGACCATTTCCAGGACTTGTG 1560

NCF1C AAAATAAATTGTTATACTTTACAATTTGTTTCTCTCTTTAGACCATTTCCAGGACTTGTG 1550

NCF1 AAAATAAATTGTTATACTTTACAATTTGTTTCTCTCTTTAGACCATTTCCAGGACTTGTG 1551 SRY

************************************************************

NCF1B ATTAATAACCGTGAGTATTTTGTGAAGTGTTTTGTTTTTGTTTTTTCCTGGGGTCTGACG 1620

NCF1C ATTAATAACCGTGAGTATTTTGTGAAGTGTTTTGTTTTTGTTTTTTCCTGGGGTCTGACG 1610

NCF1 ATTAATAACCGTGAGTATTTTGTGAAGTGTTTTGTTTTTGTTTTTTCCTGGGGTCTGACG 1611 SRY

************************************************************

NCF1B TGTGTGCGTGTGAGTGTGTATACATGCTTAACGTATATCACGTTACTTCACCTATGTCAG 1680

NCF1C TGTGTGCGTGTGAGTGTGTATACATGCTTAACGTATATCACGTTACTTCACCTATGTCAG 1670

NCF1 TGTGTGCGTGTGAGTGTGTATACATGCTTAACGTATATCACGTTACTTCACCTATGTCAG 1671

************************************************************

NCF1B TAACCAGGCCAAATACTTGTTTTAGCCCTCAGTAAAAACACCAGGCACTTCCTAGTTGTA 1720 PEA3

NCF1C TAACCAGGCCAAATACTTGTTTTAGCCCTCAGTAAAAACACCAGGCACTTCCTAGTTGTA 1730

NCF1 TAACCAGGCCAAATACTTGTTTTAGCCCTCAGTAAAAACACCAGGCACTTCCTAGTTGTA 1731

************************************************************

NCF1B AAATTATTCAAGCTTCTTAACTTCCTATCCTCGATGCACTTAATCATAAAATGGTAATAA 1800

NCF1C AAATTATTCAAGCTTCTTAACTTCCTATCCTCGATGCACTTAATCATAAAATGGTAATAA 1790

NCF1 AAATTATTCAAGCTTCTTAACTTCCTATCCTCGATGCACTTAATCATAAAATGGTAATAA 1791 PEA3

************************************************************

NCF1B TAGCACCGATTTTGGGGGAGTCGTTCCAGTAGATGGAAAGCATCTGGAACAGGTGTCAGC 1860

NCF1C TAGCACCGATTTTGGGGGAGTCGTTCCAGTAGATGGAAAGCATCTGGAACAGGTGTCAGC 1850

NCF1 TAGCACCGATTTTGGGGGAGTCGTTCCAGTAGATGGAAAGCATCTGGAACAGGTGTCAGC 1851

************************************************************

NCF1B AAGCTCCTGCCGACGTGTATGCATAAAGTTTTATTGAAACCACCATCGTGTCCATTTGTT 1920

NCF1C AAGCTCCTGCCGACGTGTATGCATAAAGTTTTATTGAAACCACCATCGTGTCCATTTGTT 1910

NCF1 AAGCTCCTGCCGACGTGTATGCATAAAGTTTTATTGAAACCACCATCGTGTCCATTTGTT 1911 SRY, FoxO1

************************************************************

NCF1B TATGGCAGAACTGAGAGATGGCAGCAGCAAGTGTGTGCCTGCAAAACCTAAAATATTTAC 1980

NCF1C TATGGCAGAACTGAGAGATGGCAGCAGCAAGTGTGTGCCTGCAAAACCTAAAATATTTAC 1970

NCF1 TATGGCAGAACTGAGAGATGGCAGCAGCAAGTGTGTGCCTGCAAAACCTAAAATATTTAC 1971

************************************************************

NCF1B TAATTGGCTCTGCAAGAAAAGAGTTTGCATCCCCCTAACCTAGAACAGTGTCTAGCCTCT 2040

NCF1C TAATTGGCTCTGCAAGAAAAGAGTTTGCATCCCCCTAACCTAGAACAGTGTCTAGCCTCT 2030

NCF1 TAATTGGCTCTGCAAGAAAAGAGTTTGCATCCCCCTAACCTAGAACAGTGTCTAGCCTCT 2031

************************************************************

NCF1B AGTATGTGTTTAGCCTACAGTATGTGCTCAGCGAATACAATCTATATTTATTACTGCTTT 2100

NCF1C AGTATGTGTTTAGCCTACAGTATGTGCTCAGCGAATACAATCTATATTTATTACTGCTTT 2090

NCF1 AGTATGTGTTTAGCCTACAGTATGTGCTCAGCGAATACAATCTATATTTATTACTGCTTT 2091

************************************************************

NCF1B TATGACTGTTATAATTACTGTGCTTGGATTTCGTTACAAAGTAAGTCACAATGTGCCTGC 2160

NCF1C TATGACTGTTAGAATTACTGTGCTTGGATTTCGTTACAAAGTAAGTCACAATGTGCCTGC 2150

NCF1 TATGACTGTTATAATTACTGTGCTTGGATTTCGTTACAAAGTAAGTCACAATGTGCCTGC 2151

*********** ************************************************

NCF1B TTCTGTTAGTATTTCAGCACAGTGCCTGGCACACATGGGGCTCTCAAATATTGCTGAGCG 2220

NCF1C TTCTGTTAGTATTTCAGCACAGTGCCTGGCACACATGGGGCTCTCAAATATTGCTGAGCG 2210

NCF1 TTCTGTTAGTATTTCAGCACAGTGCCTGGCACACATGGGGCTCTCAAATATTGCTGAGCG 2211

************************************************************

NCF1B AGTGAACAAATGTCCTTTCAATTCCTTAACGTTGATGTCATTTTCAATAGTATTTTGAGC 2280

NCF1C AGTGAACAAATGTCCTTTCAATTCCTTAACGTTGATGTCATTTTCAATAGTATTTTGAGC 2270

NCF1 AGTGAACAAATGTCCTTTCAATTCCTTAACGTTGATGTCATTTTCAATAGTATTTTGAGC 2271

************************************************************

NCF1B CAAACTTAATTTTGCGAGTGTGTTTTGTTTTCTTAACTTTATTATTAAAAATGTATAAAA 2340

NCF1C CAAACTTAATTTTGCGAGTGTGTTTTGTTTTCTTAACTTTATTATTAAAAATGTATAAAA 2330

NCF1 CAAACTTAATTTTGCGAGTGTGTTTTGTTTTCTTAACTTTATTATTAAAAATGTATAAAA 2331 SRY

************************************************************

NCF1B GTGAGGCCAGGCGTGGTGGCTCACGCCTGTAATCCCAGCAGTTTGGGAGGCTGAGGCAGG 2400

NCF1C GTGAGGCCAGGCGTGGTGGCTCACGCCTGTAATCCCAGCAGTTTGGGAGGCTGAGGCAGG 2390

NCF1 GTGAGGCCAGGCGTGGTGGCTCACGCCTGTAATCCCAGCAGTTTGGGAGGCTGAGGCAGG 2391

************************************************************

NCF1B CGGATCAGTTGAGGTTGGGAGTTGGAGACCAGCCCGTCCAACATGGTGAAAGCCTGTCTC 2460

NCF1C CGGATCAGTTGAGGTTGGGAGTTGGAGACCAGCCCGTCCAACATGGTGAAAGCCTGTCTC 2450

NCF1 CGGATCAGTTGAGGTTGGGAGTTGGAGACCAGCCCGTCCAACATGGTGAAAGCCTGTCTC 2451

************************************************************

NCF1B TACTAAAAATACAAAAATCAGCTGGGTGTGGTGGCGCGTGCCTGTAATCCCAGCTACTCA 2520

NCF1C TACTAAAAATACAAAAATCAGCTGGGTGTGGTGGCGCGTGCCTGTAATCCCAGCTACTCA 2510

NCF1 TACTAAAAATACAAAAATCAGCTGGGTGTGGTGGCGCGTGCCTGTAATCCCAGCTACTCA 2511 AML1, E2F

************************************************************

NCF1B GGAGGCTGAGGCAGGAGAGTTGCTTGAATCCTAGAGGTGGAGGTTGCAGTGAGCTGAGAT 2580

NCF1C GGAGGCTGAGGCAGGAGAGTTGCTTGAATCCTAGAGGTGGAGGTTGCAGTGAGCTGAGAT 2570

NCF1 GGAGGCTGAGGCAGGAGAGTTGCTTGAATCCTAGAGGTGGAGGTTGCAGTGAGCTGAGAT 2571

************************************************************

NCF1B CGTGCCATTGCACTCCAGCCTGGGCAACAAGAGCAAAACTCTGTCCCAATAAATAAATAA 2640

NCF1C CGTGCCATTGCACTCCAGCCTGGGCAACAAGAGCAAAACTCTGTCCCAATAAATAAATAA 2630

NCF1 CGTGCCATTGCACTCCAGCCTGGGCAACAAGAGCAAAACTCTGTCCCAATAAATAAATAA 2631

************************************************************

NCF1B ATAAAATGTTCTCTTTTGTTCCTGTTCTTGTGCGGTAGTGTGGTATAGAGTTTTATGGTA 2700

NCF1C ATAAAATGTTCTCTTTTGTTCCTGTTCTTGTGCGGTAGTGTGGTATAGAGTTTTATGGTA 2690

NCF1 ATAAAATGTTCTCTTTTGTTCCTGTTCTTGTGCGGTAGTGTGGTATAGAGTTTTATGGTA 2691 AML1

************************************************************

NCF1B ATTACTGTGAATTAGTGATTCTGAGGGACATATCAGAACTCTGAGGTTTGTTTCCTTCTC 2760

NCF1C ATTACTGTGAATTAGTGATTCTGAGGGACATATCAGAACTCTGAGGTTTGTTTCCTTCTC 2750

NCF1 ATTACTGTGAATTAGTGATTCTGAGGGACATATCAGAACTCTGAGGTTTGTTTCCTTCTC 2751 SRY

************************************************************

NCF1B ATCTTGAGGGAAACAGCAAATGTATGTTAAAATGCTTTTCCAAGGGAACAACACATCCTT 2820

NCF1C ATCTTGAGGGAAACAGCAAATGTATGTTAAAATGCTTTTCCAAGGGAACAACACATCCTT 2810

NCF1 ATCTTGAGGGAAACAGCAAATGTATGTTAAAATGCTTTTCCAAGGGAACAACACATCCTT 2811 PEA3

************************************************************

NCF1B ACATTATTTAAACCAATCTGCTTCATTTTCAGAGCTGGTTGATCAGAGTGAGTCAAAAGG 2880

NCF1C ACATTATTTAAACCAATCTGCTTCATTTTCAGAGCTGGTTGATCAGAGTGAGTCAGAAGG 2870

NCF1 ACATTATTTAAACCAATCTGCTTCATTTTCAGAGCTGGTTGATCAGAGTGAGTCAGAAGG 2871

******************************************************* ****

NCF1B CCCCGTGATACAAGGTGAGCGAGGCAGGGGAGGGCCCGGAGCTACTCCTGCCTGCACAGT 2940

NCF1C CCCCGTGATACAAGGTGAGCGAGGCAGGGGAGGGCCCGGAGCTACTCCTGCCTGCACAGT 2930

NCF1 CCCCGTGATACAAGGTGAGCGAGGCAGGGGAGGGCCCGGAGCTACTCCTGCCTGCACAGT 2931 MAZ

************************************************************

NCF1B GGCACACATGGCGTGCCTGCGTGTGGCTTTGGCTCTCAGTCACCTGCCCTGAGGGGACTC 3000

NCF1C GGCACACATGGCGTGCCTGCGTGTGGCTTTGGCTCTCAGTCACCTGCCCTGAGGGGACTC 2990

NCF1 GGCACACATGGCGTGCCTGCGTGTGGCTTCGGCTCTCAGTCACCTGCCCTGAGGGGACTC 2991 E2A (myogenin)

***************************** ******************************

NCF1B AGTTACACAGCACACACATGCTTCTCTGTGGTTTTCACTCCTGGGTTTGACAGCTGATCA 3060

NCF1C AGTTACAC--TACACACATGCTTCTCTGTGGTTTTCACTCCTGGGTTTGACAGCTGATCA 3048

NCF1 AGTTACACAGCACACACATGCTTCTCTGTGGTTTTCACTCCTGGGTTTGACAGCTGATCA 3051 AML1

******** *************************************************

NCF1B AAACATAAATTCAAGCTGTGGGTCCTGATTGAGAACTGGGGGCTGCAGACCATTTGCACC 3120

NCF1C AAACATAAATTCAAGCTGTGGGTCCTGATTGAGAACTGGGGGCTGCAGACCATTTGCACC 3108

NCF1 AAACATAAATTCAAGCTGTGGGTCCTGATTGAGAACTGGGGGCTGCAGACCATTTGCACC 3111

************************************************************

NCF1B CCCTATCCCAGCTCAGGCCTAACATCAGGAACCCCAGGATTAATGGGTAGGATGAAATGG 3180

NCF1C CCCTATCCCAGCTCAGGCCTAACATCAGGAACCCCAGGATTAATGGGTAGGATGAAATGG 3168

NCF1 CCCTATCCCAGCTCAGGCCTAACATCAGGAACCCCAGGATTAATGGGTAGGATGAAATGG 3171

************************************************************

NCF1B CAGAGCAAGAGGGCCGTCACTTTAACCTGACTCTGCCATCCATTTCTAATGTCTGCCATA 3240

NCF1C CAGAGCAAGAGGGCCGTCACTTTAACCTGACTCTGCCATCCATTTCTAATGTCTGCCATA 3228

NCF1 CAGAGCAAGAGGGCCGTCACTTTAACCTGACTCTGCCATCCATTTCTAATGTCTGCCATA 3231 Stat5A

************************************************************

NCF1B AGTCAGTGAGCAAAATGTTCTTCAGTAGAAATGTACAGATTGTGCTCTTAAAAAATTCCT 3300

NCF1C AGTCAGTGAGCAAAATGTTCTTCAGTAGAAATGTACAGATTGTGCTCTTAAAAAATTCCT 3288

NCF1 AGTCAGTGAGCAAAATGTTCTTCAGTAGAAATGTACAGATTGTGCTCTTAAAAAATTCCT 3291 Stat5A

************************************************************

NCF1B TAAAAAACAAGTGGAATGGCCTGGTGCTGTGTGAGTCATTGAAAGTAATGAGACTGGGCG 3360

NCF1C TAAAAAACAAGTGGAATGGCCTGGTGCTGTGTGAGTCATTGAAAGTAATGAGACTGGGCG 3348

NCF1 TAAAAAACAAGTGGAATGGCCTGGTGCTGTGTGAGTCATTGAAAGTAATGAGACTGGGCG 3351 TEF1, AP1

************************************************************

NCF1B CGGTGGCTCACGCCTGTTATCCCAGCACTTTGGAAGGCTGAGAAGGGTAGATCACTTGAG 3420 Nkx25

NCF1C CGGTGGCTCACGCCTGTTATCCCAGCACTTTGGAAGGCTGAGAAGGGTAGATCACTTGAG 3408

NCF1 TGGTGGCTCACGCCTGTTATCCCAGCACTTTGGAAGGCTGAGAAGGGTAGATCACTTGAG 3411

***********************************************************

NCF1B ATCAGGAGTTCGAGACCAGCCTGGCCAACATGGTGCAACCCCGTCTCTACTAAGAATACA 3480

NCF1C ATCAGGAGTTCGAGACCAGCCTGGCCAACATGGTGCAACCCCGTCTCTACTAAGAATACA 3468

NCF1 ATCAGGAGTTCGAGACCAGCCTGGCCAACATGGTGCAACCCCGTCTCTACTAAGAATACA 3471

************************************************************

NCF1B AAAACTAGCCAGACGTGGTGGCGTGTGCCGGCTACTCAGGAGGCTGAGGCAGGAGAACCG 3540

NCF1C AAAACTAGCCAGACGTGGTGGCGTGTGCCGGCTACTCAGGAGGCTGAGGCAGGAGAACCG 3528

NCF1 AAAACTAGCCAGACGTGGTGGCGTGTGCCGGCTACTCAGGAGGCTGAGGCAGGAGAACCG 3531

************************************************************

NCF1B CTTGAGCCTGGGAGGCGGAGGTCGCAGTGAGCCAAGATCGTGCCACTGCACTCCAGCCTG 3600

NCF1C CTTGAGCCTGGGAGGCGGAGGTCGCAGTGAGCCAAGATCGTGCCACTGCACTCCAGCCTG 3588

NCF1 CTTGAGCCTGGGAGGCGGAGGTCGCAGTGAGCCAAGATCGTGCCACTGCACTCCAGCCTG 3591

************************************************************

NCF1B GGCAACAGAAAGGAGATTCTGTCTCAAAAAAACAAACATACGAAGAAAAACAAAAAAAGT 3660

NCF1C GGCAACAGAAAGGAGATTCTGTCTCAAAAAAACAAACATACGAAGAAAAACAAAAAAAGT 3648

NCF1 GGCAACAGAAAGGAGATTCTGTCTCAAAAAAACAAACATACGAAGAAAAACAAAAAAAGT 3651 HNF3a, SRY, SRY

************************************************************

NCF1B AATGAAAAGCTTTTATTAAAGGGAGTAAACAGAAGGATAAGGGAGAAAGCATAACTAAGG 3720

NCF1C AATGAAAAGCTTTTATTAAAGGGAGTAAACAGAAGGATAAGGGAGAAAGCATAACTAAGG 3708

NCF1 AATGAAAAGCTTTTATTAAAGGGAGTAAACAGAAGGATAAGGGAGAAAGCATAACTAAGG 3711

************************************************************

NCF1B AGCTTGTTTTCATGGTAGAGCTATGTTAAGACTCTGCTCTTTCAAACTTCAGTTGTATAT 3780

NCF1C AGCTTGTTTTCATGGTAGAGCTATGTTAAGACTCTGCTCTTTCAAACTTCAGTTGTATAT 3768

NCF1 AGCTTGTTTTCATGGTAGAGCTATGTTAAGACTCTGCTCTTTCAAACTTCAGTTGTATAT 3771

************************************************************

NCF1B GTGAACTTAGGACCACATTTGAAAAACAGAAATTTGAAAGTACACTTGGATAATCGTGTG 3840

NCF1C GTGAACTTAGGACCACATTTGAAAAACAGAAATTTGAAAGTACACTTGGATAATCGTGTG 3828

NCF1 GTGAACTTAGGACCACATTTGAAAAACAGAAATTTGAAAGTACACTTGGATAATCGTGTG 3831 HNF4, AML1

************************************************************

NCF1B CTCCATCTCAAGACCGTGAGCATTGTTTCATCATGCACCTGTGTTTGTACAGAGTCTAGA 3900

NCF1C CTCCATCTCAAGACCGTGAGCATTGTTTCATCATGCACCTGTGTTTGTACAGAGTCTAGA 3888

NCF1 CTCCATCTCAAGACCGTGAGCATTGTTTCATCATGCACCTGTGTTTGTACAGAGTCTAGA 3891

************************************************************

NCF1B GGGCTTTTCTCCTCTTCCTCCTCCTGGGTTCTTTACATAGTATAAAGCAGCTGTTGAACA 3960

NCF1C GGGCTTTTCTCCTCTTCCTCCTCCTGGGTTCTTTACATAGTATAAAGCAGCTGTTGAACA 3948

NCF1 GGGCTTTTCTCCTCTTCCTCCTCCTGGGTTCTTTACATAGTATAAAGCAGCTGTTGAACA 3951 LBP1

************************************************************

NCF1B ATGTGGAAATCAGTCTCTGTGTTTCTCTTTAGAATCAGCTGAACCAAGCCAGTTGGAAGT 4020

NCF1C ATGTGGAAATCAGTCTCTGTGTTTCTCTTTAGAATCAGCTGAACCAAGCCAGTTGGAAGT 4008

NCF1 ATGTGGAAATCAGTCTCTGTGTTTCTCTTTAGAATCAGCTGAACCAAGCCAGTTGGAAGT 4011

************************************************************

NCF1B TCCAGCCACAGAAGGTAAAAGGGTGGGGTGGTCCTGCAAGTCCTTAAGACTTCTTCTTTC 4080

NCF1C TCCAGCCACAGAAGGTAAAAGGGTGGGGTGGTCCTGCAAGTCCTTAAGACTTCTTCTTTC 4068

NCF1 TCCAGCCACAGAAGGTAAAAGGGTGGGGTGGTCCTGCAAGTCCTTAAGACTTCTTCTTTC 4071 SREBP1

************************************************************

NCF1B TTCTTCTTCTTTTTTTTTTTTTTAAAGACAGAGACTTGCTCTGTCACCCAGGGTGGAGTG 4140

NCF1C TTCTTCTTCTTTTTTTTTTTTTTAAAGACAGAGACTTGCTCTGTCACCCAGGGTGGAGTG 4128

NCF1 TTCTTC---TTTTTTTTTTTTTTAAAGACAGAGACTTGCTCTGTCACCCAGGGTGGAGTG 4128

****** ***************************************************

NCF1B AGGTTGCGCGATCTCTGCAACCTCCGCCTCCCGGGCTCAAGCAGTTCTCCTGCCTCAGCC 4200

NCF1C AGGTTGCGCGATCTCTGCAACCTCCGCCTCCCGGGCTCAAGCAGTTCTCCTGCCTCAGCC 4188

NCF1 AGGTTGCGCGATCTCTGCAACCTCCGCCTCCCGGGCTCAAGCAGTTCTCCTGCCTCAGCC 4188

************************************************************

NCF1B TCCCGAGTAGCTGGGATTACAGGCCTGCACCACCATGCTTGGCTAATTTTTGTATTTTTA 4260

NCF1C TCCCGAGTAGCTGGGATTACAGGCCTGCACCACCATGCTTGGCTAATTTTTGTATTTTTA 4248

NCF1 TCCCGAGTAGCTGGGATTACAGGCCTGCACCACCATGCTTGGCTAATTTTTGTATTTTTA 4248

************************************************************

NCF1B GTAGAAACGGGGTTTCACCATGTTGGCCAGGCTAGTCTCAGACTTCTGACCTCAAGTGAT 4320

NCF1C GTAGAAACGGGGTTTCACCATGTTGGCCAGGCTAGTCTCAGACTTCTGACCTCAAGTGAT 4308

NCF1 GTAGAAACGGGGTTTCACCATGTTGGCCAGGCTAGTCTCAGACTTCTGACCTCAAGTGAT 4308 Nkx25

************************************************************

NCF1B CTGCCAGCCTTGGCCTCCAAAGTGCTCGGATTACAGGCGCGAGCCACCTTGCCCAGCCAA 4380

NCF1C CCGCCAGCCTTGGCCTCCAAAGTGCTCGGATTACAGGCGCGAGCCACCTTGCCCAGCCAA 4368

NCF1 CCGCCAGCCTTGGCCTCCAAAGTGCTCGGATTACAGGCGCGAGCCACCTTGCCCAGCCAA 4368 E2F

* **********************************************************

NCF1B GACTTTTTTATCAGGACAAAGGATTGTGCATTTAAACTATTTCACTAGAACTGGGTGGTG 4440

NCF1C GACTTTTTTATCAGGACAAAGGATTGTGCATTTAAACTATTTCACTAGAACTGGGTGGTG 4428

NCF1 GACTTTTTTATCAGGACAAAGGATTGTGCATTTAAACTATTTCACTAGAACTGGGTGGTG 4428

************************************************************

NCF1B GTTTTGCTCTCTTTCTTCTGGGTGAATTGGATTTGCAGGTTATGCTGTTGAGTGATGACG 4500

NCF1C GTTTTGCTCTCTTTCTTCTGGGTGAATTGGATTTGCAGGTTATGCTGTTGAGTGATGACG 4488

NCF1 GTTTTGCTCTCTTTCTTCTGGGTGAATTGGATTTGCAGGTTATGCTGTTGAGTGATGACG 4488 CREB

************************************************************

NCF1B CATAGCTGCTTTTGCTCCATTTTCCCCAGATGACTTGGTAAATTCTCCGTGAATGACTCT 4560

NCF1C CATAGCTGCTTTTGCTCCATTTTCCCCAGATGACTTGGTAAATTCTCCGTGAATGACTCT 4548

NCF1 CATAGCTGCTTTTGCTCCATTTCCCCCAGATGACTTGGTAAATTCTCCGTGAATGACTCT 4548

********************** *************************************

NCF1B GCTACATAACCTAGATAACCTAA-GTGTGTCCTTTAAATGCATGTAAGCCAGAAGATGTA 4619

NCF1C GCTACATAACCTAGATAACCTAA-GTGTGTCCTTTAAATGCATGTAAGCCAGAAGATGTA 4607

NCF1 GCTACATAACCTAGATAACCTAACGTGTGTCCTTTAAATGCATGTAAGCCAGAAGATGTA 4608

*********************** ************************************

NCF1B TGTTACTTTGAAAACATAAGTAACAAAATTTTGAATGTATTGCTAAAGAGATGTCTCTCT 4679 LEF1

NCF1C TGTTACTTTGAAAACATAAGTAACAAAATTTTGAATGTATTGCTAAAGAGATGTCTCTCT 4667

NCF1 TGTTACTTTGAAAACATAAGTAACAAAATTTTGAATGTATTGCTAAAGAGATGTCTCTCT 4668

************************************************************

NCF1B GAAGCTCTTTTGATGTTTGGTGTCTTGTCCTTCTTATTAAACCATATCTTAGTAAATAGT 4739

NCF1C GAAGCTCTTTTGATGTTTGGTGTCTTGTCCTTCTTATTAAACCATATCTTAGTAAATAGT 4727

NCF1 GAAGCTCTTTTGATGTTTGGTGTCTTGTCCTTCTTATTAAACCATATCTTAGTAAATAGT 4728

************************************************************

NCF1B TTGGTACGAATGGATTTATCACTGAGCAGGTCTGCAAAATAATTAATCGGTACCGTTTTG 4799

NCF1C TTGGTACGAATGGATTTATCACTGAGCAGGTCTGCAAAATAATTAATCGGTACCGTTTTG 4787

NCF1 TTGGTACGAATGGATTTATCACTGAGCAGGTCTGCAAAATAATTAATCGGTACCGTTTTG 4788 SRY

************************************************************

NCF1B TTTCTGTTGATAGAAATAAAAGAGACTGATGGAAGCTCTCAGATCAAGCAAGAACCAGAC 4859

NCF1C TTTCTGTTGATAGAAATAAAAGAGACTGATGGAAGCTCTCAGATCAAGCAAGAACCAGAC 4847

NCF1 TTTCTGTTGATAGAAATAAAAGAGACTGATGGAAGCTCTCAGATCAAGCAAGAACCAGAC 4848

************************************************************

NCF1B CCCACGTGGTAGACCTCTTCCCTCCTAGGGTAAATCAGCTTCTGTGTCAGGGATGCTGTG 4919

NCF1C CCCACGTGGTAGACCTCTTCCCTCCTAGGGTAAATCAGCTTCTGTGTCAGGGATGCTGTG 4907

NCF1 CCCACGTGGTAGACCTCTTCCCTCCTAGGGTAAATCAGCTTCTGTGTCAGGGATGCTGTG 4908 Myc

************************************************************

NCF1B TGGCGTCCATCTGAACCCCCTGCATACGCGTAGCTAATGTGATCTCCCCACTTTCACATA 4979

NCF1C TGGCGTCCATCTGAACCCCCTGCATACGCGTAGCTAATGTGATCTCCCCACTTTCACATA 4967

NCF1 TGGTGTCCATCTGAACCCCCTGCATACGCGTAGCTAATGTGATCTCCCCACTTTCACATA 4968 AML1 (NCF1-specific), MZF1

*** ********************************************************

NCF1B AGATGGTGGCCCTGCCTTCAGGGAATGTGGGAGCCAGGTGGGAGCCTTCCCGGATATTTA 5039

NCF1C AGATGGTGGCCCTGCCTTCAGGGAATGTGGGAGCCAGGTGGGAGCCTTCCCGGATATTTA 5027

NCF1 AGATGGTGGCCCTGCCTTCAGGGAATGTGGGAGCCAGGTGGGAGCCTTCCCGGATATTTA 5028 TEF1

************************************************************

NCF1B AGCTAGAAGATTCTACAGGGAGATTCTCCTTGGATCAATATATGTCTCTCAGTCAAAGAT 5099

NCF1C AGCTAGAAGATTCTACAGGGAGATTCTCCTTGGATCAATATATGTCTCTCAGTCAAAGAT 5087

NCF1 AGCTAGAAGATTCTACAGGGAGATTCTCCTTGGATCAATATATGTCTCTCAGTCAAAGAT 5088 LEF1

************************************************************

NCF1B GTAAAAGCACTTTTGCCTTAAAAAGAATGTTCTGTTTCTAAATAGAGTCAACGTTGTCCT 5159

NCF1C GTAAAAGCACTTTTGCCTTAAAAAGAATGTTCTGTTTCTAAATAGAGTCAACGTTGTCCT 5147

NCF1 GTAAAAGCACTTTTGCCTTAAAAAGAATGTTCTGTTTCTAAATAGAGTCAACGTTGTCCT 5148

************************************************************

NCF1B CCTCATTGGAATTCACTATGAGTCAGAATCATTAGACTGACTTTTTTTTTTCCATAGTAA 5219

NCF1C CCTCATTGGAATTCACTATGAGTCAGAATCATTAGACTGACTTTTTTTTTTCCATAGTAA 5207

NCF1 CCTCATTGGAATTCACTATGAGTCAGAATCATTAGACTGACTTTTTTTTTTCCATAGTAA 5208 AP1

************************************************************

NCF1B TAGTATTTTGCAGAGTCTCACAGAGCTGCAGATCTTTTGTTCATCTTGCAGAGTTAACAA 5279

NCF1C TAGTATTTTGCAGAGTCTCACAGAGCTGCAGATCTTTTGTTCATCTTGCAGAGTTAACAA 5267

NCF1 TAGTATTTTGCAGAGTCTCACAGAGCTGCAGATCTTTTGTTCATCTTGCAGAGTTAACAA 5268

************************************************************

NCF1B GTCTGATCCTGTTAGTCCAGATTTCTTAAATTTGGCCAAGTTATAATAGGAGCAGTAGCT 5339

NCF1C GTCTGATCCTGTTAGTCCAGATTTCTTAAATTTGGCCAAGTTATAATAGGAGCAGTAGCT 5327

NCF1 GTCTGATCCTGTTAGTCCAGATTTCTTAAATTTGGCCAAGTTATAATAGGAGCAGTAGCT 5328

************************************************************

NCF1B TGAGACCCGAAGTCAGGAAACTTTGACAATGGATTTTTTTTTTTTAATCCAGAGACTTGT 5399

NCF1C TGAGACCCGAAGTCAGGAAACTTTGACAATGGATTTTTTTTTTTTAATCCAGAGACTTGT 5387

NCF1 TGAGACCCGAAGTCAGGAAACTTTGACAATGGATTTTTTTTTTT-AATCCAGAGACTTGT 5387 LEF1

******************************************** ***************

NCF1B ACTGGAATTTGCCTTACCCTGTCAGCTCATGGACTTAAGGTTTCATCCCGCTTTATGAGT 5459

NCF1C ACTGGAATTTGCCTTACCCTGTCAGCTCATGGACTTAAGGTTTCATCCCGCTTTATGAGT 5447

NCF1 ACTGGAATTTGCCTTACCCTGTCAGCTCATGGACTTAAGGTTTCATCCCGCTTTATGAGT 5447

************************************************************

NCF1B GCTTCTGAATCCAAGTCATTGTTACCTGAATTTGCAAATTAAGTTGTGATATTCGTGACT 5519

NCF1C GCTTCTGAATCCAAGTCATTGTTACCTGAATTTGCAAATTAAGTTGTGATATTCGTGACT 5507

NCF1 GCTTCTGAATCCAAGTCATTGTTACCTGAATTTGCAAATTAAGTTGTGATATTCGTGACT 5507

************************************************************

NCF1B GTTAAATTCCTGTAATTAGATTAACCTCTTTGCTTGCTTGTTTGTTTTCTCTCCTATTTT 5579

NCF1C GTTAAATTCCTGTAATTAGATTAACCTCTTTGCTTGCTTGTTTGTTTTCTCTCCTATTTT 5567

NCF1 GTTAAATTCCTGTAATTAGATTAACCTCTTTGCTTGCTTGTTTGTTTTCTCTCCTATTTT 5567 SRY

************************************************************

NCF1B AGCTTAAAGTATCAGTGGTTGAGAAGAGCTTTTCGGACCTGTTACTACCCCAAGCTGTGT 5639

NCF1C AGCTTAAAGTATCAGTGGTTGAGAAGAGCTTTTCGGACCTGTTACTACCCCAAGCTGTGT 5627

NCF1 AGCTTAAAGTATCAGTGGTTGAGAAGAGCTTTTCGGACCTGTTACTACCCCAAGCTGTGT 5627

************************************************************

NCF1B AATATACTTGTATAACAGAAATACCTTCTATACAAACCTTTTTTTCTACTTTTAGATAGA 5699

NCF1C AATATACTTGTATAACAGAAATACCTTCTATACAAACCTTTTTTTCTACTTTTAGATAGA 5687

NCF1 AATATACTTGTATAACAGAAATACCTTCTATACAAACCTTTTTTTCTACTTTTAGATAGA 5687 Stat5A

************************************************************

NCF1B AATGTCTACTTTTTCAGCAGTTCTGTGAATTAAAGAGCAGAGTGACTGTGGGTCTGGAAT 5759

NCF1C AATGTCTACTTTTTCAGCAGTTCTGTGAATTAAAGAGCAGAGTGACTGTGGGTCTGGAAT 5747

NCF1 AATGTCTACTTTTTCAGCAGTTCTGTGAATTAAAGAGCAGAGTGACTGTGGGTCTGGAAT 5747 TEF1

************************************************************

NCF1B GGCTGGTGTACTTGGGAATGTACTATCAGGATTTTACAGCAATGCTGGGAAATGACAGGG 5819

NCF1C GGCTGGTGTACTTGGGAATGTACTATCAGGATTTTACAGCAATGCTGGGAAATGACAGGG 5807

NCF1 GGCTGGTGTACTTGGGAATGTACTATCAGGATTTTACAGCAATGCTGGGAAATGACAGGG 5807 TEF1

************************************************************

NCF1B AAAATGACAGGAATGAATCTCACCAGATTTTTTATGTACTCAGCAGAGCCTTGAGTTACG 5879

NCF1C AAAATGACAGGAATGAATCTCACCAGATTTTTTATGTACTCAGCAGAGCCTTGAGTTACG 5867

NCF1 AAAATGACAGGAATGAATCTCACCAGATTTTTTATGTACTCAGCAGAGCCTTGAGTTACG 5867 TEF1

************************************************************

NCF1B GTGTTTATTTTCCAATCAAGTGAAGATATCTCCTACTTCTCCTACTGGAACATCTCAGCT 5939

NCF1C GTGTTTATTTTCCAATCAAGTGAAGATATCTCCTACTTCTCCTACTGGAACATCTCAGCT 5927

NCF1 GTGTTTATTTTCCAATCAAGTGAAGATATCTCCTACTTCTCCTACTGGAACATCTCAGCT 5927 Nkx25, IRF1

************************************************************

NCF1B TCTGCAGTGAAGAAAAATTCCTGTGATAGTTCAGTTCTTTAGTTTTTCTATTTGAAAAAA 5999

NCF1C TCTGCAGTGAAGAAAAATTCCTGTGATAGTTCAGTTCTTTAGTTTTTCTATTTGAAAAAA 5987

NCF1 TCTGCAGTGAAGAAAAATTCCTGTGATAGTTCAGTTCTTTAGTTTTTCTATTTGAAAAAA 5987 HNF4

************************************************************

NCF1B AAAAATCATTTAAATGATCCTTTGTTCACGGCTCTCCTTAATGACTGAGTGAACAGTTCC 6059

NCF1C AAAAATCATTTAAATGATCCTTTGTTCACGGCTCTCCTTAATGACTGAGTGAACAGTTCC 6047

NCF1 AAAAATCATTTAAATGATCCTTTGTTCACGGCTCTCCTTAATGACTGAGTGAACAGTTCC 6047

************************************************************

NCF1B TATCTGTATATTTGACTAAACCTTTTCCTAAGCTATCTCTCATGGTTCCTATGTTTTTTT 6119

NCF1C TATCTGTATATTTGACTAAACCTTTTCCTAAGCTATCTCTCATGGTTCCTATGTTTTTTT 6107

NCF1 TATCTGTATATTTGACTAAACCTTTTCCTAAGCTATCTCTCATGGTTCCTATGTTTTTTT 6107

************************************************************

NCF1B ATCATAATTAAAAGCAAAACCATCTGGATCACCTAACAGTCAGAGGTCAGTATCTCAGCG 6179

NCF1C ATCATAATTAAAAGCAAAACCATCTGGATCACCTAACAGTCAGAGGTCAGTATCTCAGCG 6167

NCF1 ATCATAATTAAAAGCAAAACCATCTGGATCACCTAACAGTCAGAGGTCAGTATCTCAGCG 6167

************************************************************

NCF1B TGTGAATTATAGAGGAAATACAGAGAGAACCTCTTCCACTTTTACTTTTCGTCCAAATAA 6239

NCF1C TGTGAATTATAGAGGAAATACAGAGAGAACCTCTTCCACTTTTACTTTTCGTCCAAATAA 6227

NCF1 TGTGAATTATAGAGGAAATACAGAGAGAACCTCTTCCACTTTTACTTTTCGTCCAAATAA 6227 Stat5A

************************************************************

NCF1B AATGCATGGTGTACCAGAAGTTGAAGATCGGGTTGAGGATTGGGGCTAGCTCGATGACAC 6299

NCF1C AATGCATGGTGTACCAGAAGTTGAAGATCGGGTTGAGGATTGGGGCTAGCTCGATGACAC 6287

NCF1 AATGCATGGTGTACCAGAAGTTGAAGATCGGGTTGAGGATTGGGGCTAGCTCGATGACAC 6287

************************************************************

NCF1B TAAGGCCCCAACATCGCGGGACCTGCTGTGGCGCGGATTCTTAGGAACGCTGTTCTAGCC 6359

NCF1C TAAGGCCCCAACATCGCGGGACCTGCTGTGGCGCGGATTCTTAGGAACGCTGTTCTAGCC 6347

NCF1 TAAGGCCCCAACATCGCGGGACCTGCTGTGGCGCGGATTCTTAGGAACGCTGTTCTAGCC 6347 E2F

************************************************************

NCF1B GGCCCCCTCTCCAGGGGTCGCCGTGGCCGGCATTATTTCCTAGTTCTTCTTGTAACCCTG 6419

NCF1C GGCCCCCTCTCCAGGGGTCGCCGTGGCCGGCATTATTTCCTAGTTCTTCTTGTAACCCTG 6407

NCF1 GGCCCCCTCTCCAGGGGTCGCCGTGGCCGGCATTATTTCCTAGTTCTTCTTGTAACCCTG 6407 Stat5A

************************************************************

NCF1B AGGTGCCAGCGCGGGGAGTGAGGAGGGGTCAGGGGGCTAAGGATGCAACCTCTGACGTTC 6479

NCF1C AGGTGCCAGCGCGGGGAGTGAGGAGGGGTCAGGGGGCTAAGGATGCAACCTCTGACGTTC 6467

NCF1 AGGTGCCAGCGCGGGGAGTGAGGAGGGGTCAGGGGGCTAAGGATGCAACCTCTGACGTTC 6467 LFA1

************************************************************

NCF1B TGCGCCTTCCTAGGAGAGTCTTACATGTGTTGAGATTTCACAAGCAATGCGAGTTGTAAA 6539

NCF1C TGCGCCTTCCTAGGAGAGTCTTACATGTGTTGAGATTTCACAAGCAATGCGAGTTGTAAA 6527

NCF1 TGCGCCTTCCTAGGAGAGTCTTACATGTGTTGAGATTTCACAAGCAATGCGAGTTGTAAA 6527

************************************************************

NCF1B ATACCAGCTCTACAAGAAGCTAGGCTCTGTGACGGCATAGTTTTCAGTAGCTTTATCACA 6599

NCF1C ATACCAGCTCTACAAGAAGCTAGGCTCTGTGACGGCATAGTTTTCAGTAGCTTTATCACA 6587

NCF1 ATACCAGCTCTACAAGAAGCTAGGCTCTGTGACGGCATAGTTTTCAGTAGCTTTATCACA 6587

************************************************************

NCF1B ATATTCACAATGGAGAATTATATGACATGGTAGCAGAAATAGGCCCTTTTATGTGTTGCT 6659

NCF1C ATATTCACAATGGAGAATTATATGACATGGTAGCAGAAATAGGCCCTTTTATGTGTTGCT 6647

NCF1 ATATTCACAATGGAGAATTATATGACATGGTAGCAGAAATAGGCCCTTTTATGTGTTGCT 6647

************************************************************

NCF1B TCTATTTTACCTCAAATTGTAGATATAGGGTAATCAATAAAATCCATCCATGCCTTTCAC 6719

NCF1C TCTATTTTACCTCAAATTGTAGATATAGGGTAATCAATAAAATCCATCCATGCCTTTCAC 6707

NCF1 TCTATTTTACCTCAAATTGTAGATATAGGGTAATCAATAAAATCCATCCATGCCTTTCAC 6707

************************************************************

NCF1B ACACTAAGTCATTGCTCTCTCGGCTGTTTTCATGGTCCTGTCTGGGGAAGCTTGGGGGTG 6779

NCF1C ACACTAAGTCATTGCTCTCTCGGCTGTTTTCATGGTCCTGTCTGGGGAAGCTTGGGGGTG 6767

NCF1 ACACTAAGTCATTGCTCTCTCGGCTGTTTTCATGGTCCTGTCTGGGGAAGCTTGGGGGTG 6767

************************************************************

NCF1B GCTCGGCGTAGGTGGGACGCAGACCAAGGCCGAGGCTGGCGCTGGGCAGAGCCCGCCGGG 6839

NCF1C GCTCGGCGTAGGTGGGACGCAGACCAAGGCCGAGGCTGGCGCTGGGCAGAGCCCGCCGGG 6827

NCF1 GCTCGGCGTAGGTGGGACGCAGACCAAGGCCGAGGCTGGCGCTGGGCAGAGCCCGCCGGG 6827

************************************************************

NCF1B CCTCCCGGGGACAGCGCCACTTGCGGTGTTTCTCAGACCGCTGCTGCCCATCTCCTCTGA 6899

NCF1C CCTCCCGGGGACAGCGCCACTTGCGGTGTTTCTCAGACCGCTGCTGCCCATCTCCTCTGA 6887

NCF1 CCTCCCGGGGACAGCGCCACTTGCGGTGTTTCTCAGACCGCTGCTGCCCATCTCCTCTGA 6887

************************************************************

NCF1B GCGGGCCAGGGCCCCCACCCTTCCATCTGGGCCATGCCAGCTGTGTATGGAGAGCCGCTC 6959

NCF1C GCGGGCCAGGGCCCCCACCCTTCCATCTGGGCCATGCCAGCTGTGTATGGAGAGCCGCTC 6947

NCF1 GCGGGCCAGGGCCCCCACCCTTCCATCTGGGCCATGCCAGCTGTGTATGGAGAGCCGCTC 6947 HEB

************************************************************

NCF1B ACATACGACATTGGGTGCCAAAAGCCCCTGCCATCGAGAGCTCATGCAGCAGTCCCTCCT 7019

NCF1C ACATACGACATTGGGTGCCAAAAGCCCCTGCCATCGAGAGCTCATGCAGCAGTCCCTCCT 7007

NCF1 ACATACGACATTGGGTGCCAAAAGCCCCTGCCATCGAGAGCTCATGCAGCAGTCCCTCCT 7007

************************************************************

NCF1B GCCTGAGCCCACACACTGACTCTGAGGCTCTTCTGTTCTCAGCGTGGTCCCTGCCTCTGC 7079

NCF1C GCCTGAGCCCACACACTGACTCTGAGGCTCTTCTGTTCTCAGCGTGGTCCCTGCCTCTGC 7067

NCF1 GCCTGAGCCCACACACTGACTCTGAGGCTCTTCTGTTCTCAGCGTGGTCCCTGCCTCTGC 7067 GR

************************************************************

NCF1B CGTGCCCTATCCGCGTGTGCCAGAAAGGGAAACTGATCTCACGATTCACCTGCCTGCTAA 7139

NCF1C CGTGCCCTATCCGCGTGTGCCAGAAAGGGAAACTGATCTCACGATTCACCTGCCTGCTAA 7127

NCF1 CGTGCCCTATCCGCGTGTGCCAGAAAGGGAAACTGATCTCACGATTCACCTGCCTGCTAA 7127 **E2A (myogenin)**

************************************************************

NCF1B CCTGGGAGGAGACTGCTTCTCTGATAGCATCTCACAGTTCTTTTAACATGTTCTTAAAAT 7199

NCF1C CCTGGGAGGAGACTGCTTCTCTGATAGCATCTCACAGTTCTTTTAACATGTTCTTAAAAT 7187

NCF1 CCTGGGAGGAGACTGCTTCTCTGATAGCATCTCACAGTTCTTTTAACATGTTCTTAAAAT 7187

************************************************************

NCF1B GTGTCCTGCCGCGCCACCCTCAGCCACACCTCCCAGCACCCATCCTGGAGAATCAGGGCC 7259

NCF1C GTGTCCTGCCGCGCCACCCTCAGCCACACCTCCCAGCACCCATCCTGGAGAATCAGGGCC 7247

NCF1 GTGTCCTGCCGCGCCACCCTCAGCCACACCTCCCAGCACCCATCCTGGAGAATCAGGGCC 7247 E2F

************************************************************

NCF1B ATGGCAAGGCTGCCGTTGGCAAAAGCCCAGCTCTGCCATGAATCTCCGTTCAGTTACTGG 7319

NCF1C ATGGCAAGGCTGCCGTTGGCAAAAGCCCAGCTCTGCCATGAATCTCCGTTCAGTTACTGG 7307

NCF1 ATGGCAAGGCTGCCGTTGGCAAAAGCCCAGCTCTGCCATGAATCTCCGTTCAGTTACTGG 7307

************************************************************

NCF1B TTTCACACTCATTTGTGAGGTCACAGACTTGCCAAGGATATTCATGATTCAATCCATTAC 7379

NCF1C TTTCACACTCATTTGTGAGGTCACAGACTTGCCAAGGATATTCATGATTCAATCCATTAC 7367

NCF1 TTTCACACTCATTTGTGAGGTCACAGACTTGCCAAGGATATTCATGATTCAATCCATTAC 7367

************************************************************

NCF1B AACAATTCTGTGTGTACATAACACATTTTTTCCTTTCTTTTTTTTTTT------TTTTTC 7433

NCF1C AACAATTCTGTGTGTACATAACACATTTTTTCCTTTCTTTTTTTTTTTTTTTTTTTTTTC 7427

NCF1 AACAATTCTGTGTGTACATAACACATTTTTTCCTTTCTTTTTTTTTTTTTTT--TTTTTC 7425

************************************************ ******

NCF1B CTGAGACAGTCTTGCTCTGTTGCCCAGGCTAGAGCACAGTGGTGCAGTCTCAGCTCACTG 7493

NCF1C CTGAGACAGTCTTGCTCTGTTGCCCAGGCTAGAGCACAGTGGTGCAGTCTCAGCTCACTG 7487

NCF1 CTGAGACAGTCTTGCTCTGTTGCCCAGGCTAGAGCACAGTGGTGCAGTCTCAGCTCACTG 7485

************************************************************

NCF1B CATCCTCCACCTCACAGGTTCAAGCAATTCTCCTGCCTCAGCCTCCCGAGTAGCTGGGAT 7553

NCF1C CATCCTCCACCTCACAGGTTCAAGCAATTCTCCTGCCTCAGCCTCCCGAGTAGCTGGGAT 7547

NCF1 CATCCTCCACCTCACAGGTTCAAGCAATTCTCCTGCCTCAGCCTCCCGAGTAGCTGGGAT 7545

************************************************************

NCF1B TGCAGGCACCCACCAACACACCTGACTAATTTTTGTATTTTTAGTAGAGACGGGGTTTCA 7613

NCF1C TGCAGGCACCCACCAACACACCTGACTAATTTTTGTATTTTTAGTAGAGACGGGGTTTCA 7607

NCF1 TGCAGGCACCCACCAACACACCTGACTAATTTTTGTATTTTTAGTAGAGACGGGGTTTCA 7605

************************************************************

NCF1B GCATGTTGGCCAGGCTGGTCTCAAAATCCTGGCCTCAGGTATATACACATATTTTTACAC 7673

NCF1C GCATGTTGGCCAGGCTGGTCTCAAAATCCTGGCCTCAGGTATATACACATATTTTTACAC 7667

NCF1 GCATGTTGGCCAGGCTGGTCTCAAAATCCTGGCCTCAGGTATATACACATATTTTTACAC 7665

************************************************************

NCF1B ACACACACACACACACACACACACACA----TAAAGGATTTAAGCCAGGTGCTGTGGTGT 7729

NCF1C ACACACACACACACACACACACACACA----TAAAGGATTTAAGCCAGGTGCTGTGGTGT 7723

NCF1 ACACACACACACACACACACACACACACACATAAAGGATTTAAGCCAGGTGCTGTGGTGT 7725 AML1

*************************** *****************************

NCF1B ACAACTGTAGTCCCAGCTACTAGGGAGGCTGAGGTGGGAGGATCACTTGAGCCACTGCAG 7789

NCF1C ACAACTGTAGTCCCAGCTACTAGGGAGGCTGAGGTGGGAGGATCACTTGAGCCACTGCAG 7783

NCF1 ACAACTGTAGTCCCAGCTACTAGGGAGGCTGAGGTGGGAGGATCACTTGAGCCACTGCAG 7785 Nkx25

************************************************************

NCF1B TGATTACACCACTGCACTCCAGACTGGGTGACAGTGAGACCCTGTCTCTTAAAAGAAAAA 7849

NCF1C TGATTACACCACTGCACTCCAGACTGGGTGACAGTGAGACCCTGTCTCTTAAAAGAAAAA 7843

NCF1 TGATTACACCACTGCACTCCAGACTGGGTGACAGTGAGACCCTGTCTCTTAAAAGAAAAA 7845

************************************************************

NCF1B AGAAGAGGCCTTAGCTGCCTGCATTAATTAATGAACAGGCAGTGTATTTCTGTTCTTTTT 7909

NCF1C AGAAGAGGCCTTAGCTGCCTGCATTAATTAATGAACAGGCAGTGTATTTCTGTTCTTTTT 7903

NCF1 AGAAGAGGCCTTAGCTGCCTGCATTAATTAATGAACAGGCAGTGTATTTCTGTTCTTTTT 7905 GR

************************************************************

NCF1B TTTTTTTTTTT---GAAGACGGAGTCTCACTCTGTTGCCCAGGCTGGAGTGCAGTGGTGC 7966

NCF1C TTTTTTTTTTTTTTGAAGACGGAGTCTCACTCTGTTGCCCAGGCTGGAGTGCAGTGGTGC 7963

NCF1 TTTTTTTTTTTTTTGAAGACGGAGTCTCACTCTGTTGCCCAGGCTGGAGTGCAGTGGTGC 7965

*********** **********************************************

NCF1B AATCTTGGCTCACTGTAACCTCCGCCTCCTGAGTTCGAGATTCTCCTGTCTCAGCCACCC 8026

NCF1C AATCTTGGCTCACTGTAACCTCCGCCTCCTGAGTTCGAGATTCTCCTGTCTCAGCCACCC 8023

NCF1 AATCTTGGCTCACTGTAACCTCCGCCTCCTGAGTTCGAGATTCTCCTGTCTCAGCCACCC 8025

************************************************************

NCF1B CAGTAACTGTGATTATGGCCACCTGCCACCATGCCCGGCTAATTTTTGTATTTTTAGTAG 8086

NCF1C CAGTAACTGTGATTATGGCCACCTGCCACCACGCCCGGCTAATTTTTGTATTTTTAGTAG 8083

NCF1 CAGTAACTGTGATTATGGCCACCTGCCACCATGCCCGGCTAATTTTTGTATTTTTAGTAG 8085 SREBP1, **E2A (myogenin)**

******************************* ****************************

NCF1B AGACGGGGTTTCCCCATGTCGGCCAAGCTGGTCTCGAACTCCCAACCTCAAGTGATCCAC 8146

NCF1C AGACGGGGTTTCCCCATGTCGGCCAAGCTGGTCTCGAACTCCCAACCTCAAGTGATCCAC 8143

NCF1 AGACGGGGTTTCCCCATGTCGGCCAAGCTGGTCTCAAACTCCCAACCTCAAGTGATCCAC 8145 Nkx25, **E2A (myogenin)**

*********************************** ************************

NCF1B CTGCCTCGGCCTCCCAAAGTGCTGGGATGACAGGTGTGAGCCACCAAACCCAGCCTCAGG 8206

NCF1C CTGCCTCGGCCTCCCAAAGTGCTGGGATGACAGGTGTGAGCCACCAAACCCAGCCTCAGG 8203

NCF1 CTGCCTCGGCCTCCCAAAGTGCTGGGATGACAGGTGTGAGCCACCAAACCCAGCCTCAGG 8205 **E2A**

************************************************************

NCF1B CAGTGCATTTCTAAAAGGCAGATAGTGTGATTGTTCAGAGGGTGAAGGGGAAGTTAAACT 8266

NCF1C CAGTGCATTTCTAAAAGGCAGATAGTGTGATTGTTCAGAGGGTGAAGGGGAAGTTAAACT 8263

NCF1 CAGTGCATTTCTAAAAGGCAGATAGTGTGATTGTTCAGAGGGTGAAGGGGAAGTTAAACT 8265 Stat5A

************************************************************

NCF1B TGTCCAGTAAAATCTTCGTTAGCCCGGTATTCTGGATTGAAAAAGCAAAATATAGTTCAA 8326

NCF1C TGTCCAGTAAAATCTTCGTTAGCCCGGTATTCTGGATTGAAAAAGCAAAATATAGTTCAA 8323

NCF1 TGTCCAGTAAAATCTTCGTTAGCCCGGTATTCTGGATTGAAAAAGCAAAATATAGTTCAA 8325 HNF4

************************************************************

NCF1B GTAGGTCTCTTCAGTGTATCTAATAAGCTCTTGTTTCTGAAACAACTGATTCCTTGGCCG 8386

NCF1C GTAGGTCTCTTCAGTGTATCTAATAAGCTCTTGTTTCTGAAACAACTGATTCCTTGGCCG 8383

NCF1 GTAGGTCTCTTCAGTGTATCTAATAAGCTCTTGTTTCTGAAACAACTGATTCCTTGGCCG 8385

************************************************************

NCF1B GGCGCGGTGGCTCACGCCTGTAATTCCAGCACTTTGGGAGGCCAAGGAGGGCAGATCACT 8446

NCF1C GGCGCGGTGGCTCACGCCTGTAATTCCAGCACTTTGGGAGGCCAAGGAGGGCAGATCACT 8443

NCF1 GGCGCGGTGGCTCACGCCTGTAATTCCAGCACTTTGGGAGGCCAAGGAGGGCAGATCACT 8445 E2A, Nkx25

************************************************************

NCF1B TGAGGTCGGGAGCTCAAGACCAGCCTGGTCAACATGGTGAAACCTCGTCTCTAGTGAAAA 8506

NCF1C TGAGGTCGGGAGCTCAAGACCAGCCTGGTCAACATGGTGAAACCTCGTCTCTAGTGAAAA 8503

NCF1 TGAGGTCGGGAGCTCAAGACCAGCCTGGTCAACATGGTGAAACCTCGTCTCTAGTGAAAA 8505

************************************************************

NCF1B TACAAAAATTAGCCGACTGTGGTAGTGCACACCTGTAATCCCAGCTACTCGGGAGGCTGA 8566

NCF1C TACAAAAATTAGCCGACTGTGGTAGTGCACACCTGTAATCCCAGCTACTCGGGAGGCTGA 8563

NCF1 TACAAAAATTAGCCGACTGTGGTAGTGCACACCTGTAATCCCAGCTACTCGGGAGGCTGA 8565 AML1

************************************************************

NCF1B GGTAGGAGAGAATTGCTTGAACCTGGGAGGCGGAGGCTGCAGTGAGCCAAGATCACACCA 8626

NCF1C GGTAGGAGAGAATTGCTTGAACCTGGGAGGCGGAGGCTGCAGTGAGCCAAGATCACACCA 8623

NCF1 GGTAGGAGAGAATTGCTTGAACCTGGGAGGCGGAGGCTGCAGTGAGCCAAGATCACACCA 8625

************************************************************

NCF1B CTGCACTACAGCCTGCGTGACAGAGTGAAACTCTGTCTCAAAAAAACAAAAACCTCCTGA 8686

NCF1C CTGCACTACAGCCTGCGTGACAGAGTGAAACTCTGTCTCAAAAAAACAAAAACCTCCTGA 8683

NCF1 CTGCACTACAGCCTGCGTGACAGAGTGAAACTCTGTCTCAAAAAAACAAAAACCTCCTGA 8685 SRY

************************************************************

NCF1B TTTTTTTTTTTTTTTTTTTTTTT-----GAGATGGAGTCTGGCTCTGTCGCCCAGGCTGG 8741

NCF1C TTTTTTTTTTTTTTTTTTTTTTT-----GAGATGGAGTCTGGCTCTGTCGCCCAGGCTGG 8738

NCF1 TTTTTTTTTTTTTTTTTTTTTTTTTTTTGAGATGGAGTCTGGCTCTGTCGCCCAGGCTGG 8745 FOXM1

*********************** ********************************

NCF1B AGCGCAATGGTGCGATCTCGGCTCACTGCAACCTCTGCCTTCTGGATTCAAACGATTCTC 8801

NCF1C AGCGCAATGGTGCGATCTCGGCTCACTGCAACCTCTGCCTTCTGGATTCAAACGATTCTC 8798

NCF1 AGCGCAATGGTGCGATCTCGGCTCACTGCAACCTCTGCCTTCTGGATTCAAACGATTCTC 8805

************************************************************

NCF1B CTGCCTCAGCCTCCAGAGTATCTGGAACTACAGGTGCCTGCCACCACGCCCAGCTAATTT 8861

NCF1C CTGCCTCAGCCTCCAGAGTATCTGGAACTACAGGTGCCTGCCACCACGCCCAGCTAATTT 8858

NCF1 CTGCCTCAGCCTCCAGAGTATCTGGAACTACAGGCGCCTGCCACCACGCCCAGCTAATTT 8865

********************************** *************************

NCF1B TTTGTATTTTTAGTAGAGATGGGGTTTCACCATGTTAGCCTGGATGGTCTCGATCTCCTG 8921

NCF1C TTTGTATTTTTAGTAGAGATGGGGTTTCACCATGTTAGCCTGGATGGTCTCGATCTCCTG 8918

NCF1 TTTGTATTTTTAGTAGAGATGGGGTTTCACCATGTTAGCCTGGATGGTCTCGATCTCCTG 8925

************************************************************

NCF1B ACCTCGTGATTTGCCCACCTCGGCCTCCGAAAGTGTTGGGATTACAGGCATGAGCCGCCA 8981

NCF1C ACCTCGTGATTTGCCCACCTCGGCCTCCGAAAGTGTTGGGATTACAGGCATGAGCCACCA 8978

NCF1 ACCTCGTGATTTGCCCACCTCGGCCTCCGAAAGTGTTGGGATTACAGGCATGAGCCGCCA 8985 Pitx2

******************************************************** ***

NCF1B CACCCGGCCGCCTCCTGATTTCATAATAATTCAAGGGACAAACATAATTACTTCCCAGGT 9041

NCF1C CACCCGGCCGCCTCCTGATTTCATAATAATTCAAGGGACAAACATAATTACTTCCCAGGT 9038

NCF1 CACCCGGCCGCCTCCTGATTTCATAATAATTCAAGGGACAAACATAATTACTTCCCAGGT 9045

************************************************************

NCF1B AAGGCAGTAGAGTGACTAAGAACAAATCCACTTCTGGACAGCCATTATATAGGGGACATT 9101

NCF1C AAGGCAGTAGAGTGACTAAGAACAAATCCACTTCTGGACAGCCATTATATAGGGGACATT 9098

NCF1 AAGGCAGTAGAGTGACTAAGAACAAATCCACTTCTGGACAGCCATTATATAGGGGACATT 9105

************************************************************

NCF1B TTTATCAGAAAAACAGTAAAAGCAAGGAAATGTCTCCCTCCCAGGACTGTATGAATTAAA 9161

NCF1C TTTATCAGAAAAACAGTAAAAGCAAGGAAATGTCTCCCTCCCAGGACTGTGTGAATTAAA 9158

NCF1 TTTATCAGAAAAACAGTAAAAGCAAGGAAATGTCTCCCTCCCAGGACTGTATGAATTAAA 9165

************************************************** *********

NCF1B CCCTGCCTGCTGATTGCCAAGTGACAACTCCCATTCCAAGCCCCTGGTCCTCAGTGGGGA 9221

NCF1C CCCTGCCTGCTGATTGCCAAGTGACAACTCCCATTCCAAGCCCCTGGTCCTCAGTGGGGA 9218

NCF1 CCCTGCCTGCTGATTGCCAAGTGACAACTCCCATTCCAAGCCCCTGGTCCTCAGTGGGGA 9225 TEF1, MZF1

************************************************************

NCF1B GGAAACCAAAGCTTTGTCCCTGGAGGGAGGTCCTGCGGGAATGGCCAGGGACGCATCCGC 9281

NCF1C GGAAACCAAAGCTTTGTCCCTGGAGGGAGGTCCTGCGGGAATGGCCAGGGACGCATCCGC 9278

NCF1 GGAAACCAAAGCTTTGTCCCTGGAGGGAGGTCCTGCGGGAATGGCCAGGGACGCATCCGC 9285 TFIII, TEF1

************************************************************

NCF1B TCTCAAATGCTAGAGCTGGCAGTTGTCATCTGCTCGTGAGAAGGTTTCGCCTTTGCCTGT 9341

NCF1C TCTCAAATGCTAGAGCTGGCAGTTGTCATCTGCTCGTGAGAAGGTTTCGCCTTTGCCTGT 9338

NCF1 TCTCAAATGCTAGAGCTGGCAGTTGTCATCTGCTCGTGAGAAGGTTTCGCCTTTGCCTGT 9345

************************************************************

NCF1B CCCCACCCCCGTCCCTCCCAGGCCATCAGCGCACATCAGAGTTAGCACATTACATGATGC 9401

NCF1C CCCCACCCCCGTCCCTCCCAGGCCATCAGCGCACATCAGAGTTAGCACATTACATGATGC 9398

NCF1 CCCCACCCCCGTCCCTCCCAGGCCATCAGCGCACATCAGAGTTAGCACATTACATGATGC 9405

************************************************************

NCF1B CTTAATCTTCTAATTGGTTTAAGTCAGCTGACATGTAAAGTGAGGTCTGGGAGACTGTTC 9461

NCF1C CTTAATCTTCTAATTGGTTTAAGTCAGCTGACATGTAAAGTGAGGTCTGGGAGACTGTTC 9458

NCF1 CTTAATCTTCTAATTGGTTTAAGTCAGCTGACATGTAAAGTGAGGTCTGGGAGACTGTTC 9465

************************************************************

NCF1B TGGAACTGTATAGATGAGGACGTTGTTGCCCCAGTCTTATTCAGAAGCTTGAGGATGCAA 9521

NCF1C TGGAACTGTATAGATGAGGACGTTGTTGCCCCAGTCTTATTCAGAAGCTTGAGGATGCAA 9518

NCF1 TGGAACTGTATAGATGAGGACGTTGTTGCCCCAGTCTTATTCAGAAGCTTGAGGATGCAA 9525 HNF4

************************************************************

NCF1B CTTGGACCTGGGTCTGTGGTCCCCTAGGACCTGAAACTCATATAGAGCCAGCCGCCAGTT 9581

NCF1C CTTGGACCTGGGTCTGTGGTCCCCTAGGACCTGAAACTCATATAGAGCCAGCCGCCAGTT 9578

NCF1 CTTGGACCTGGGTCTGTGGTCCCCTAGGACCTGAAACTCATATAGAGCCAGCCGCCAGTT 9585 AML1

************************************************************

NCF1B CCATTTTGAAGTAGGTTGGTCTCTCTCTCT------ATTTTTTGAGACAGAGTTTCGCTC 9635

NCF1C CCATTTTGAAGTAGGTTGGTCTCTCTCTCTCTTTTTATTTTTTGAGACAGAGTTTCGCTC 9638

NCF1 CCATTTTGAAGTAGGTTGGTCTCTCTCTCTCTTTTTATTTTTTGAGACAGAGTTTCGCTC 9645

****************************** ************************

NCF1B TTGTTGCCCAGGCTGGAGTGCAATGGCACGATCTCAGCTCACCGCAACCTCCACCTCCCA 9695

NCF1C TTGTTGCCCAGGCTGGAGTGCAATGGCACGATCTCAGCTCACCGCAACCTCCACCTCCCA 9698

NCF1 TTGTTGCCCAGGCTGGAGTGCAATGGCACGATCTCAGCTCACCGCAACCTCCACCTCCCA 9705 HNF4

************************************************************

NCF1B GTTCAAGCGATTCTCCTGCTTCAGCCTCCCGAGTAGCTGGGATTACAGGCATGTGCCACC 9755

NCF1C GTTCAAGCGATTCTCCTGCTTCAGCCTCCCGAGTAGCTGGGATTACAGGCATGTGCCACC 9758

NCF1 GTTCAAGCGATTCTCCTGCTTCAGCCTCCCGAGTAGCTGGGATTACAGGCATGTGCCACC 9765

************************************************************

NCF1B ACACCTGGCTAGTTTTGTATTTTTAGTAGAGATGGGGTTTCTCCATGTTGGTCAGGCTGG 9815

NCF1C ACACCTGGCTAGTTTTGTATTTTTAGTAGAGATGGGGTTTCTCCATGTTGGTCAGGCTGG 9818

NCF1 ACACCTGGCTAGTTTTGTATTTTTAGTAGAGATGGGGTTTCTCCATGTTGGTCAGGCTGG 9825 AML1

************************************************************

NCF1B TCTGGAACTCCTGACCTCAAGCAATCCGCCCGCCCCAGCCTCCCAAAGTGCTGGGATAAC 9875

NCF1C TCTGGAACTCCTGACCTCAAGCAATCCGCCCGCCCCAGCCTCCCAAAGTGCTGGGATAAC 9878

NCF1 TCTGGAACTCCTGACCTCAAGCAATCCGCCCGCCCCAGCCTCCCAAAGTGCTGGGATAAC 9885

************************************************************

NCF1B AGGCATGAGCCCCTGTGCCTGGCTGTAGTTTGGTTTTTCTGAGCCTCCTCCTGGTTCCCA 9935

NCF1C AGGCATGAGCCCCTGTGCCTGGCTGTAGTTTGGTTTTTCTGAGCCTCCTCCTGGTTCCCA 9938

NCF1 AGGCATGAGCCCCTGTGCCTGGCTGTAGTTTGGTTTTTCTGAGCCTCCTCCTGGTTCCCA 9945

************************************************************

NCF1B TTTCTGTTTTTTTGTTTTTGTTTTTGTTTTTTCTCCAAAGACCAAAAGTTCCACCAGAGA 9995

NCF1C TTTCTGTTTTTTTGTTTTTGTTTTTGTTTTTTCTCCAAAGACCAAAAGTTCCACCAGAGA 9998

NCF1 TTTCTGTTTTTTTGTTTTTGTTTTTGTTTTTTCTCCAAAGACCAAAAGTTCCACCAGAGA 10005 SRY

************************************************************

NCF1B GGAAGATCGAGGGACCAGGCCTTTCTAGTTTCCACATTACACTCTAAGTGCTGGTCTTAG 10055

NCF1C GGAAGATCGAGGGACCAGGCCTTTCTAGTTTCCACATTACACTCTAAGTGCTGGTCTTAG 10058

NCF1 GGAAGATCGAGGGACCAGGCCTTTCTAGTTTCCACATTACACTCTAAGTGCTGGTCTTAG 10065

************************************************************

NCF1B TAAATTCAAGGCACCTGGTGGGCTTGACCATTCGGGGGCAGATAATTGTTACACACCAAA 10115

NCF1C TAAATTCAAGGCACCTGGTGGGCTTGACCATTCGGGGGCAGATAATTGTTACACACCAAA 10118

NCF1 TAAATTCAAGGCACCTGGTGGGCTTGACCATTCGGGGGCAGATAATTGTTACACACCAAA 10125 HNF4

************************************************************

NCF1B GGGGCATCTTTTGGAAAGTCACTGCCCAGTAACCACTTCCATCTTCTGGAAGGTCGCTGC 10175

NCF1C GGGGCATCTTTTGGAAAGTCACTGCCCAGTAACCACTTCCATCTTCTGGAAGGTCGCTGC 10178

NCF1 GGGGCATCTTTTGGAAAGTCACTGCCCAGTAACCACTTCCATCTTCTGGAAGGTCGCTGC 10185 HNF4

************************************************************

NCF1B TCATCTTCCTAAATGGAAGCCCCAGTTTCTGGACTTGGATGTGTTTTGAGGATCTGATGT 10235

NCF1C TCATCTTCCTAAATGGAAGCCCCAGTTTCTGGACTTGGATGTGTTTTGAGGATCTGATGT 10238

NCF1 TCATCTTCCTAAATGGAAGCCCCAGTTTCTGGACTTGGATGTGTTTTGAGGATCTGATGT 10245

************************************************************

NCF1B TCTCCCAAAGTGCCTCAGTTTCCCTATGATGGGGAAAGAGGAAGGGGACGGATTTTAGGA 10295 LYF1

NCF1C TCTCCCAAAGTGCCTCAGTTTCCCTATGATGGGGAAAGAGGAAGGGGACGGATTTTAGGA 10298

NCF1 TCTCCCAAAGTGCCTCAGTTTCCCTATGATGGGGAAAGAGGAAGGGGACGGATTTTAGGA 10305

************************************************************

NCF1B ATGGAGGTGACCTGGAGGCCGCTGTCCCTGTCCTTAGACCTGCGAGTCCAGGGGGATGAC 10355

NCF1C ATGGAGGTGACCTGGAGGCCGCTGTCCCTGTCCTTAGACCTGCGAGTCCAGGGGGATGAC 10358

NCF1 ATGGAGGTGACCTGGAGGCCGCTGTCCCTGTCCTTAGACCTGCGAGTCCAGGGGGATGAC 10365 TEF1, T3R

************************************************************

NCF1B CGCAAACAGGGCTGTGGGGCCTTTCTTTACTCTCAAAAGCATCACTTCCCCTGCCTGGAG 10415

NCF1C CGCAAACAGGGCTGTGGGGCCTTTCTTTACTCTCAAAAGCATCACTTCCCCTGCCTGGAG 10418

NCF1 CGCAAACAGGGCTGTGGGGCCTTTCTTTACTCTCAAAAGCATCACTTCCCCTGCCTGGAG 10425 HNF4

************************************************************

NCF1B TTCAGATCCTGCCTGGATCCACGGTGGGAAGGGAGCCCTGGCTCTCTGTACTTCACCCAC 10475

NCF1C TTCAGATCCTGCCTGGATCCACGGTGGGAAGGGAGCTCTGGCTCTCTGTACTTCACCCAC 10478

NCF1 TTCAGATCCTGCCTGGATCCACGGTGGGAAGGGAGCCCTGGCTCTCTGTACTTCACCCAC 10485

************************************ ***********************

NCF1B GGCTGCCCACTCACCTGGCTCACAGGGCAGACTGGATGCAGCTTTCAGCCAGTTGTAGAA 10535

NCF1C GGCTGCCCACTCACCTGGCTCACAGGGCAGACTGGATGCAGCTTTCAGCCAGTTGTAGAA 10538

NCF1 GGCTGCCCACTCACCTGGCTCACAGGGCAGACTGGATGCAGCTTTCAGCCAGTTGTAGAA 10545

************************************************************

NCF1B ATCACAGGTCCCTGGCCGGGAACGGTGACTCAGGCCTGTAATCCCAGCACTTTGGGAGGC 10595

NCF1C ATCACAGGTCCCTGGCCGGGAACGGTGACTCAGGCCTGTAATCCCAGCACTTTGGGAGGC 10598

NCF1 ATCACAGGTCCCTGGCTGGGAACAGTGACTCAGGCCTGTAATCCCAGCACTTTGGGAGGC 10605

**************** ****** ************************************

NCF1B CGAGGCGGGCGGATCATGAGGTCAGGAGATCGAGACCATCCTGGCTAGCACGGTGAAACC 10655

NCF1C CGAGGCGGGCGGATCATGAGGTCAGGAGATCGAGACCATCCTGGCTAGCACGGTGAAACC 10658

NCF1 CGAGGCGGGCGGATCATGAGGTCAGGAGATCGAGACCATCCTGGCTAGCACGGTGAAACC 10665

************************************************************

NCF1B CCGTCTCTACTAAAAATACAAAAAATTAGCCGGGCATGGTGCTGGGCGCCTATAGTCCCA 10715

NCF1C CCGTCTCTACTAAAAATACAAAAAATTAGCCGGGCATGGTGCTGGGCGCCTATAGTCCCA 10718

NCF1 CCGTCTCTACTAAAAATACAAAAAATTAGCCGGGCATGGTGCTGGGCGCCTATAGTCCCA 10725

************************************************************

NCF1B GCTACTCGGGAGGCTGAGGCAGAATGGCGTGAACCTGGGAGGCCAAGCTGGCAGTGAGCC 10775

NCF1C GCTACTCGGGAGGCTGAGGCAGAATGGCGTGAACCTGGGAGGCCAAGCTGGCAGTGAGCC 10778

NCF1 GCTACTCGGGAGGCTGAGGCAGAATGGCGTGAACCTGGGAGGCCAAGCTGGCAGTGAGCC 10785

************************************************************

NCF1B GAGATCGCACCACTGCACTCTAGCCTGGGTGACAGAGCGAGACTCCGTCTCAAAAAAAAG 10835

NCF1C GAGATCGCACCACTGCACTTCAGCCTGGGTGACAGAGCGAGACTCCGTCTCAAAAAAAAG 10838

NCF1 GAGATCGCACCACTGCACTCTAGCCTGGGTGACAGAGCGAGACTCCGTCTCAAAAAAAAG 10845

******************* ***************************************

NCF1B AAATCACAGGTCCCTAGGGTCCTAGTGGCCCATCGGTGACAAAGGGCAGGTGGACCTGGT 10895

NCF1C AAATCACAGGTCCCTAGGGGCCTAGTGGCCCATCGGTGACAAAGGGCAGGTGGACCTGGT 10898

NCF1 AAATCACAGGTCCCTAGGGGCCTAGTGGCCCATCGGTGACAAAGGGCAGGTGGACCTGGT 10905 E2F **(myogenin)**

******************* ****************************************

NCF1B GTGGCTGCACCAGAGGGGCCTTCTCATCCTGGGAACTGGGCTAAAAACCAAGCCCAGACT 10955

NCF1C GTGGCTGCACCAGAGGGGCCTTCTCATCCTGGGAACTGGGCTAAAAACCAAGCCCAGACT 10958

NCF1 GTGGCTGCACCAGAGGGGCCTTCTCATCCTGGGAACTGGGCTAAAAACCAAGCCCAGACT 10965

************************************************************

NCF1B GAGGCCCATGCTTTTGTCCCCCCAGCCGCCTCGAGGTCCCTCCTTACCTGCCCCCTGCAC 11015

NCF1C GAGGCCCATGCTTTTGTCCCCCCAGCCGCCTCGAGGTCCCTCCTTACCTGCCCCCTGCAC 11018

NCF1 GAGGCCCATGCTTTTGTCCCCCCAGCCGCCTCGAGGTCCCTCCTTACCTGCCCCCTGCAC 11025 HNF4

************************************************************

NCF1B CCCTACCCCATCTTAACTCTTTTTTTTTTTT--CCAAGATGGAGTCTCGCTCTGTGGCCC 11073

NCF1C CCCTACCCCATCTTAACTCTTTTTTTTTTTTTTCCAAGATGGAGTCTCGCTCTGTTGCCC 11078

NCF1 CCCTACCCCATCTTAACTCTTTTTTTTTTTTT-CCAAGATGGAGTCTCGCTCTGTGGCCC 11084 FoxM1

******************************* ********************** ****

NCF1B AGGCTGGAGTGCAGTGGTGCAATCTCTGCCCCCTGCAACCTCTGCCTACCAGGTTCAAGC 11133

NCF1C AGGCTGGAGTGCAGTGGTGCAATCTCTGCCCCCTGCAACCTCTGCCTACCAGGTTCAAGC 11138

NCF1 AGGCTGGAGTGCAGTGGTGCAATCTCTGTCCCCTGCAACCTCTGCCTACCAGGTTCAAGC 11144

**************************** *******************************

NCF1B TATTCTCCTGCCTCAGCCTCCCGAGTAGCTGGGATTACAGGTGTCCACCACCACACCCAG 11193

NCF1C TATTCTCCTGCCTCAGCCTCCCGAGTAGCTGGGATTACAGGTGTCCACCACCACACCCAG 11198

NCF1 TATTCTCCTGCCTCAGCCTCCCGAGTAGCTGGGATTACAGGTGTCCACCACCACACCCAG 11204 AML1

************************************************************

NCF1B CTAATTTTTGTATTTTTAGTAGAGACGGGTTTCCCCATGTTGGCCAGGTTGGTCTCGAAC 11253

NCF1C CTAATTTTTGTATTTTTAGTAGAGACGGGTTTCCCCATGTTGGCCAGGTTGGTCTCGAAC 11258

NCF1 CTAATTTTTGTATTTTTAGTAGAGACGGGTTTCCCCATGTTGGCCAGGTTGGTCTCGAAC 11264

************************************************************

NCF1B TCCTGACCTCAAGTGATCCACCTGCCTTGGCCTCCCAGAGTTCTGGGATTACAGGCGTGC 11313

NCF1C TCCTGACCTCAAGTGATCCACCTGCCTTGGCCTCCCAGAGTTCTGGGATTACAGGCGTGC 11318

NCF1 TCCTGACCTCAAGTGATCCGCCTGCCTTGGCCTCCCAGAGTTCTGGGATTACAGGCGTGC 11324 Nkx25

******************* ****************************************

NCF1B GTGAGCCACTGCGCCTGGCACCGCACCTTAATTTTGAGGAAGTGTTGGGAAAGCTGGCTC 11373

NCF1C GTGAGCCACTGCGCCTGGCACCGCACCTTAATTTTGAGGAAGTGTTGGGAAAGCTGGCTC 11378

NCF1 GTGAGCCACTGCGCCTGGCACCGCACCTTAATTTTGAGGAAGTGTTGGGAAAGCTGGCTC 11384 ETS, Pea3

************************************************************

NCF1B TGACATCTCTTGTGAGGCAAGCCCAGCTGGGGAAGGTGTTTCCTGCCTGACTTCCTCCCC 11433

NCF1C TGACATCTCTTGTGAGGCAAGCCCAGCTGGGGAAGGTGTTTCCTGCCTGACTTCCTCCCC 11438

NCF1 TGACATCTCTTGTGAGGCAAGCCCAGCTGGGGAAGGTGTTTCCTGCCTGACTTCCTCCCC 11444 ETS, Pea3

************************************************************

NCF1B AGAAGCAATAGCAGGGCTTGCTGTGGCATCCGCTTCCTGCGGGGGCTCAAGGTTGCTCGT 11493

NCF1C AGAAGCAATAGCAGGGCTTGCTGTGGCATCCGCTTCCTGCGGGGGCTCAAGGTTGCTCGT 11498

NCF1 AGAAGCAATAGCAGGGCTTGCTGTGGCATCCGCTTCCTGCGGGGGCTCAAGGTTGCTCGT 11504 CETS168

************************************************************

NCF1B TCCTCAGGCTACATAAAGCAGTAAGGACCCACCCAGGAGGACCCCTCCAGAGGTGTGAGG 11553

NCF1C TCCTCAGGCTACATAAAGCAGTAAGGACCCACCCAGGAGGACCCCTCCAGAGGTGTGAGG 11558

NCF1 TCCTCAGGCTACATAAAGCAGTAAGGACCCACCCAGGAGGACCCCTCCAGAGGTGTGAGG 11564

************************************************************

NCF1B GCGATTTGGTGGACACAGGTAGGGCCAGCGACAGAGCAGAGGGCACACCTCCCAGAGCCC 11613

NCF1C GCGATTTGGTGGACACAGGTAGGGCCAGCGACAGAGCAGAGGGCACACCTCCCAGAGCCC 11618

NCF1 GCGATTTGGTGGACACAGGTAGGGCCAGCGACAGAGCAGAGGGCACACCTCCCAGAGCCC 11624

************************************************************

NCF1B CAAATGCCACAGGCTGAGAAGGTCTAGCTCTAGCTGCATCCAAAGGACCTGTTGAACTCC 11673

NCF1C CAAATGCCACAGGCTGAGAAGGTCTAGCTCTAGCTGCATCCAAAGGACCTGTTGAACTCC 11678

NCF1 CAAATGCCACAGGCTGAGAAGGTCTAGCTCTAGCTGCATCCAAAGGACCTGTTGAACTCC 11684 HNF4

************************************************************

NCF1B TGTCCCGATTGACACTAAAACTGTCCCCTCCTGTATGGTGCCCACACCCTTCAGGGGAAA 11733

NCF1C TGTCCCGATTGACACTAAAACTGTCCCCTCCTGTATGGTGCCCACACCCTTCAGGGGAAA 11738

NCF1 TGTCCCGATTGACACTAAAACTGTCCCCTCCTGTATGGTGCCCACACCCTTCAGGGGAAA 11744

************************************************************

NCF1B CCCCTGGATAAACAGCAGTCACTGCAGGTCTCCAAAAAGACAAGGCCGGAACAGGGAACC 11793

NCF1C CCCCTGGATAAACAGCAGTCACTGCAGGTCTCCAAAAAGACAAGGCCGGAACAGGGAACC 11798

NCF1 CCCCTGGATAAACAGCAGTCACTGCAGGTCTCCAAAAAGACAAGGCCGGAACAGGGAACC 11804

************************************************************

NCF1B TGGGGTGCATGTTCAGGAAGCTGTGGCAAGTGTTGACACCACAGCCCCAAGTTTCTTTTT 11853

NCF1C TGGGGTGCATGTTCAGGAAGCTGTGGCAAGTGTTGACACCACAGCCCCAAGTTTCTTTTT 11858

NCF1 TGGGGTGCATGTTCAGGAAGCTGTGGCAAGTGTTGACACCACAGCCCCAAGTTTCTTTTT 11864 SREBP1, CETS168, AML1

************************************************************

NCF1B CTGGCCCGCGGAAGATGAGCTTCAGTCCCATGGAGGTGACAAGGTTGTCCTCCTGCATGA 11913

NCF1C CTGGCCCGCGGAAGATGAGCTTCAGTCCCATGGAGGTGACAAGGTTGCCCTCCTGCATGA 11918

NCF1 CTGGCCCGCGGAAGATGAGCTTCAGTCCCATGGAGGTGACAAGGTTGTCCTCCTGCATGA 11924

*********************************************** ************

NCF1B TGGGGCTCTGCGGTGCCTCTGCGCCTGGGTAACCCCTCACCGAGAAAGCCACGTCTACAG 11973

NCF1C TGGGGCTCTGCGGTGCCTCTGCGCCTGGGTAACCCCTCACCGAGAAAGCCACGTCTACAG 11978

NCF1 TGGGGCTCTGCGGTGCCTCTGCGCCTGGGTAACCCCTCACCGAGAAAGCCACGTCTACAG 11984

************************************************************

NCF1B GTCCCACCTGTCTGCAGGTGTGCCGCCCCCAACTCCAGGCCTGTGTGACTTAGCGACTGT 12033

NCF1C GTCCCACCTGTCTGCAGGTGTGCCGCCCCCAACTCCAGGCCTGTGTGACTTAGCGACTGT 12038

NCF1 GTCCCACCTGTCTGCAGGTGTGCCGCCCCCAACTCCAGGCCTGTGTGACTTAGCGACTGT 12044 **E2A**

************************************************************

NCF1B CCCAGATCTCTCCTGGGAAGTGGCACAGGGGCCTGGCCCTCAGTGAGTCCCAGAGGGACC 12093

NCF1C CCCAGATCTCTCCTGGGAAGTGGCACAGGGGCCTGACCCTCAGTGAGTCCCAGAGGGACC 12098

NCF1 CCCAGATCTCTCCTGGGAAGTGGCGCAGGGGCCTGGCCCTCAGTGAGTCCCAGAGGGACC 12104

************************ ********** ************************

NCF1B GCACCACCACTGACTTCACTGTGCTGCTTGGAGCTGGGGAGATGTGGAAACGGCCACTCC 12153

NCF1C GCACCACCACTGACTTCACTGTGCTGCTTGGAGCTGGGGAGATGTGGAAACGGCCACTCC 12158

NCF1 GCACCACCACTGACTTCACTGTGCTGCTTGGAGCTGGGGAGATGTGGAAACGGCCACTCC 12164

************************************************************

NCF1B AGACCCAGTGACTGGCTGGTGCTTAGGATGTGATTTCCTCATTACTGAAATTTGTTTCAA 12213

NCF1C AGACCCAGTGACTGGCTGGTGCTTAGGATGTGATTTCCTCATTACTGAAATTTGTTTCAA 12218

NCF1 AGACCCAGTGACTGGCTGGTGCTTAGGATGTGATTTCCTCATTACTGAAATTTGTTTCAA 12224 SRY, Pea3, Nkx25

************************************************************

NCF1B GTGTACAGAAATCACCATCGGCCACGTGCAGTGGCTCACGCCTATAATCCCAGCACTTTG 12273 Myc

NCF1C GTGTACAGAAATCACCATCGGCCACGTGCAGTGGCTCACGCCTATAATCCCAGCACTTTG 12278

NCF1 GTGTACAGAAATCACCATCGGCCACGTGCAGTGGCTCACGCCTATAATCCCAGCACTTTG 12284 USF, Myc

************************************************************

NCF1B GGAGGCCGAGGCGGGCGGATCACCTGAGGTCAGGAGTTCGAGACCAGCCTGGCCAATATG 12333

NCF1C GGAGGCCGAGGCGGGCGGATCACCTGAGGTCAGGAGTTCGAGACCAGCCTGGCCAATATG 12338

NCF1 GGAGGCCGAGGCGGGCGGATCACCTGAGGTCAGGAGTTCGAGACCAGCCTGGCCAATATG 12344

************************************************************

NCF1B GAGAAATCCTGTCTGTACTAAAAATACAAAAATTAGCCGGGTGTGGTAGCGGAAACCTGT 12393

NCF1C GAGAAATCCTGTCTGTACTAAAAATACAAAAATTAGCCGGGTGTGGTAGCGGAAACCTGT 12398

NCF1 GAGAAATCCTGTCTGTACTAAAAATACAAAAATTAGCCGGGTGTGGTAGCGGAAACCTGT 12404 AML1

************************************************************

NCF1B AATCCCAGCTACTCAGGAGGCTGAGGCAGGAGAATTGCTTGAACCCAGGAGGTTGGAGGT 12453

NCF1C AATCCCAGCTACTCAGGAGGCTGAGGCAGGAGAATTGCTTGAACCCAGGAGGTTGGAGGT 12458

NCF1 AATCCCAGCTACTCAGGAGGCTGAGGCAGGAGAATTGCTTGAACCCAGGAGGTTGGAGGT 12464

************************************************************

NCF1B TGCAGTGAGCCGAGACTGCGCCATTGCACTGCAGCCTGGGCAACGGAGTGAGACTTTGCC 12513

NCF1C TGCCGTGAGCCGAGACTGCGCCATTGCACTGCAGCCTGGGCAACGGAGTGAGACTTTGCC 12518

NCF1 TGCAGTGAGCCGAGACTGCGCCATTGCACTGCAGCCTGGGCAACGGAGTGAGACTTTGCC 12524

*** ********************************************************

NCF1B TCAAAAAAAAAAAAAAAAAAAATTTCACCAAAGCCCCTGGGGGTGGTGGTGCACTGCGGA 12573

NCF1C TCAAAAAAAAAAAAAAAAAAAATTTCACCAAAGCCCCTGGGGGTGGTGGTGCACTGCGGA 12578

NCF1 TCAAAAAAAAAAAAAAA---AATTTCACCAAAGCCCCTGGGGGTGGTGGTGCACTGCGGA 12581

***************** ****************************************

NCF1B GTTTATAGCCTTCCTGGGTGGCAGAGGGCAACTGGGCCCCTGAGCACAGAAGTGGGGCTC 12633

NCF1C GTTTATAGCCTTCCTGGGTGGCAGAGGGCAACTGGGCCCCTGAGCACAGAAGTGGGGCTC 12638

NCF1 GTTTATAGCCTTCCTGGGTGGCAGAGGGCAACTGGGCCCCTGAGCACAGAAGTGGGGCTC 12641

************************************************************

NCF1B CCGCGGGGGGTGGGGCTGGCTTGGAAGGTCCCCATTCCTGTGGTGCATATTTGATGAGGG 12693

NCF1C CCGCGGGGGGTGGGGCTGGCTTGGAAGGTCCCCATTCCTGTGGTGCATATTTGATGAGGG 12698

NCF1 CCGCGGGGG-TGGGGCTGGCTTGGAAGGTCCCCATTCCTGTGGTGCATATTTGATGAGGG 12700 TEF1, AML1

********* **************************************************

NCF1B CCCATGTCGTGCCTGGAGCTGGGCCAGAAGCCACAGTGCAGTGCAGTGGGGACAGACCCA 12753 MZF1

NCF1C CCCATGTCGTGCCTGGAGCTGGGCCAGAAGCCACAGTGCAGTGCAGTGGGGACAGACCCA 12758

NCF1 CCCATGTCGTGCCTGGAGCTGGGCCAGAAGCCACAGTGCAGTGCAGTGGGGACAGACCCA 12760

************************************************************

NCF1B GCCTGTCCATGGGCTTGGTTACTGTCAGCCTGGCCGTGTCAGGGAAGGCTTCCAGGCCAC 12813

NCF1C GCCTGTCCATGGGCTTGGTTACTGTCAGCCTGGCCGTGTCAGGGAAGGCTTCCAGGCCAC 12818

NCF1 GCCTGTCCATGGGCTTGGTTACTGTCAGCCTGGCCGTGTCAGGGAAGGCTTCCAGGCCAC 12820

************************************************************

NCF1B CCACACAGCTGCACCACACCCAGACAAGCTCTCCAAGGGACATCATGAGACTGCTCCACT 12873

NCF1C CCACACAGCTGCACCACACCCAGACAAGCTCTCCAAGGGACATCATGAGACTGCTCCACT 12878

NCF1 CCACACAGCTGCACCACACCCAGACAAGCTCTCCAAGGGACATCATGAGACTGCTCCACT 12880 LBP1, AML1

************************************************************

NCF1B GTCAGAGCCCCGGGCCGGTGGTCCCAGCAACTTGGGGTGCCTGCTGTCCTTCATGTGACA 12933

NCF1C GTCAGAGCCCCGGGCCGGTGGTCCCAGCAACTTGGGGTGCCTGCTGTCCTTCATGTGACA 12938

NCF1 GTCAGAGCCCCGGGCCGGTGGTCCCAGCAACTTGGGGTGCCTGCTGTCCTTCATGTGACA 12940 SREBP1, TFE

************************************************************

NCF1B TTTTCTTCATTCAAAGCTCAATTTAAACAGCAAGAAGCCTCCCTGCCTCCTACCTGGCAA 12993

NCF1C TTTTCTTCATTCAAAGCTCAATTTAAACAGCAAGAAGCCTCCCTGCCTCCTACCTGGCAA 12998

NCF1 TTTTCTTCATTCAAAGCTCAATTTAAACAGCAAGAAGCCTCCCTGCCTCCTACCTGGCAA 13000 LEF1

************************************************************

NCF1B TTTCCCTGCCCCGCACCTTGGAGGCAGCCCCTGCGCAGTTTGCATCTGGTGATGGCCAAC 13053

NCF1C TTTCCCTGCCCCGCACCTTGGAGGCAGCCCCTGCGCAGTTTGCATCTGGTGATGGCCAAC 13058

NCF1 TTTCCCTGCCCCGCACCTTGGAGGCAGCCCCTGCGCAGTTTGCATCTGGTGATGGCCAAC 13060 HNF4

************************************************************

NCF1B GTTTGCCACCCTAGTACTTGACCCTATTTAATCCTCACGGCAGCCCATGAAGTGGGTACA 13113

NCF1C GTTTGCCACCCTAGTACTTGACCCTATTTAATCCTCACGGCAGCCCATGAAGTGGGTACA 13118

NCF1 GTTTGCCACCCTAGTACTTGACCCTATTTAATCCTCACGGCAGCCCATGAAGTAGGTACA 13120

***************************************************** ******

NCF1B GTGGTCAGTGCCATTTCACAGATGAAAAGACAGAGGCTCAGGGAGCTTCAGCCACGTGCA 13173

NCF1C GTGGTCAGTGCCATTTCACAGATGAAAAGACAGAGGCTCAGGGAGCTTCAGCCACGTGCA 13178

NCF1 GTGGTCAGTGCCATTTCACAGATGAAAAGACAGAGGCTCAGGGAGCTTCAGCCACGTGCA 13180 USF, Myc

************************************************************

NCF1B CAGCTCAGCCGTCAGCCTGCACGTCTCCCCACGGGGCCTGGGTCTCCGTCTGACTCCCCT 13233

NCF1C CAGCTCAGCCGTCAGCCTGCACGTCTCCCCACGGGGCCTGGGTCTCCGTCTGACTCCCCT 13238

NCF1 CAGCTCAGCCGTCAGCCTGCACGTCTCCCCACGGGGCCTGGGTCTCCGTCTGACTCCCCT 13240

************************************************************

NCF1B GAGTTGAATGCCTGGGGCCTGGAGCCCATTCTCTTGTGTCCCTAGGCCAAGGGCTGGAGA 13293

NCF1C GAGTTGAATGCCTGGGGCCTGGAGCCCATTCTCTTGTGTCCCTAGGCCAAGGGCTGGAGA 13298

NCF1 AAGTCGAATGCCTGGGGCCTGGAGCCCATTCTCTTGTGTCCCTAGGCCAAGGGCTGGAGA 13300

*** *******************************************************

NCF1B TCAGGTCTAGGGGAGAAGGGAATTCCCAGGGGTGTGGTTCAGGTGTGTCCTGGGACAGTC 13353

NCF1C TCAGGTCTAGGGGAGAAGGGAATTCCCAGGGGTGTGGTTCAGGTGTGTCCTGGGACAGTC 13358

NCF1 TCAGGTCTAGGGGAGAAGGGAATTCCCAGGGGTGTGGTTCAGGTGTGTCCTGGGACAGTC 13360 AML1

************************************************************

NCF1B TCCCAAGGGCAGGTCCCTGATTCCCCTCTGCCGAGGCCTCAGGGTCCACCCCTGCCAAGC 13413

NCF1C TCCCAAGGGCAGGTCCCTGATTCCCCTCTGCCGAGGCCTCAGGGTCCACCCCTGCCAAGC 13418

NCF1 TCCCAAGGGCAGGTCCCTGATTCCCCTCTGCCGAGGCCTCAGGGTCCACCCCTGCCAAGC 13420

************************************************************

NCF1B AGAGCCCAGCGCAGGTGATTTGGCTGATAAAGGAAGATGGGTCTCTGGGGAGGCAGCGAG 13473

NCF1C AGAGCCCAGCGCAGGTGATTTGGCTGATAAAGGAAGATGGGTCTCTGGGGAGGCAGCGAG 13478

NCF1 AGAGCCCAGCGCAGGTGATTTGGCTGATAAAGGAAGATGGGTCTCTGGGGAGGCAGCAAG 13480

********************************************************* **

NCF1B TGGCCCACTTTCTTCTTTGCTTTTTTTTTTTGAGATGGAGTCTCACTCACTCTGTCTCCC 13533

NCF1C TGGCCCACTTTCTTCTTTGCTTTTTTTTTTTGAGATGGAGTCTCACTCACTCTGTCTCCC 13538

NCF1 TGGCCCACTTTCTTCTTTGCTTTTTTTTTTTGAGATGGAGTCTCACTCACTCTGTCTCCC 13540 FoxM1

************************************************************

NCF1B AGGCTGGAATGCAGTGGCACGATCTCGGGTCACTGCAACCTCCGCCTCCCGGGTTCAAGA 13593

NCF1C AGGCTGGAATGCAGTGGCACGATCTCGGGTCACTGCAACCTCCGCCTCCCGGGTTCAAGA 13598

NCF1 AGGCTGGAATGCAGTGGCACGATCTCGGGTCACTGCAACCTCCGCCTCCCGGGTTCAAGA 13600 TEF1

************************************************************

NCF1B GAGTCTCCTGCCTCGGCCTCCTGAGTAGCTGGGATTACAAGCACCTGCCACCATGCCCAG 13653 **E2A (myogenin)**

NCF1C GAGTCTCCTGCCTCGGCCTCCTGAGTAGCTGGGATTACAAGCACCTGCCACCATGCCCAG 13658

NCF1 GAGTCTCCTGCCTCGGCCTCCTGAGTAGCTGGGATTACAAGCACCTGCCACCATGCCCAG 13660

************************************************************

NCF1B CTAATTTTTGTATTTTTTGTAGAGATGGGGTTCACCATGTTGGCCAAGGCTGGAGTGGCC 13713

NCF1C CTAATTTTTGTATTTTTTCTAGAGATGGGGTTCACCATGTTGGCCAAGGCTGGAGTGGCC 13718

NCF1 CTAATTTTTGTATTTTTTGTAGAGATGGGGTTCACCATGTTGGCCAAGGCTGGAGTGGCC 13720

****************** *****************************************

NCF1B AGCGTTTCTGCCTATTGACACCTGCCTGCCAATACAGGGTCGGTCACACAGAGCCAGCGT 13773

NCF1C AGCGTTTCTGCCTATTGACACCTGCCTGCCAATACAGGGTCGGTCACACAGAGCCAGCGT 13778

NCF1 AGCGTTTCTGCCTATTGACACCTGCCTGCCAATACAGGGTCGGTCACACAGAGCCAGCGT 13780 E2A (myogenin), CREB

************************************************************

NCF1B CATAGGTACCTTGGGGCCCATCCTGCTGCCCTAGGCAGGAGGGAGAGCTGGTCCTGTGGG 13833

NCF1C CATAGGTACCTTGGGGCCCATCCTGCTGCCCTAGGCAGGAGGGAGAGCTGGTCCTGTGGG 13838

NCF1 CATAGGTACCTTGGGGCCCATCCTGCTGCCCTAGGCAGGAGGGAGAGCTGGTCCTGTGGG 13840

************************************************************

NCF1B CTGGCCCAGGAGACGGTCACCCAGGGCTGTCCAAGGGGATGGATCCTGGCTGTCACTCGC 13893

NCF1C CTGGCCCAGGAGACGGTCACCCAGGGCTGTCCAAGGGGATGGATCCTGGCTGTCACTCGC 13898

NCF1 CTGGCCCAGGAGACGGTCACCCAGGGCTGTCCAAGGGGATGGATCCTGGCTGTCACTCGC 13900

************************************************************

NCF1B TCAAGAAGAGGCAGCAGGAGAGGCTTAGGTTAGATCTGGGAAAGAACTGTCCCACCCAAA 13953

NCF1C TCAAGAAGAGGCAGCAGGAGAGGCTTAGGTTAGATCTGGGAAAGAACTGTCCCACCCAAA 13958

NCF1 TCAAGAAGAGGCAGCAGGAGAGGCTTAGGTTAGATCTGGGAAAGAACTGTCCCACCCAAA 13960

************************************************************

NCF1B GGCTGATTTGCTCCAGTCCTGGGAACTGCTAGATTCCCTCTCCTGATAAAATGTCCCAGA 14013

NCF1C GGCTGATTTGCTCCAGTCCTGGGAACTGCTAGATTCCCTCTCCTGATAAAATGTCCCAGA 14018

NCF1 GGCTGATTTGCTCCAGTCCTGGGAACTGCTAGATTCCCTCTCCTGATAAAATGTCCCAGA 14020

************************************************************

NCF1B GGGCACTGCGTGGGTCCCATGCCCTGATGCCAAGTCGCCTTTCTCCTGATATGGTACCCA 14073

NCF1C GGGCACTGCGTGGGTCCCATGCCCTGATGCCAAGTCGCCTTTCTCCTGATATGGTACCCA 14078

NCF1 GGGCACTGCGTGGGTCCCATGCCCTGACGCCAAGTCGCCTTTCTCCTGATATGGTACCCA 14080

*************************** ********************************

NCF1B CTGGCCTGGCCTTGGGGATTTCCTGGTTAAAGAAATAGCGAGCTCAGCCCATCTGGGGAA 14133

NCF1C CTGGCCTGGCCTTGGGGATTTCCTGGTTAAAGAAATAGCGAGCTCAGCCCATCTGGGGAA 14138

NCF1 CTGGCCTGGCCTTGGGGATTTCCTGGTTAAAGAAATAGCGAGCTCAGCCCATCTGGGGAA 14140 CREL

************************************************************

NCF1B AGCCAAGTGGCCTGAGGGCGGAGAAGGTGACATTTGGAAAAAGGCCAGGACTGGCAGCTC 14193

NCF1C AGCCAAGTGGCCTGAGGGCGGAGAAGGTGACATTTGGAAAAAGGCCAGGACTGGCAGCTC 14198

NCF1 AGCCAAGTGGCCTGAGGGCGGAGAAGGTGACATTTGGAAAAAGGCCAGGACTGGCAGCTC 14200

************************************************************

NCF1B AGACCTGGAAGCCCCCACTCCCTGCCAGCCTGGCTTGAGGTGAGAGCCTTCCTCATGAGC 14253

NCF1C AGACCTGGAAGCCCCCACTCCCTGCCAGCCTGGCTTGAGGTGAGAGCCTTCCTCATGAGC 14258

NCF1 AGACCTGGAAGCCCCCACTCCCTGCCAGCCTGGCTTGAGGTGAGAGCCTTCCTCATGAGC 14260

************************************************************

NCF1B CCCCTACCCCCACTCATTCATTCATTCATTCATTCACATTGTTCAAGGGCAAGGCATGAG 14313

NCF1C CCCCTACCCCCACTCATTCATTCATTCATTCATTCACATTGTTCAAGGGCAAGGCATGAG 14318

NCF1 CCCCTACCCCCACTCATTCATTCATTCATTCATTCACATTGTTCAAGGGCAAGGCATGAG 14320

************************************************************

NCF1B AGTGCAACAGTTAAATCATGGGCCTGGGTCCAGACTGCCCAAGTTCAATCGCGGTTCTAG 14373

NCF1C AGTGCAACAGTTAAATCATGGGCCTGGGTCCAGACTGCCCAAGTTCAATCGCGGTTCTAG 14378

NCF1 AGTACAACAGTTAAATCATGGGCCTGGGTCCAGACTGCCCAAGTTCAATCGCGGTTCTAG 14380 HNF4

*** ********************************************************

NCF1B CCTGGCGCGATGGCTCATGCCTGTAATCCCAGCACTTTGGGAGGCCGAGGCAGGCAGATC 14433

NCF1C CCTGGCGCGATGGCTCATGCCTGTAATCCCAGCACTTTGGGAGGCCGAGGCAGGCAGATC 14438

NCF1 CCTGGCGCGATGGCTCATGCCTGTAATCCCAGCACTTTGGGAGGCTGAGGCAGGCAGATC 14440 E2F

********************************************* **************

NCF1B ACTTGAGGTCAGGAGTTCGAGACCAGCTTGGGCAACATGGTGAAACCCCATCTCTACAAA 14493

NCF1C ACTTGAGGTCAGGAGTTCGAGACCAGCCTGGGCAACATGGTGAAACCCCATCTCTACAAA 14498

NCF1 ACTTGAGGTCAGGAGTTCGAGACCAGCCTGGGCAACATGGTGAAACCCCATCTCTACAAA 14500 Nkx25

*************************** ********************************

NCF1B AAATACAAAAATTAGCCGGGCGTGGTGGCACGAGCCTGTAATCCCAGCTACTCGGGAGGC 14553

NCF1C AAATACAAAAATTAGCCGGGCGTGGTGGCACGAGCCTGTAATCCCAGCTACTCGGGAGGC 14558

NCF1 AAATACAAAAATTAGCCGGGCGTGGTGGCACGAGCCTGTAATCCCAGCTACTCGGGAGGC 14560

************************************************************

NCF1B TGGCACAGAATTTCTTGAACCAGGGAGGCGGAGGTTACAGTGAGGCAGGATTGCGCCACT 14613

NCF1C TGGCACAGAATTTCTTGAACCAGGGAGGCGGAGGTTACAGTGAGGCAGGATTGCGCCACT 14618

NCF1 TGGCACAGAATTTCTTGAACCAGGGAGGCGGAGGTTACAGTGAGGCAGGATTGCGCCACT 14620

************************************************************

NCF1B GCACTCCAGCCTGGGTGACACAGCAAGACTCTGTCTCAAAACAAAAACAAAACAAATCCC 14673

NCF1C GCACTCCAGCCTGGGTGACACAGCAAGACTCTGTCTCAAAACAAAAACAAAACAAATCCC 14678

NCF1 GCACTCCAGCCTGAGTGACACAGCAAGACTCTGTCTCAAAACAAAAACAAAACAAATCCC 14680 SRY

************* **********************************************

NCF1B AGTTCTGCATATACCAGCTGTGGCAATCTGTTTTCCTCTCTGTTTTCCCATCTGTGAAGT 14733

NCF1C AGTTCTGCATATACCAGCTGTGGCAATCTGTTTTCCTCTCTGTTTTCCCATCTGTGAAGT 14738

NCF1 AGTTCTGCATATACCAGCTGTGGCAATCTGTTTTCCTCTCTGTTTTCCCATCTGTGAAGT 14740 AP4

************************************************************

NCF1B GGGGAAAATTGTACCCACTCCCTGACAGCCTCCTTGTGAGGAGAAGATACGGTCATAAAT 14793

NCF1C GGGGAAAATTGTACCCACTCCCTGACAGCCTCCTTGTGAGGAGAAGATACGGTCATAAAT 14798

NCF1 GGGGAAAATTGTACCCACTCCCTGACAGCCTCCTTGTGAGGAGAAGATACGGTCATAAAT 14800 MZF1

************************************************************

NCF1B AGAAAGCACCTAGAATAGTGCCTGACGTAGGGCAGGGCGGCGGGGTGCGGGGGGCCCTTC 14853

NCF1C AGAAAGCACCTAGAATAGTGCCTGACGTAGGGCAGGGCGGCGGGGTGCGGGGGGCCCTTC 14858

NCF1 AGAAAGCACCTAGAATAGTGCCTGACGTAGGGCAGGGCGGCGGGGTGCGGGGGGCCCTTC 14860 ETF

************************************************************

NCF1B CTATTAGCACTCGGGACGTGGGGGAATTCTTGGGGCCCTGGTATTGTTCTAACACCCAGT 14913

NCF1C CTATTAGCACTCGGGACGTGGGGGAATTCTTGGGGCCCTGGTATTGTTCTAACACCCAGT 14918

NCF1 CTATTAGCACTCGGGACGTGGGGGAATTCTTGGGGCCCTGGTATTGTTCTAACACCCAGT 14920

************************************************************

NCF1B GATGGTTCACCAGCTGTCTCCTTTATAATAATTAGGTAAGAGATGTGGTTTTCTACATAT 14973

NCF1C GATGGTTCACCAGCTGTCTCCTTTATAATAATTAGGTAAGAGATGTGGTTTTCTACATAT 14978

NCF1 GATGGTTCACCAGCTGTCTCCTTTATAATAATTAGGTAAGAGATGTGGTTTTCTACATAT 14980 HoxA4, AML1

************************************************************

NCF1B TTCACAATACAAACATTTTTAGAAATTCTATTTCCTTCCCCTGAAAAAACTCTCTTATCT 15033

NCF1C TTCACAATACAAACATTTTTAGAAATTCTATTTCCTTCCCCTGAAAAAACTCTCTTATCT 15038

NCF1 TTCACAATACAAACATTTTTAGAAATTCTATTTCCTTCCCCTGAAAAAACTCTCTTATCT 15040 Stat5A,GATA3(GATA2),GATA

************************************************************

NCF1B CTTTATTACTTCCTTCCTTAACTTTATTTTATTTTATTTTATTTATTTATTTTTTGAAAC 15093 Pea3

NCF1C CTTTATTACTTCCTTCCTTAACTTTATTTTATTTTATTTTATTTATTTATTTTTTGAAAC 15098

NCF1 CTTTATTACTTCCTTCCTTAACTTTATTTTATTTTATTTTATTTATTTATTTTTTGAAAC 15100

************************************************************

NCF1B CAAGTCTACTCTGTTACCCAGCCTGGAGTGCAGTGGTGTGATCTCGGCTCACTGCAACCT 15153

NCF1C CAAGTCTACTCTGTTACCCAGCCTGGAGTGCAGTGGTGTGATCTCGGCTCACTGCAACCT 15158

NCF1 CAAGTCTACTCTGTTACCCAGCCTGGAGTGCAGTGGTGTGATCTCGGCTCACTGCAACCT 15160

************************************************************

NCF1B CTGCCTCCCAGGTTCAAGCGATTCTTCTGCCTCAGCCTCCCAAGTAGCTGGGATTATAGG 15213

NCF1C CTGCCTCCCAGGTTCAAGCGATTCTTCTGCCTCAGCCTCCCAAGTAGCTGGGATTATAGG 15218

NCF1 CTGCCTCCCAGGTTCAAGCGATTCTTCTGCCTCAGCCTCCCAAGTAGCTGGGATTATAGG 15220

************************************************************

NCF1B CTTGCAATGCCATGCCTGGCTAATTTTTTTATTTTTAGTAGAGACCCGGTTTCACCGTGT 15273

NCF1C CTTGCAATGCCATGCCTGGCTAATTTTTTTATTTTTAGTAGAGACCCGGTTTCACCGTGT 15278

NCF1 CTTGCAATGCCATGCCTGGCTAATTTTTTTATTTTTAGTAGAGACCCGGTTTCACCGTGT 15280

************************************************************

NCF1B TGGCCAAGCTGGTCCCGAACTCCTGACCTCAGATGACCCACCCACCTTGGCCTCCCAAAG 15333

NCF1C TGGCCAAGCTGGTCCCGAACTCCTGACCTCAGATGACCCACCCACCTTGGCCTCCCAAAG 15338

NCF1 TGGCCAAGCTGGTCCCGAACTCCTGACCTCAGATGACCCACTCACCTTGGCCTCCCAAAG 15340

***************************************** ******************

NCF1B TGCTGGAATTACAGGCATGAGCCACTGCACCTATCTTTTTTTTTTTAATTAAAAAAATTA 15393

NCF1C TGCTGGAATTACAGGCATGAGCCACTGCACCTATCTTTTTTTTTTTAATTAAAAAAATTA 15398

NCF1 TGCTGGAATTACAGGCATGAGCCACTGCACCTATCTTTTTTTTTTTAATTAAAAAAATTA 15400

************************************************************

NCF1B TTTGGTACCTTGTTTCATCCATGCATTAAATTAAATCCTGGCCAGACACAGTGGCTTATG 15453

NCF1C TTTGGTACCTTGTTTCATCCATGCATTAAATTAAATCCTGGCCAGACACAGTGGCTTATG 15458

NCF1 TTTGGTACCTTGTTTCATCCATGCATTAAATTAAATCCTGGCCAGACACAGTGGCTTATG 15460

************************************************************

NCF1B CCTGTAATCCCAGCATTTTGGGAGGCTGAGGTGGGAGGACCACTTGATACTGGAGCTTGA 15513

NCF1C CCTGTAATCCCAGCATTTTGGGAGGCTGAGGTGGGAGGACCACTTGATACTGGAGCTTGA 15518

NCF1 CCTGTAATCCCAGCATTTTGGGAGGCTGAGGTGGGAGGACCACTTGATACTGGAGCTTGA 15520 Nkx25

************************************************************

NCF1B GACCAGGCCGAGCAGCATCTCGAGACCCCGTCTCTACAAAAAAAAAAATAA-TAATAATA 15572

NCF1C GACCAGGCCGAGGAGCATCTCGAGACCCCGTCTCTACAAAAAAAAAAATAA-TAATAATA 15577

NCF1 GACCAGGCCGAGCAGCATCTCGAGACCCCGTCTCTACAAAAAAAAAAAAAAATAATAATA 15580

************ *********************************** ** ********

NCF1B ATAATAATAAATAAAAAGTGGAAAAAATCCTATGTCATCCTGAAAAAAGGCTGTAAGCCT 15632

NCF1C ATAATAATAAATAAAAAGTGGAAAAAATCCTATGTCATCCTGAAAAAAGGCTGTAAGCCT 15637

NCF1 ATAATAATAAATAAAAAGTGGAAAAAATCCTATGTCATCCTGAAAAAAGGCTGTAAGCCT 15640 CIZ

************************************************************

NCF1B GCTTACAGAGGTCATTACAAGGTCAAACTCAAGTTCAGAGCGCTTCCTGCCTCTGCTCAT 15692

NCF1C GCTTACAGAGGTCATTACAAGGTCAAACTCAAGTTCAGAGCGCTTCCTGCCTCTGCTCAT 15697

NCF1 GCTTACAGAGGTCATTACAAGGTCAAACTCAAGTTCAGAGCGCTTCCTGCCTCTGCTCAT 15700 SF1, HNF4, CETS168

************************************************************

NCF1B CCAACAAACTTGCTGGATACCTCCTGTCTGCAGAGCACTTTGAGGGAACATAACAGGGTC 15752

NCF1C CCAACAAACTTGCTGGATACCTCCTGTCTGCAGAGCACTTTGAGGGAACATAACAGGGTC 15757

NCF1 CCAACAAACTTGCTGGATACCTCCTGTCTGCAGAGCACTTTGAGGGAACATAACAGGGTC 15760 LEF1

************************************************************

NCF1B TTGGGAGGCCACAGGAGGAGAGTTGAAAGATCACAGCCAGGGGCTCAGGGTGTCCACAGG 15812

NCF1C TTGGGAGGCCACAGGAGGAGAGTTGAAAGATCACAGCCAGGGGCTCAGGGTGTCCACAGG 15817

NCF1 TTGGGAGGCCACAGGAGGAGAGTTGAAAGATCACAGCCAGGGGCTCAGGGTGTCCACAGG 15820

************************************************************

NCF1B ACAAGTACCCTTGGCCAGGCAGTTACGCAAGTGTGGAAAGACCGCTAGAGGAAGGGAAGG 15872

NCF1C ACAAGTACCCTTGGCCAGGCAGTTACGCAAGTGTGGAAAGACCGCTAGAGGAAGGGAAGG 15877

NCF1 ACAAGTACCCTTGGCCAGGCAGTTACGCAAGTGTGGAAAGACCGCTAGAGGAAGGGAAGG 15880 Pea3

************************************************************

NCF1B AAGTGCCGAGAGCCCACAAAATTCTCTGCTTACAACCAGCCCCACTAGAACCTTCCTCTG 15932

NCF1C AAGTGCCGAGAGCCCACAAAATTCTCTGCTTACAACCAGCCCCACTAGAACCTTCCTCTG 15937

NCF1 AAGTGCCGAGAGCCCACAAAATTCTCTGCTTACAACCAGCCCCACTAGAACCTTCCTCTG 15940

************************************************************

NCF1B CCCTGCCTCGACATGCCCAGGAGAGCACCGCTGCAGGTCTGGCCTCTGTGCTGAGCCTTT 15992

NCF1C CCCTGCCTCGACATGCCCAGGAGAGCACCGCTGCAGGTCTGGCCTCTGTGCTGAGCCTTT 15997

NCF1 CCCTGCCTCGACATGCCCAGGAGAGCACCGCTGCAGGTCTGGCCTCTGTGCTGAGCCTTT 16000

************************************************************

NCF1B TTTTTTTTTTTTTTCCTGAGACAGAATCTCACTCTGCTTCCCAGGCTGGAGTGCAGTGGC 16052

NCF1C TTTTTTTTTTTTTTCCTGAGACAGAATCTCACTCTGCTTCCCAGGCTGGAGTGCAGTGGC 16057

NCF1 TTTTTTTTTTTTT-CCTGAGACAGAATCTCACTCTGCTTCCCAGGCTGGAGTGCAGTGGC 16059

************* **********************************************

NCF1B AGGATCTCGGCTCACTGCAACCTCCACCTCCCTGGTTCAAGTGATTCTCCTGTCTTACCC 16112

NCF1C AGGATCTCGGCTCACTGCAACCTCCACCTCCCTGGTTCAAGTGATTCTCCTGTCTTACCC 16117

NCF1 AGGATCTCGGCTCACTGCAACCTCCACCTCCCTGGTTCAAGTGATTCTCCTGTCTTACCC 16119 Nkx25

************************************************************

NCF1B TCCGGAGTAGCTGGGATTACAGGTGTGTGCCACCATGCCCAGCTAATTTTTGTATTTTTA 16172

NCF1C TCCGGAGTAGCTGGGATTACAGGTGTGTGCCACCATGCCCAGCTAATTTTTGTATTTTTA 16177

NCF1 TCCGGAGTAGCTGGGATTACAGGTGTGTGCCACCATGCCCAGCTAATTTTTGTATTTTTA 16179

************************************************************

NCF1B GTGGAGACTGGGTTTCACCATGTTGGGCCAGCTGGTCTTGAACTCCTGACCTCAGGTGAT 16232

NCF1C GTGGAGACTGGGTTTCACCATGTTGGGCCAGCTGGTCTTGAACTCCTGACCTCAGGTGAT 16237

NCF1 GTGGAGACTGGGTTTCACCATGTTGGGCCAGCTGGTCTTGAACTCCTGACCTCAGGTGAT 16239 HEB, HNF4

************************************************************

NCF1B CCGCCCACCGTAGCCTCCCAAAGTTCTGGGATTAGAGCTATGAGCCACCATGCCTGGCTA 16292

NCF1C CCGCCCACCGTAGCCTCCCAAAGTTCTGGGATTAGAGCTATGAGCCACCATGCCTGGCTA 16297

NCF1 CCGCCCACCGTAGCCTCCCAAAGTTCTGGGATTAGAGCTATGAGCCACCATGCCTGGCTA 16299

************************************************************

NCF1B CCATGCTGGGCCTTTCGAGGAGGCATTTGACAGGGAAGATGAGAGACAAATTGAGTGTCA 16352

NCF1C CCGTGCTGGGCCTTTCGAGGAGGCATTTGACAGGGAAGATGAGAGACAAATTGAGTGTCA 16357

NCF1 CCGTGCTGGGCCTTTCGAGGAGGCATTTGACAGGGAAGATGAGAGACAAATTGAGTGTCA 16359

** *********************************************************

NCF1B GGGAAGGGGTGTTGATAGAAAAATTACAGGAGAGCACACAACTTTCAGCGGGTGAGCCCA 16412

NCF1C GGGAAGGGGTGTTGATAGAAAAATTACAGGAGAGCACACAACTTTCAGCGGGTGAGCCCA 16417

NCF1 GGGAAGGGGTGTTGATAGAAAAATTACAGGAGAGCACACAACTTTCAGCGGGTGAGCCCA 16419

************************************************************

NCF1B GTGCCTGAGCTGCGGGACCACCCTACCAATGACCTTGAACTTATCTGACTGCAGCCTTGA 16472

NCF1C GTGCCTGAGCTGCGGGACCACCCTACCAATGACCTTGAACTTATCTGACTGCAGCCTTGA 16477

NCF1 GTGCCTGAGCTGCGGGACCACCCTACCAATGACCTTGAACTTATCTGACTGCAGCCTTGA 16479 SF1, GATA

************************************************************

NCF1B ACTCCTGAGCTCAAGGAGTCCTTCTGCCTCAGCCTCCTCCCAAGTAGCTGGGACTACTGG 16532

NCF1C ACTCCTGAGCTCAAGGAGTCCTTCTGCCTCAGCCTCCTCCCAAGTAGCTGGGACTACTGG 16537

NCF1 ACTCCTGAGCTCAAGGAGTCCTTCTGCCTCAGCCTCCTCCCAAGTAGCTGGGACTACTGG 16539

************************************************************

NCF1B CACATGCCACCATGCCCAGCTAATTATTTTATTTATTTTATTTTATTTTATTTTATTTTA 16592

NCF1C CACATGCCACCATGCCCAGCTAATTATTTTATTTATTTTATTTTATTTTATTTTATTTTA 16597

NCF1 CACATGCCACCATGCCCAGCTAATTATTTTATTTATTTTATTTTATTTTATTTTATTTTA 16599 HoxA4

************************************************************

NCF1B TTTTATTTTGAGATGGAGTTTTGTCCTTGTTGCCCAGGCTGGAGTGCAATGGTGCAATCT 16652

NCF1C TTTT-----GAGATGGAGTTTTGTCCTTGTTGCCCAGGCTGGAGTGCAATGGTGCAATCT 16652

NCF1 TTTTATTTTGAGATGGAGTTTTGTCCTTGTTGCCCAGGCTGGAGTGCAATGGTGCAATCT 16659 FoxM1

**** ***************************************************

NCF1B CAGCTCGCCGCAACTTCTGCCTCCCAGGTTCAAGCGAATTCTCCTGCCTCAGCCTCCTGA 16712

NCF1C CAGCTCGCCGCAACTTCTGCCTCCCAGGTTCAAGCGAATTCTCCTGCCTCAGCCTCCTGA 16712

NCF1 CAGCTCGCCGCAACTTCTGCCTCCCAGGTGCAAGCGAATTCTCCTGCCTCAGCCTCCTGA 16719

***************************** ******************************

NCF1B GTAGCTGGGATTACAGGCATGTGCCACCACGCCTGGCTAATTTTGTATTTTTAGTAGAGA 16772

NCF1C GTAGCTGGGATTACAGGCATGTGCCACCACGCCTGGCTAATTTTGTATTTTTAGTAGAGA 16772

NCF1 GTAGCTGGGATTACAGGCATGTGCCACCACGCCTGGCTAATTTTGTATTTTTAGTAGAGA 16779

************************************************************

NCF1B CGGGGTTTCACCATGTTGGCCACGCTGGTCTCGAACCCCTGACCTCAGGTGATCCACCTG 16832

NCF1C CGGGGTTTCACCATGTTGGCCACGCTGGTCTCGAACCCCTGACCTCAGGTGATCCACCTG 16832

NCF1 CGGGGTTTCACCATGTTGGCCACGCTGGTCTCGAACCCCTGACCTCAGGTGATCCACCTG 16839 E2A **(myogenin)**

************************************************************

NCF1B CCTCGGCCTCCCAAAGTACTGAGATTACAGGCATGAGCCACCGCACCTGGCCCCACTTGT 16892

NCF1C CCTCGGCCTCCCAAAGTACTGAGATTACAGGCATGAGCCACCGCACCTGGCCCCACTTGT 16892

NCF1 CCTCGGCCTCCCAAAGTACTGAGATTACAGGCATGAGCCACCGCACCTGGCCCCACTTGT 16899

************************************************************

NCF1B GGAACTAGCATCTATCTGGAGAGGAGGCAAACATCGCCCACCACCTCCCGCTCTCTCCTG 16952

NCF1C GGAACTAGCATCTATCTGGAGAGGAGGCAAACATCGCCCACCACCTCCCGCTCTCTCCTG 16952

NCF1 GGAACTAGCATCTATCTGGAGAGGAGGCAAACATCGCCCACCACCTCCCGCTCTCTCCTG 16959

************************************************************

NCF1B TCACCACTGTCCCCACCATCATTCCAGAGGTCACCCTGGCTTCCAACACCACAGCCTGGC 17012

NCF1C TCACCACTGTCCCCACCATCATTCCAGAGGTCACCCTGGCTTCCAACACCACAGCCTGGC 17012

NCF1 TCACCACTGTCCCCACCATCATTCCAGAGGTCACCCTGGCTTCCAACACCACAGCCTGGC 17019 TEF1, AML1

************************************************************

NCF1B TTGGGCAGTTTTCAAGCCTCGTATAAATGACATCCTCCAGAACATGTGCTCTGTGCCTGC 17072

NCF1C TTGGGCAGTTTTCAAGCCTCGTATAAATGACATCCTCCAGAACATGTGCTCTGTGCCTGC 17072

NCF1 TTGGGCAGTTTTCAAGCCTCGTATAAATGACATCCTCCAGAACATGTGCTCTGTGCCTGC 17079 Pea3

************************************************************

NCF1B CTTCCTTCCGTCAGTGATGTATCTGGAAGATTCCACTGTGTCGCCCTGTGGGACAGGTCC 17132

NCF1C CTTCCTTCCGTCAGTGATGTATCTGGAAGATTCCACTGTGTCGCCCTGTGGGACAGGTCC 17132

NCF1 CTTCCTTCCGTCAGTGATGTATCTGGAAGATTCCACTGTGTCGCCCTGTGGGACAGGTCC 17139

************************************************************

NCF1B TTGTCATTGCTGAGTAGATCCTGTTGCAAATGCCTATCTCTCTTCATGGAAAGATCCAAG 17192

NCF1C TTGTCATTGCTGAGTAGATCCTGTTGCAAATGCCTATCTCTCTTCATGGAAAGATCCAAG 17192

NCF1 TTGTCATTGCTGAGTAGATCCTGTTGCAAATGCCTATCTCTCTTCATGGAAAGATCCAAG 17199

************************************************************

NCF1B ATACACAGATGGAAATCATCATAGGAAGGGCTGGCAAGGCCGTTCACACCCAGGGCTGGG 17252

NCF1C ATACACAGATGGAAATCATCATAGGAAGGGCTGGCAAGGCCGTTCACACCCAGGGCTGGG 17252

NCF1 ATATACAGATGGAAATCATCATAGGAAGGGCTGGCAAGGCCGTTCACACCCAGGGCTGGG 17259

*** ********************************************************

NCF1B GACCTCAGGGTGGAGGTGGGGGACAGTAAGGACCAGAAGGAGCAGGTGCCGGCGGGTGAT 17312

NCF1C GACCTCAGGGTGGAGGTGGGGGACAGTAAGGACCAGAAGGAGCAGGTGCCGGCGGGTGAT 17312

NCF1 GACCTCAGGGTGGAGGTGGGGGACAGTAAGGACCAGAAGGAGCAGGTGCCGGCGGGTGAT 17319

************************************************************

NCF1B GTGAGCTTTCTTCTCTATAGAGAAGTGAAGGCCGGGTGCAGTGGCTCACTCCTGTAATCC 17372

NCF1C GTGAGCTTTCTTCTCTATAGAGAAGTGAAGGCCGGGTGCAGTGGCTCACTCCTGTAATCC 17372

NCF1 GTGAGCTTTCTTCTCTATAGAGAAGTGAAGGCCGGGTGCAGTGGCTCACTCCTGTAATCC 17379 IRF1

************************************************************

NCF1B CAGCGCTTTGGGAGGTCGAGGCGGGCAGATCACTTGAGGTCAGGAGTTCGAGACCAGCCT 17432

NCF1C CAGCGCTTTGGGAGGTCGAGGCGGGCAGATCACTTGAGGTCAGGAGTTCGAGACCAGCCT 17432

NCF1 CAGCGCTTTGGGAGGTCGAGGCGGGCAGATCACTTGAGGTCAGGAGTTCGAGACCAGCCT 17439 Nkx25

************************************************************

NCF1B GGGCAATTTGGTGAAACCCCATCACTATAAAAATACAAAAAATTAGCCGGACGTGGTGGT 17492

NCF1C GGGCAATTTGGTGAAACCCCATCACTATAAAAATACAAAAAATTAGCCGGACGTGGTGGT 17492

NCF1 GGGCAATTTGGTGAAACCCCATCACTATAAAAATACAAAAAATTAGCCGGACGTGGTGGT 17499

************************************************************

NCF1B GCACGCCTGTAATCCCAGCTATTTGGGAGGCTGAGGCAGGAGAATTGCTTGTACCCGGAA 17552

NCF1C GCACGCCTGTAATCCCAGCTATTTGGGAGGCTGAGGCAGGAGAATTGCTTGTACCCGGAA 17552

NCF1 GCACGCCTGTAATCCCAGCTATTTGGGAGGCTGAGGCAGGAGAATTGCTTGTACCCGGAA 17559

************************************************************

NCF1B GGTGAAGGTTGCAGTGAGCCGAGATCATGCCACTGCATACCAGCCTGGGGGACAGAAAGA 17612

NCF1C GGTGAAGGTTGCAGTGAGCCGAGATCATGCCACTGCATACCAGCCTGGGGGACAGAAAGA 17612

NCF1 GGTGAAGGTTGCAGTGAGCCGAGATCATGCCACTGCATACCAGCCTGGGGGACAGAAAGA 17619

************************************************************

NCF1B GACTCTGTCTCAAAAAAAAAAAAAAGAAAAAAAGAAGTGAAGCACTTGCCAAGCAAATCT 17672

NCF1C GACTCTGTCTCAAAAAAAAAAAAAAGAAAAAAAGAAGTGAAGCACTTGCCAAGCAAATCT 17672

NCF1 GACTCTGTCTCAAAAAAAAAAAAAAGAAAAAAAGAAGTGAAGCACTTGCCAAGCAAATCT 17679 IRF1

************************************************************

NCF1B TTCAGAGCAGGTGGAGTGGACCCTACACCTCTTGGATAATAAATGCACTGGATAATAAAA 17732

NCF1C TTCAGAGCAGGTGGAGTGGACCCTACACCTCTTGGATAATAAATGCACTGGATAATAAAA 17732

NCF1 TTCAGAGCAGGTGGAGTGGACCCTACACCTCTTGGATAATAAATGCACTGGATAATAAAA 17739

************************************************************

NCF1B GCAGGAACAGGCCAGGTGTGGTGGCATGTGCCTGTAGTCCCAACCTACTGGGGAGGCCAA 17792

NCF1C GCAGGAACAGGCCAGGTGTGGTGGCATGTGCCTGTAGTCCCAACCTACTGGGGAGGCCAA 17792

NCF1 GCAGGAACAGGCCAGGTGCGGTGGCATGTGCCTGTAGTCCCAACCTACTGGGGAGGCCAA 17799

****************** *****************************************

NCF1B GGCAGGAGGACTGCTTGAGCCCAGGAGTTGGAGGCTGCAGTGAGTTATGACCAGGCAACT 17852

NCF1C GGCAGGAGGACTGCTTGAGCCCAGGAGTTGGAGGCTGCAGTGAGTTATGACCAGGCAACT 17852

NCF1 GGCAGGAGGACTGCTTGAGCCCAGGAGTTGGAGGCTGCAGTGAGTTATGACCAGGCAACT 17859

************************************************************

NCF1B GCACTCCAGCCTGGGTGACAGATAGAGACCCTGTCTTTAAAAAAAAAAAAAAAAAAAAAA 17912

NCF1C GCACTCCAGCCTGGGTGACAGATAGAGACCCTGTCTTTAAAAAAAAAAAAAAAAAAAAAA 17912

NCF1 GCACTCCAGCCTGGGTGACAGATAGAGACCCTGTCTTTAAAAAAAAAAAAAAAAAAAAAA 17919

************************************************************

NCF1B AAAAGGGCCAGGCACAGTGGCTCATGCCTGTAATCCCAACACTTTGGGAGGCTGAGGCGG 17972

NCF1C AA--GGGCCAGGCACAGTGGCTCATGCCTGTAATCCCAACACTTTGGGAGGCTGAGGCGG 17970

NCF1 ----GGGCCAGGCACAGTGGCTCATGCCTGTAATCCCAACACTTTGGGAGGCTGAGGTGG 17975 Pitx2

***************************************************** **

NCF1B GTGGATCTCCTGAGCTCAGGAGTTCAAGACCAGCCTGGCCAACAGGGTGATACCCCTTCT 18032

NCF1C GTGGATCTCCTGAGCTCAGGAGTTCAAGACCAGCCTGGCCAACAGGGTGATACCCCTTCT 18030

NCF1 GTGGATCTCCTGAGCTCAGGAGTTCAAGACCAGCCTGGCCAACAGGGTGATACCCCTTCT 18035 HNF4

************************************************************

NCF1B CTACTAAAAATACAAAATTAGCCAGGCGTGGTGGCGCACACCTATAATCCCAGCTACTTG 18092

NCF1C CTACTAAAAATACAAAATTAGCCAGGCGTGGTGGCGCACACCTGTAATCCCAGCTACTTG 18090

NCF1 CTACTAAAAATACAAAATTAGCCAGGCGTGGTGGCGCACACCTGTAATCCCAGCTACTTG 18095

******************************************* ****************

NCF1B GGAAGCTGAGGCAGGAGAATCGCTTGAACCTGGAAGGCAGAGGTTGCAGTGAGCCGAGAT 18152

NCF1C GGAAGCTGAGGCAGGAGAATCGCTTGAACCTGGAAGGCAGAGGTTGCAGTGAGCCGAGAT 18150

NCF1 GGAAGCTGAGGCAGGAGAATCGCTTGAACCTGGAAGGCAGAGGTTGCAGTGAGCCGAGAT 18155

************************************************************

NCF1B TGTGCCACTGCACTCCAGCCTGGGCAACAAGAGCGAAACTTCGCTTCAAACAAATAAATT 18212

NCF1C TGTGCCACTGCACTCCAGCCTGGGCAACAAGAGCGAAACTTCGCTTCAAACAAATAAATT 18210

NCF1 TGTGCCACTGCACTCCAGCTTGGGCAACAAGAGCGAAACTTCGCTTCAAACAAATAAATT 18215 SRY

******************* ****************************************

NCF1B AACGCCCAGCATGTCTTGGCTTTCATCTGCCAGACCTCAACCCTCACCCCCAGGAGATCA 18272

NCF1C AACGCCCAGCATGTCTTGGCTTTCATCTGCCAGACCTCAACCCTCACCCCCAGGAGATCA 18270

NCF1 AACGCCCAGCATGTCTTGGCTTTCATCTGCCAGACCTCAACCCTCACCCCCAGGAGATCA 18275

************************************************************

NCF1B GGTCCGGACCACGAGCTGACCCTGGACTCAGGCAAGGGTGAGTTGGTGCAGCCCTGGCCT 18332

NCF1C GGTCCGGACCACGAGCTGACCCTGGACTCAGGCAAGGGTGAGTTGGTGCAGCCCTGGCCT 18330

NCF1 GGTCCGGACCATGAGCTGACCCTGGACTCAGGCAAGGGTGAGTTGGTGCAGCCCTGGCCT 18335

*********** ************************************************

NCF1B GCTGGGAGGCACAGGCTGCAGCAGGCTGCCTGGGGCTGAGGCCCACCACTCATGAACTCA 18392

NCF1C GCTGGGAGGCACAGGCTGCAGCAGGCTGCCTGGGGCTGAGGCCCACCACTCATGAACTCA 18390

NCF1 GCTGGGAGGCACAGGCTGCAGCAGGCTGCCTGGGGCTGAGGCCCGCCACTCATGAACTCA 18395 HNF4

******************************************** ***************

NCF1B TGACCTTGAATGAGCTCCAAAAGCTCTGGGCCTCCCAGGCTCTAGGGGGAGTGGGAGAGA 18452

NCF1C TGACCTTGAATGAGCTCCAAAAGCTCTGGGCCTCCCAGGCTCTAGGGGGAGTGGGAGAGA 18450

NCF1 TGACCTTGAATGAGCTCCAAAAGCTCTGGGCCTCCCAGGCTCTAGGGGGAGTGGGAGAGA 18455 SF1

************************************************************

NCF1B GAGGCCTCAGCCTGTCCCTGGGCATGCTGCCCCCTCCTCACCTCTTTGTCCCAAATCCCC 18512

NCF1C GAGGCCTCAGCCTGTCCCTGGGCATGCTGCCCCCTCCTCACCTCTTTGTCCCAAATCCCC 18510

NCF1 GAGGCCTCAGCCTGTCCCTGGGCATGCTGCCCCCTCCTCACCTCTTTGTCCCAAATCCCC 18515 HNF4

************************************************************

NCF1B TTCCTGGCAAAGCTGACAGTCTTAATATCACTCTGGAGAAAACTGAGTCAGCCCTAAGGA 18572

NCF1C TTCCTGGCAAAGCTGACAGTCTTAATATCACTCTGGAGAAAACTGAGTCAGCCCTAAGGA 18570

NCF1 TTCCTGGCAAAGCTGACAGTCTTAATATCACTCTGGAGAAAACTGAGTCAGCCCTAAGGA 18575

************************************************************

NCF1B ACAATTCAATGAACCATTTGCTTACTTGAGGATTGGAACTCAAGTCTCACTCAAAGTCTG 18632

NCF1C ACAATTCAATGAACCATTTGCTTACTTGAGGATTGGAACTCAAGTCTCACTCAAAGTCTG 18630

NCF1 ACAATTCAATGAACCATTTGCTTACTTGAGGATTGGAACTCAAGTCTCACTCAAAGTCTG 18635 LEF1

************************************************************

NCF1B TGCCATTTTCGTCCCAGCTGTCACTGGCCCTCATCCACACACACCCAAGGATGAGCATCT 18692

NCF1C TGCCATTTTCGTCCCAGCTGTCACTGGCCCTCATCCACACACACCCAAGGATGAGCATCT 18690

NCF1 TGCCATTTTCGTCCCAGCTGTCACTGGCCCTCATCCACACACACCCAAGGATGAGCATCT 18695

************************************************************

NCF1B AACGCTTGCATGCACACTCCCATGCCCGCGTTCATTCACTCATTCATTCATTCATTCACT 18752

NCF1C AACGCTTGCATGCACACTCCCATGCCCGCGTTCATTCACTCATTCATTCATTCATTCACT 18750

NCF1 AACGCTTGCATGCACACTCCCATGCCCGCGTTCATTCACTCATTCATTCATTCATTCACT 18755

************************************************************

NCF1B CATTCATTGACTCATTCATTCATTCACTCACTCATTCATTCACTCAGTGAATGTTGCAGT 18812

NCF1C CATTCATTGACTCATTCATTCATTCACTCACTCATTCATTCACTCAGTGAATGTTGCAGT 18810

NCF1 CATTCATTGACTCATTCATTCATTCACTCACTCATTCATTCACTCAGTGAATGTTGCAGT 18815

************************************************************

NCF1B CACGATCCAAATATTTATGGCCTCTGTGTGCCAGGCACTAGCTGGAGGGGCTGGGGCTAG 18872

NCF1C CACGATCCAAATATTTATGGCCTCTGTGTGCCAGGCACTAGATGGAGGGGCTGGGGCTAG 18870

NCF1 CACGATCCAAATATTTATGGCCTCTGTGTGCCAGGCACTAGATGGAGGGGCTGGGGCTAG 18875

***************************************** ******************

NCF1B AGCCCCTGATAACCCGGTCATGCCCTAGCTTTCCTGGGACACACATTGTGGTAAGGGGAG 18932

NCF1C AGCCCCTGATAACCCGGTCATGCCCTAGCTTTCCTGGGACACACATTGTGGTAAGGGGAG 18930

NCF1 AGCCCCTGATAACCCGGTCATGCCCTAGCTTTCCTGGGACACACATTGTGGTAAGGGGAG 18935 AML1

************************************************************

NCF1B ACTAAAAAAATTAAGTCAGGCCAGGCACGGTGGCTCATGCCTGAATCCCAGCACTTTGGG 18992

NCF1C ACTAAAAAAATTAAGTCAGGCCAGGCACGGTGGCTCATGCCTGAATCCCAGCACTTTGGG 18990

NCF1 ACTAAAAAAATTAAGTCAGGCCAGGCACGGTGGCTCATGCCTGAATCCCAGCACTTTGGG 18995

************************************************************

NCF1B AGGCCGAGGCGAGTGAATTACCTGAGGTCAGGAGTTCAAGACCAGCCTGGCCAACATGGA 19052

NCF1C AGGCCGAGGCGAGTGAATTACCTGAGGTCAGGAGTTCAAGACCAGCCTGGCCAACATGGA 19050

NCF1 AGGCCGAGGCGAGTGAATTACCTGAGGTCAGGAGTTCAAGACCAGCCTGGCCAACATGGA 19055 HNF4

************************************************************

NCF1B GAAACCCAGTCTCTAATTAAAAAAAAAAAAAAAATTAGCCAGGTGTGGTGGCACATGCCT 19112

NCF1C GAAACCCAGTCTCTAATTAAAAAAAAAAAAAAAATTAGCCAGGTGTGGTGGCACATGCCT 19110

NCF1 GAAACCCAGTCTCTAATTAAAAAAAAAAAAAA--TTAACCAGGTGTGGTGGCACATGCCT 19113 AML1

******************************** *** **********************

NCF1B GTAATCCCAGCTACTCAGGAGACTAACGCAAGAGAATTGCTTGAACCCAGGAGGCAGAGG 19172

NCF1C GTAATCCCAGCTACTCAGGAGACTAACGCAAGAGAATTGCTTGAACCCAGGAGGCAGAGG 19170

NCF1 GTAATCCCAGCTACTCAGGAGACTAACGCAAGAGAATTGCTTGAACCCAGGAGGCAGAGG 19173

************************************************************

NCF1B TTGCGGTGAGCCGAGATCGCGCCATTGCACTCCAGCCTGGGAAACAAGAGCGAGACTCCA 19232

NCF1C TTGCGGTGAGCCGAGATCGCGCCATTGCACTCCAGCCTGGGAAACAAGAGCGAGACTCCA 19230

NCF1 TTGCGGTGAGCCGAGATCGCGCCATTGCACTCCAGCCTGGGAAACAAGAGCGAGACTCCA 19233 E2F, FoxM1

************************************************************

NCF1B TCTCAAAAAAAAAAAAA--GTGGGAGGCAGAGGCAGGAGGATCACTAGAGGCCAGTAGTT 19290

NCF1C TCTCAAAAAAAAAAAAAAAGTGGGAGGCAGAGGCAGGAGGATCACTAGAGGCCAGTAGTT 19290

NCF1 TCTCAAAAAAAAAAAAG---TGGGAGGCAGAGGCAGGAGGATCACTAGAGGCCAGTAGTT 19290

**************** ****************************************

NCF1B TGAGACCATCCTGGGCAACATAGCAGGACCCTGTCTGTACAAAAAAATTAAAAAAAATTT 19350

NCF1C TGAGACCATCCTGGGCAACATAGCAGGACCCTGTCTGTACAAAAAAATTAAAAAAAATTT 19350

NCF1 TGAGACCATCCTGGGCAACATAGCAGGACCCTGTCTGTACAAAAAAATTAAAAAAAATTT 19350

************************************************************

NCF1B AACCGGGCATGGTGGCACACACCCGTAGTCCCAGCTACTCCAGAGGCTGAGGCAGGAGGA 19410

NCF1C AACCGGGCATGGTGGCACACACCCGTAGTCCCAGCTACTCCAGAGGCTGAGGCAGGAGGA 19410

NCF1 AACCGGGCATGGTGGCACACACCCGTAGTCCCAGCTACTCCAGAGGCTGAGGCAGGAGGA 19410

************************************************************

NCF1B TCGCTGGAGCCCAGGAGTTGGAGGCTGCAGTGAACTGTGATCCCACCACTGCGCTTAAGC 19470 HNF4

NCF1C TCGCTGGAGCCCAGGAGTTGGAGGCTGCAGTGAACTGTGATCCCACCACTGCGCTTAAGC 19470

NCF1 TCGCTGGAGCCCAGGAGTTGGAGGCTGCAGTGAACTGTGATCCCACCACTGCACTTAAGC 19470

**************************************************** *******

NCF1B CTGGATAACAAAGCAAGACCCTGTCTCAAATAACAATAGCAATAATAATAAAGAAAAATT 19530

NCF1C CTGGATAACAAAGCAAGACCCTGTCTCAAATAACAATAGCAATAATAATAAAGAAAAATT 19530

NCF1 CTGGATAACAAAGCAAGACCCTGTCTCAAATAACAATAGCAATAATAATAAAGAAAAATT 19530

************************************************************

NCF1B AAATGCAATTTGCGATGCATCAGTGATAAGTGCTCTGCAGAAAAAGGAGGCAGGAAGAGG 19590

NCF1C AAATGCAATTTGCGATGCATCAGTGATAAGTGCTCTGCAGAAAAAGGAGGCAGGAAGAGG 19590

NCF1 AAATGCAATTTGCGATGCATCAGTGATAAGTGCTCTGCAGAAAAAGGAGGCAGGAAGAGG 19590

************************************************************

NCF1B CTGAGAAAGGTATGAGGTTTGCTATGCAATGTGAAGTTATCAAGGAAGGCTTCTCGGAAG 19650

NCF1C CTGAGAAAGGTATGAGGTTTGCTATGCAATGTGAAGTTATCAAGGAAGGCTTCTCGGAAG 19650

NCF1 CTGAGAAAGGTATGAGGTTTGCTATGCAATGTGAAGTTATCAAGGAAGGCTTCTCGGAAG 19650

************************************************************

NCF1B AGGTGACATTTGAGCAGAGAAATGGAGGAGAGTTATGGAGGGAAGATGGTGAATGGGGGG 19710

NCF1C AGGTGACATTTGAGCAGAGAAATGGAGGAGAGTTATGGAGGGAAGATGGTGAATGGGGGG 19710

NCF1 AGGTGACATTTGAGCAGAGAAATGGAGGAGAGTTATGGAGGGAAGATGGTGAATGGGGGG 19710

************************************************************

NCF1B AACATGGTCAAGACCAGGAATATGGTCAAGGGGGGAAAGATGGTCAAGGGGACGCAGCAA 19770

NCF1C AACATGGTCAAGACCAGGAATATGGTCAAGGGGGGAAAGATGGTCAAGGGGACGCAGCAA 19770

NCF1 AACATGGTCAAGACCAGGAATATGGTCAAGGGGGGAAAGATGGTCAAGGGGACGCAGCAA 19770

************************************************************

NCF1B ATGCAAAGGCCCTGAGGCAGGAGCAGCTTGATTCACCCCCAAAACCCGTGGGGCCCGTGC 19830

NCF1C ATGCAAAGGCCCTGAGGCAGGAGCAGCTTGATTCACCCCCAAAACCCGTGGGGCCCGTGC 19830

NCF1 ATGCAAAGGCCCTGAGGCAGGAGCAGCTTGATTCACCCCCAAAACCCGTGGGGCCCGTGC 19830

************************************************************

NCF1B AGGCGACGGGAAGGACAAGTGTAAACCCTTTTCCTTGTCCCTGCAGGTGTGTGTGAACAT 19890

NCF1C AGGCGACGGGAAGGACAAGTGTAAACCCTTTTCCTTGTCCCTGCAGGTGTGTGTGAACAT 19890

NCF1 AGGCGACGGGAAGGACAAGTGTAAACCCTTTTCCTTGTCCCTGCAGGTGTGTGTGAACAT 19890

************************************************************

NCF1B GAGTCTGCCCATGTTTACACCCTGCAAGCCTGAAGAGTCCCCAGAAACTGAAAGAAGAAG 19950

NCF1C GAGTCTGCCCATGTTTACACCCTGCAAGCCTGAAGAGTCCCCAGAAACTGAAAGAAGAAG 19950

NCF1 GAGTCTGCCCATGTTTACACCCTGCAAGCCTGAAGAGTCCCCAGAAACTGAAAGAAGAAG 19950

************************************************************

NCF1B CAAAGCCCTTTCTGTACCCTCCCTGCCCCCTGTCCCGACCGCGACAAAAG 20000

NCF1C CAAAGCCCTTTCTGTACCCTCCCTGCCCCCTGTCCCGACCGCGACAAAAG 20000

NCF1 CAAAGCCCTTTCTGTACCCTCCCTGCCCCCTGTCCCGACCGCGACAAAAG 20000

**************************************************

References in Supplementary Figure 6:

Larkin MA, Blackshields G, Brown NP, Chenna R, McGettigan PA, McWilliam H, Valentin F, Wallace IM, Wilm A, Lopez R, Thompson JD, Gibson TJ, Higgins DG: **ClustalW and ClustalX version 2.** *Bioinformatics* 2007, 23(21): 2947-2948*.*

Loots G, Ovcharenko I, Pachter L, Dubchak I, Rubin E: **rVISTA for comparative sequence-based discovery of functional transcription factor binding sites.** *Genome Res.* 2002, 12:832-839.
